# Supplementary material for: Haplotype editing with CRISPR-Cas9 as a therapeutic approach for dominant-negative missense mutations in NEFL
Source: Mol Ther. 2025 Nov 19;34(3):1633–51. doi: 10.1016/j.ymthe.2025.11.026 (PMC12974165; doi:10.1016/j.ymthe.2025.11.026)
Supplement: Document S1. Figures S1–S20 and Tables S1–S10 [file mmc1.pdf]

YMTHE, Volume *34*

Supplemental Information

Haplotype editing with CRISPR-Cas9 as a therapeutic approach for dominant-negative missense mutations in *NEFL*

Poorvi H. Dua, Bazilco M.J. Simon, Chiara B.E. Marley, Carissa M. Feliciano, Hannah L. Watry, Quinn T. Cowan, Dylan Steury, Abin Abraham, Erin N. Gilbertson, Grace D. Ramey, John A. Capra, Bruce R. Conklin, and Luke M. Judge

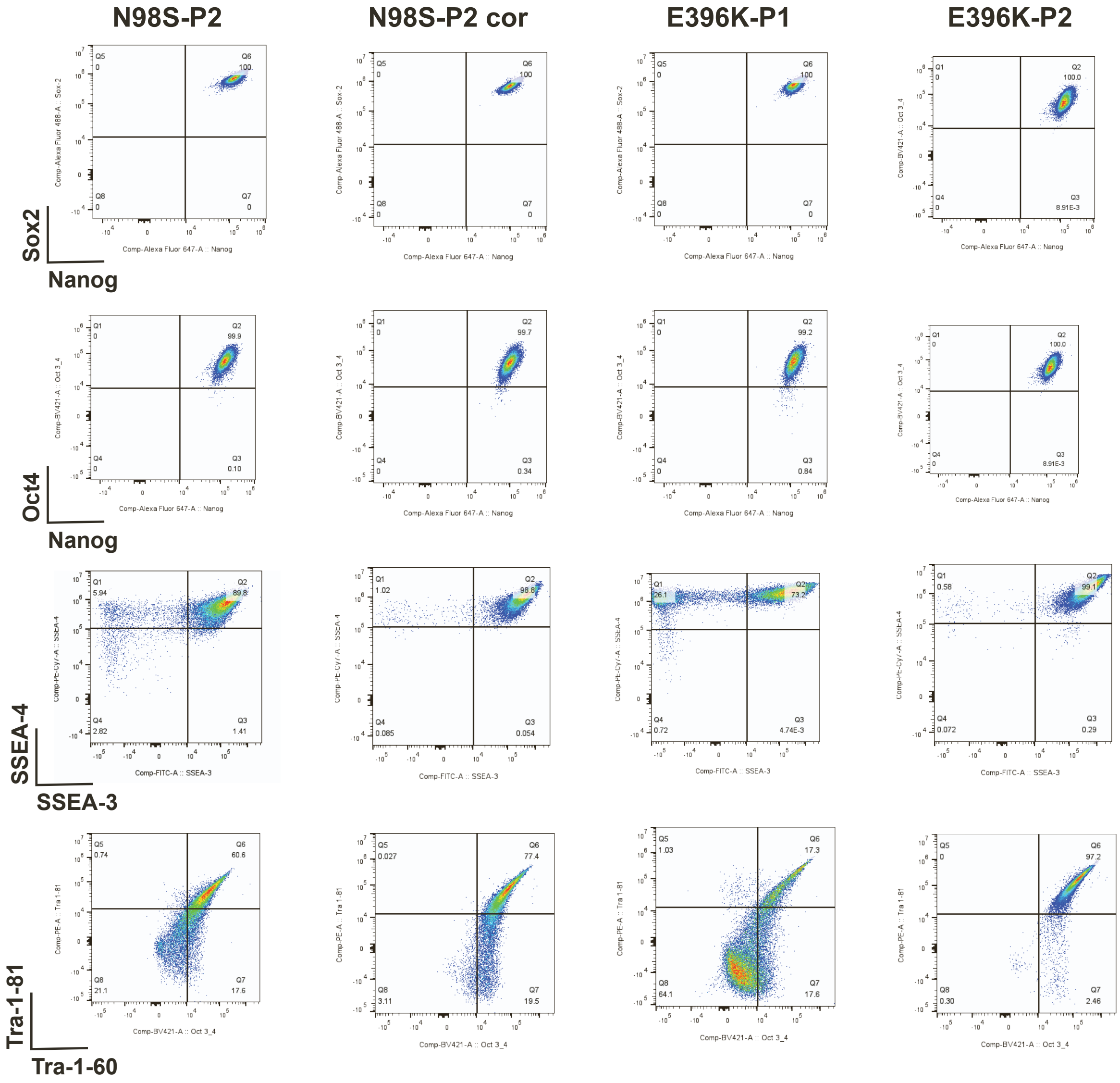

Figure S1: Expression of pluripotency markers in iPSC. Patient-derived iPSC containing the CLYBL-hNIL transgene (N98S-P2, E396K-P1, and E396K-P2) and the isogenic corrected version of N98S-P2 (N98S-P2-cor) were stained with a live/dead marker and antibodies to Sox2, Nanog, Oct 3/4, SSEA-3, SSEA-4, Tra-1-60, and Tra-1-81 (see Table S7 for list of antibodies). Flow cytometry was used to analyze expression of each marker in live cells.

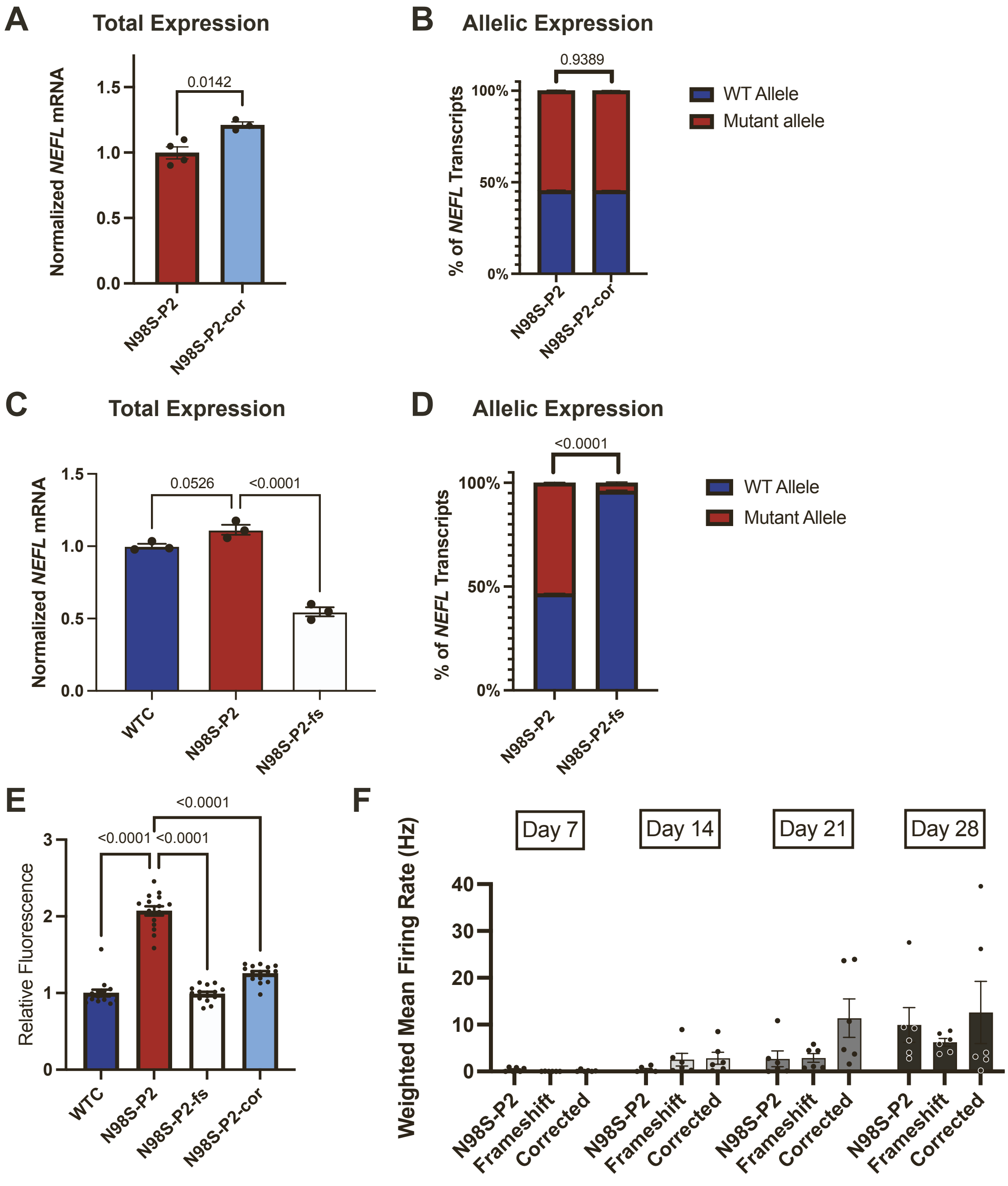

Figure S2: Derivation and characterization of clonal edited iPSC lines derived from N98S-P2 patient line. Transfection of N98S-P2 iPSC with N98S-specific HiFiCas9 RNP produced N98S-P2-fs (contains +1 insertion at the mutation locus). Transfection of N98S-P2 iPSC with N98S-specific HiFiCas9 RNP plus single-strand oligonucleotide donor produced N98S-P2-cor (precise correction of N98S mutation with linked silent mutation to facilitate

genotyping). Clonal iPSC were differentiated into i^{3}LMNs and RNA was extracted on day 7. **(A,C)** Total *NEFL* expression by quantitative RT-ddPCR, normalized to GAPDH. **(B,D)** Relative allelic expression by allele discrimination RT-ddPCR. Bar graphs for A-D represent mean +/- S.E.M. with each data point representing an independent well of i^{3}LMN (n = 3). Statistical comparisons by unpaired two-tailed T-test in (A,B,D) and one-way ANOVA with Dunnett's test for comparison of multiple lines to N98S-P2 in (C). **(E)** Mean NfL fluorescence intensity in the cell bodies of day 7 i^{3}LMNs. Bar graph represents mean +/- S.E.M. of 15 independent wells of i^{3}LMN with each data point representing the mean value from 5 images per well. Statistical comparisons by one-way ANOVA with Dunnett's test for comparison of multiple lines to N98S-P2. **(F)** Day 3 i^{3}LMNs were seeded on multi-electrode array plates to measure spontaneous action potentials in N98S-P2, corrected, and frameshift lines. Spontaneous electrical activity was measured at days 7, 14, 21, and 28. Bar graphs represent mean +/- S.E.M. with each data point representing the mean firing rate from an independent well of i^{3}LMN (n = 6). Statistical comparison by two-way ANOVA demonstrated a statistically significant effect of the differentiation day (p = 0.0002) but no significant difference between cell lines (p = 0.0881).

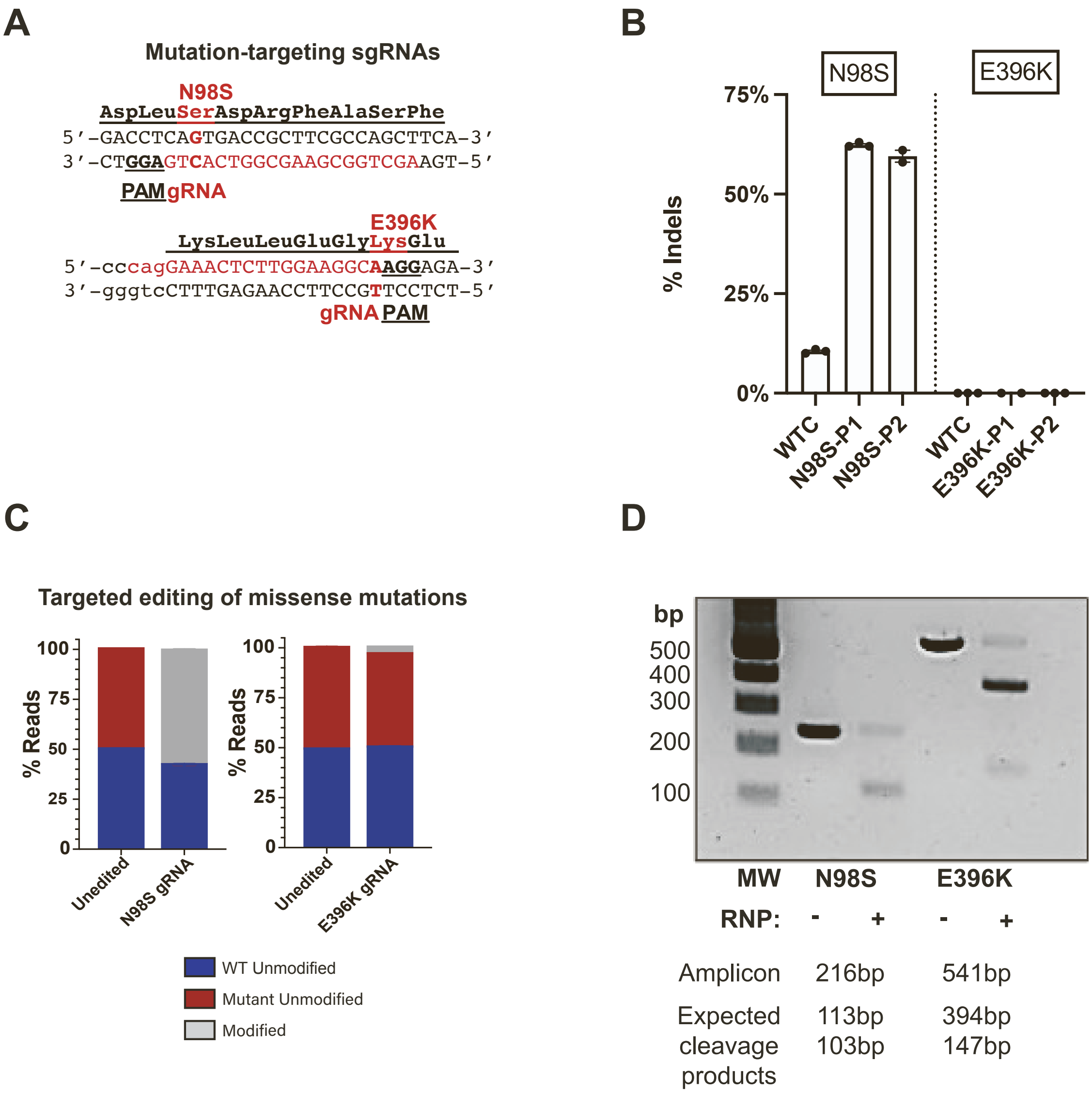

Figure S3: Comparison of the editing and nuclease activity of gRNAs targeting the N98S and E396K mutations. (A) Sequences of N98S and E396K mutation-targeting gRNAs (red) with associated PAM sequences (underlined). **(B)** Indel editing efficiency of N98S and E396K mutation-specific gRNAs in multiple patient backgrounds by Sanger sequencing and ICE analysis. Bar graphs represent mean +/- S.E.M with each data pointing indicating an independent transfection (n = 2 – 3). **(C)** NGS amplicon sequencing of N98S and E396K targeted editing in N98S-P2 (left) and E396K-P2 (right) iPSCs. Modified reads contain indels at the target site, WT and mutant unmodified indicate reads without indels on the WT and mutant

alleles, respectively. Bar graphs represent mean +/- S.E.M of independent transfections (n = 3 for N98S, n = 6 for E396K). **(D)** PCR amplicons spanning the N98S or E396K mutations were generated from N98S-P2 and E396K-P2 gDNA, respectively, followed by incubation with corresponding N98S or E396K mutation-specific gRNA and HiFiCas9 RNP. Gel electrophoresis identifies the expected cleavage products. MW = molecular weight DNA ladder.

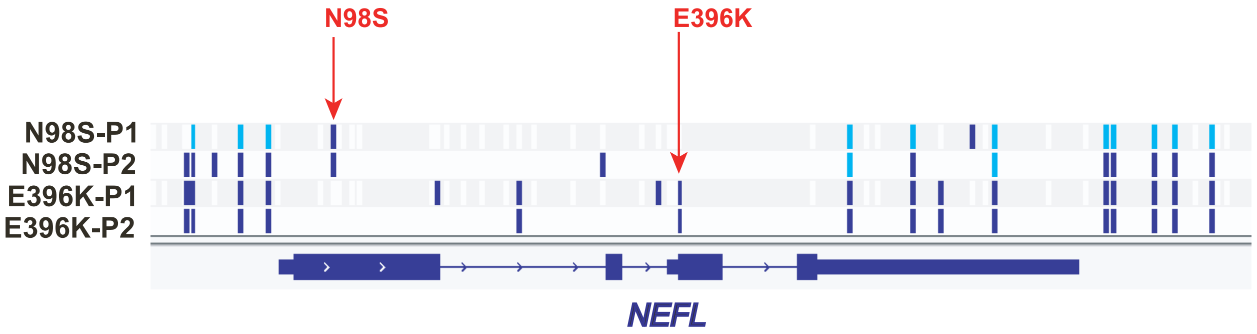

Figure S4: Panel of CMT2E patient iPSC lines with variants flanking the *NEFL* coding region. Whole genome sequencing was performed from patient-derived iPSC. Dark blue lines indicate heterozygous variants and light blue lines indicate homozygous variants. Causative missense mutations are annotated with red arrows. Images generated from vcf files using Integrative Genomics Viewer[1].

1. Robinson, J.T., Thorvaldsdóttir, H., Winckler, W., Guttman, M., Lander, E.S., Getz, G., and Mesirov, J.P. (2011). Integrative genomics viewer. Nat. Biotechnol. *29*, 24–26. 10.1038/nbt.1754.

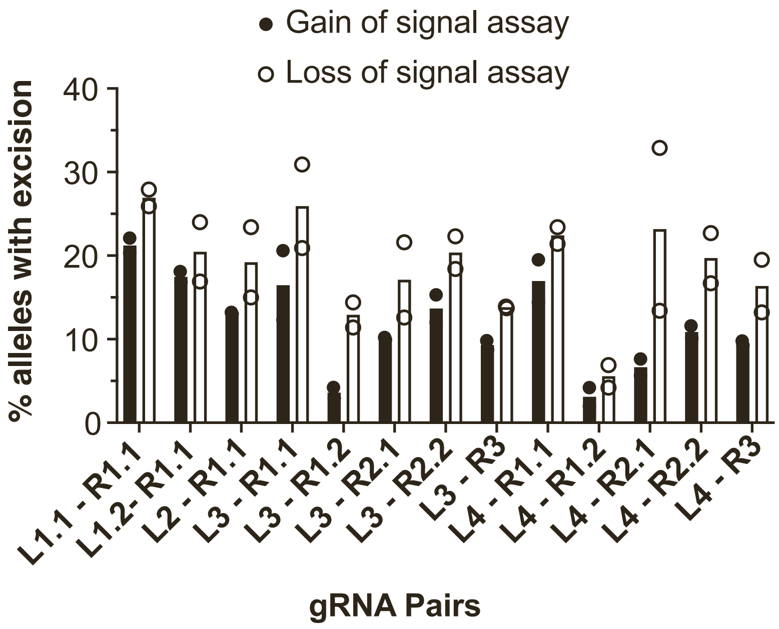

Figure S5: Comparison of ddPCR assays to measure excision by gain or loss of signal. N98S-P2 iPSC were nucleofected with different pairs of gRNA-HiFiCas9 RNP in duplicate, followed by gDNA extraction 4 days later. Excision was quantified for each sample using the assay shown in Figure 1E designed to detect the specific expected excision event (gain of signal, filled circles) and separately using an *NEFL* internal primer/probe assay that would be disrupted by gene excision or any event leading to loss of the genomic locus (loss of signal, open circles). Both assays were normalized to an internal RPP30 genomic copy number assay. For the loss of signal assay, percent excision was calculated by subtracting the normalized *NEFL* signal for each edited sample from unedited N98S-P2 control samples. Bar graphs represent mean values with data points indicating independent duplicate transfections.

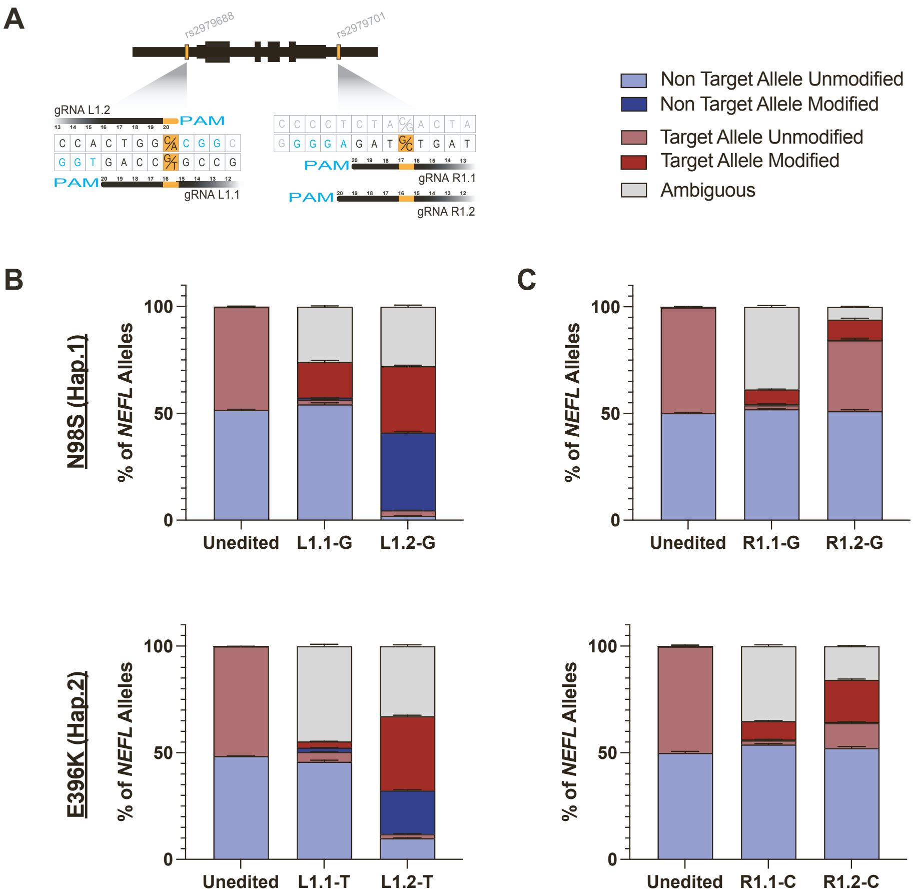

Figure S6: Analysis of allele-specific editing with single gRNA. (A) Schematic of sequences targeted by SNP-specific gRNAs with variants in orange and the associated PAM in blue. **(B,C)** N98S-P2 (top) and E396K-P2 (bottom) iPSC were transfected with the indicated allele-specific gRNA-HiFiCas9 RNPs followed by quantification of editing outcomes by NGS amplicon sequencing and CRISPResso2 analysis. Ambiguous editing events cannot be assigned to either allele due to the deletion of the variant nucleotide. Bar graphs represent the mean +/- S.E.M. of independent transfections (n = 3).

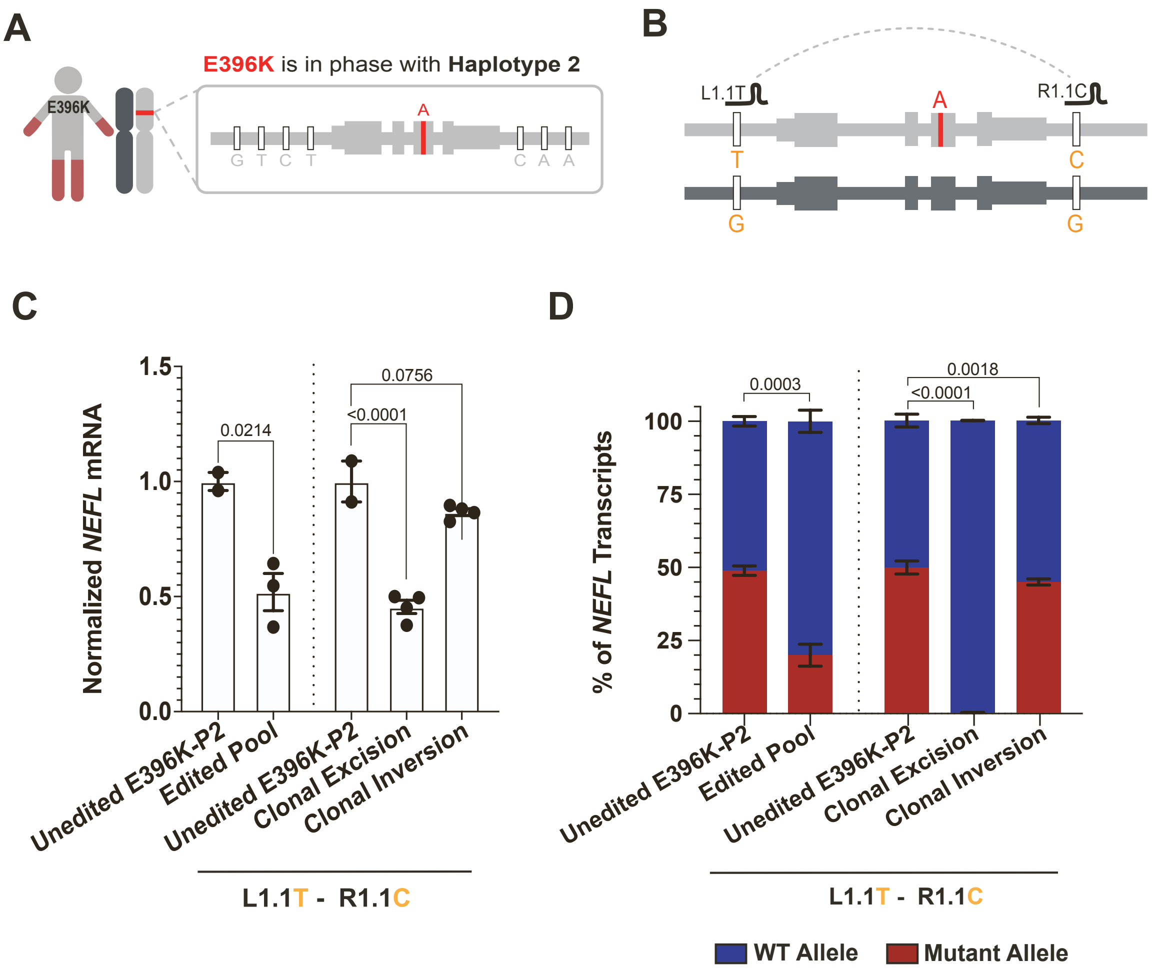

Figure S7: Analysis of *NEFL* gene expression after haplotype editing in E396K-P2 i^{3}LMNs. **(A)** Schematic of phasing of E396K mutation with haplotype 2 **(B)** Schematic of SNP-targeting gRNA pair for E396K haplotype editing. Edited and unedited iPSCs from E396K-P2 were differentiated into i^{3}LMNs, and RNA was extracted on Day 7. **(C)** Total *NEFL* expression was measured by quantitative RT-ddPCR relative to *GAPDH* and normalized to the unedited control. **(D)** Relative expression of wildtype and mutant alleles was measured by allele discrimination ddPCR. Bar graphs represent mean +/- S.E.M. normalized to unedited control, with each data point representing an independent well of i^{3}LMNs (n = 2 – 4). Dashed vertical lines indicate separate experiments for mixed population of edited cells versus clones with specific editing outcomes. Comparisons were performed by unpaired two-tailed T-test; multiple comparisons were performed by one-way ANOVA with Dunnett's post-test.

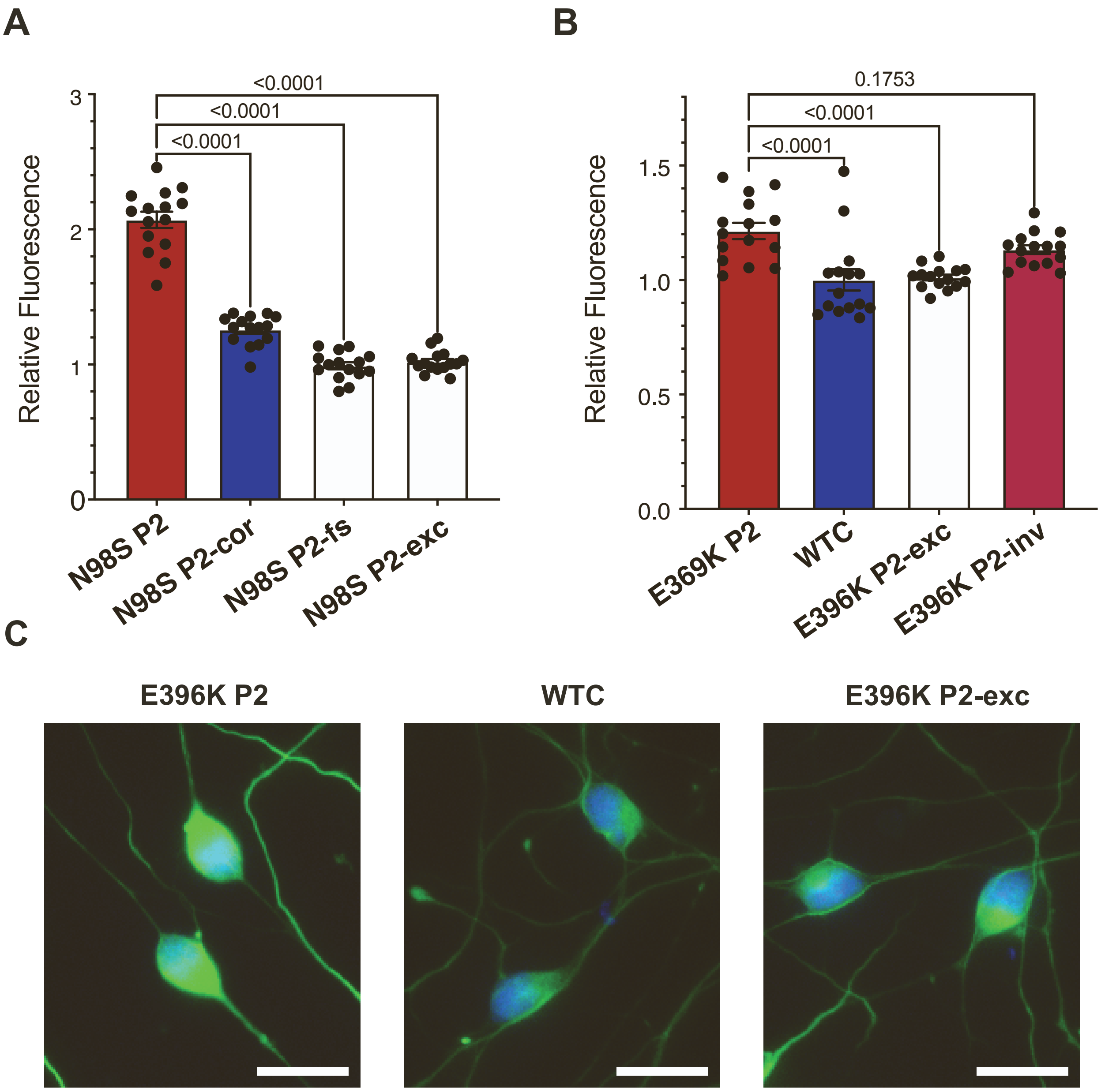

Figure S8: NfL accumulation in cell bodies of control and clonal edited i³LMNs.

Quantification of mean NF-L fluorescence intensity in inferred cell bodies in day 7 clonal edited i³LMNs derived from **(A)** N98S-P2 and **(B)** E396K-P2. Bar graph represents mean +/- S.E.M. of 15 independent wells of i³LMNs with each data point representing the mean NfL intensity from 5 images per well. Statistical comparisons by one-way ANOVA with Dunnett's post-test. **(C)** Representative images of day 7 i³LMNs stained with anti-NF-L (green) and anti-HB9 (blue). Scale bars = 20 μM.

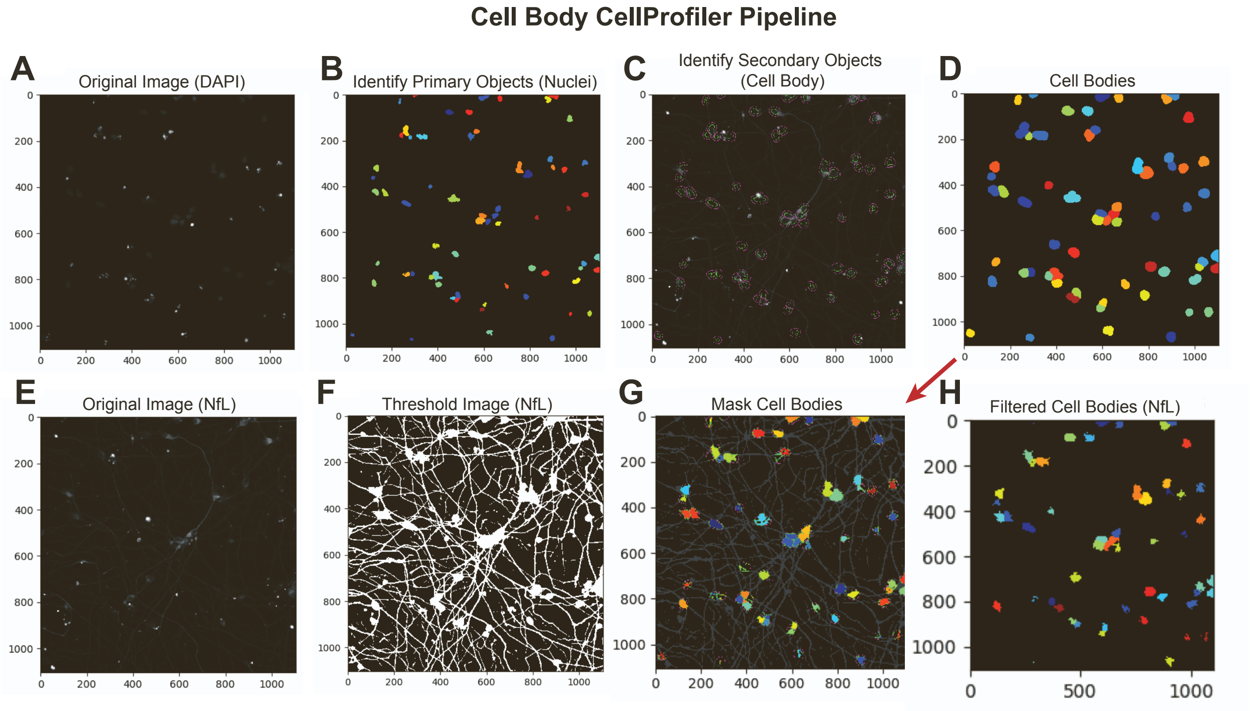

Figure S9: CellProfiler pipeline for measuring NfL intensity in i³LMN cell bodies. **(A-B)** Nuclei (primary objects) were identified from the DAPI-stained image channel. **(C-D)** Cell bodies (secondary objects) were inferred by extending the area of DAPI+ signal by 10 pixels. **(E-F)** The NfL-stained image channel was thresholded and then **(G)** masked with the inferred cell bodies. **(H)** The cell bodies were then filtered by shape and compactness to remove any incorrectly identified objects. Red arrow indicates the application of masks to images in other channels. The mean intensity in the cell body-masked NfL-stained image channel was measured to quantify NfL within cell bodies.

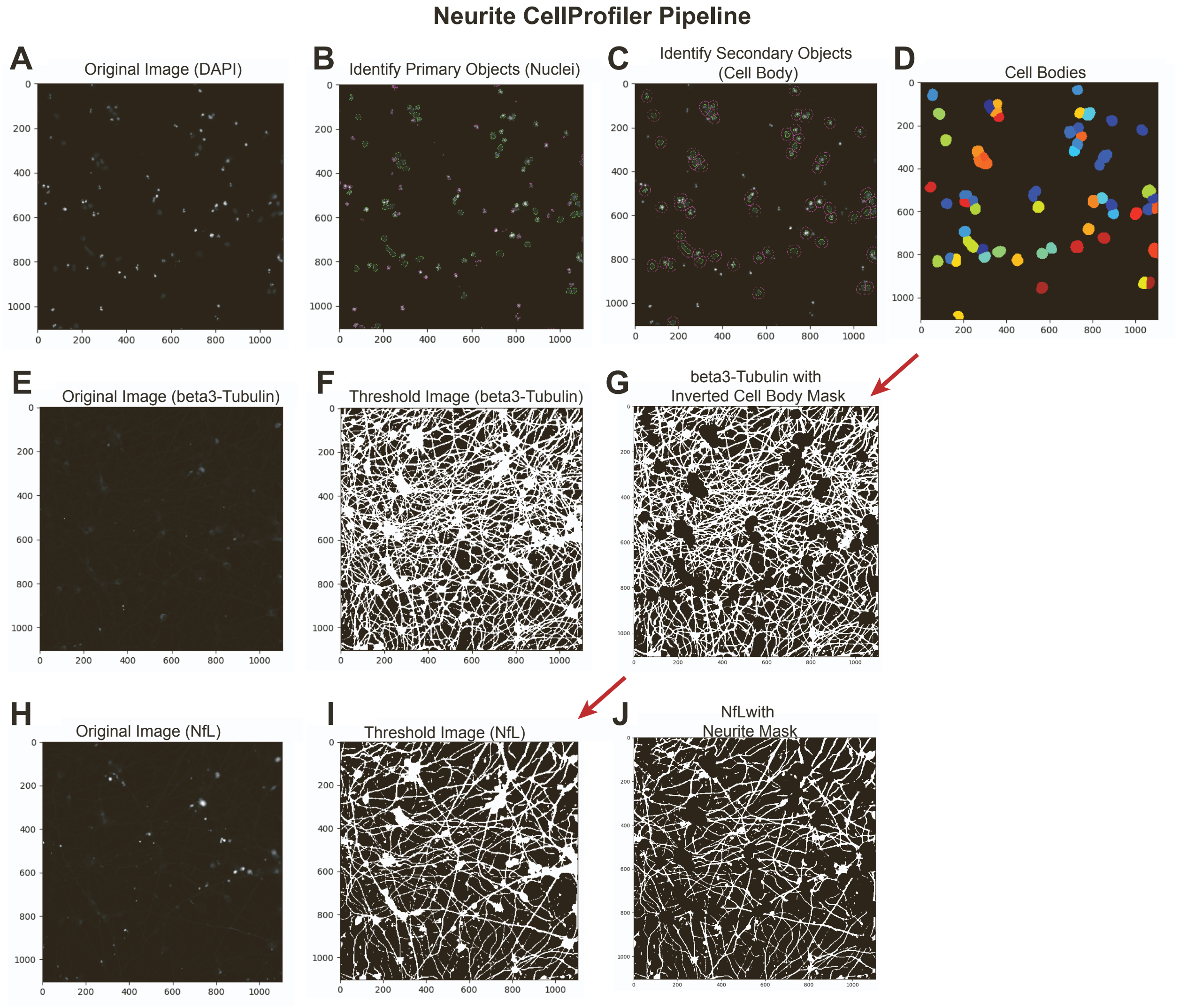

Figure S10: CellProfiler pipeline for measuring NF-L intensity in i^{3}LMN neurites.

(A-B) Nuclei (primary objects) were identified from the DAPI-stained image channel. **(C-D)** Cell bodies (secondary objects) were inferred by extending the area of the DAPI+ signal by 15 pixels. **(E-F)** The beta3-tubulin-stained image channel was thresholded, and **(G)** an inverted cell body mask was applied to define the region encompassed by neurites. **(H-I)** The NfL-stained image channel was then thresholded and **(J)** masked with the beta3-tubulin neurite image. Red arrows indicate the application of masks to images in other channels. Total intensity in the neurite-masked NfL-stained image channel was calculated and divided by the beta3-tubulin-positive neurite area to normalize for variation in neurite density.

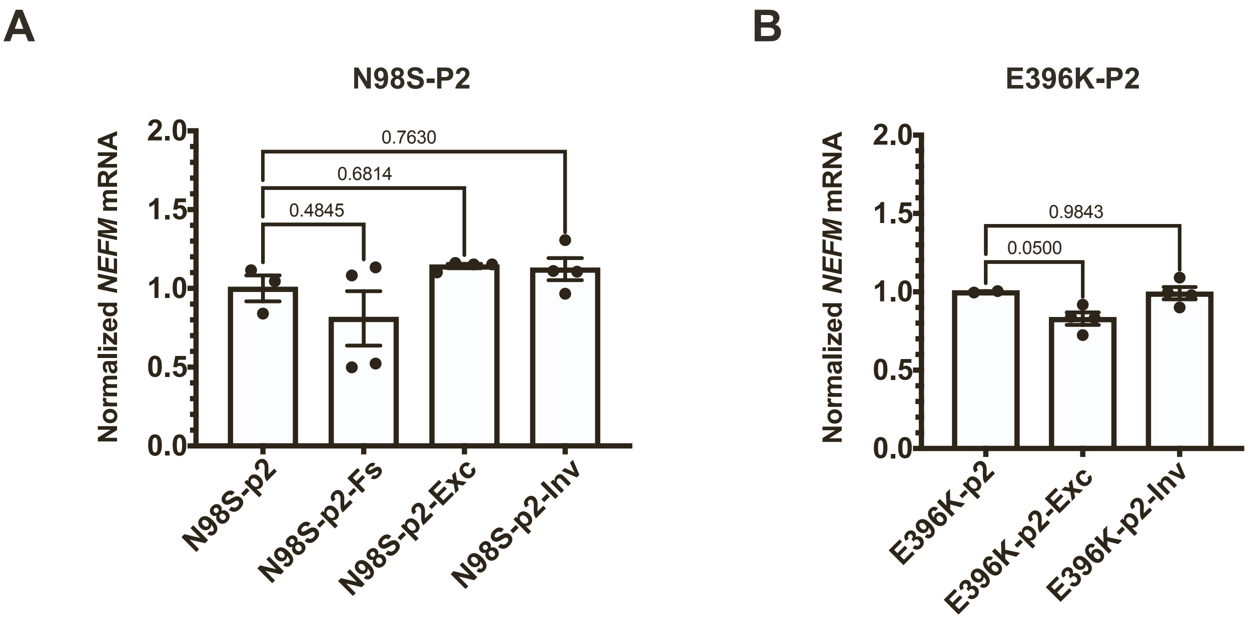

Figure S11: Total *NEFM* expression in edited i[3]LMNs. Edited iPSCs from **(A)** N98S-P2 and **(B)** E396K-P2 were differentiated into i[3]LMNs, and RNA was extracted on day 7. Total *NEFM* expression was measured by quantitative RT-ddPCR relative to *GAPDH* and normalized to unedited controls. Bar graphs represent mean +/- S.E.M. normalized to unedited control, with each data point representing an independent well of i[3]LMNs (n = 2 – 4). Multiple comparisons were performed by one-way ANOVA with Dunnett's post-test.

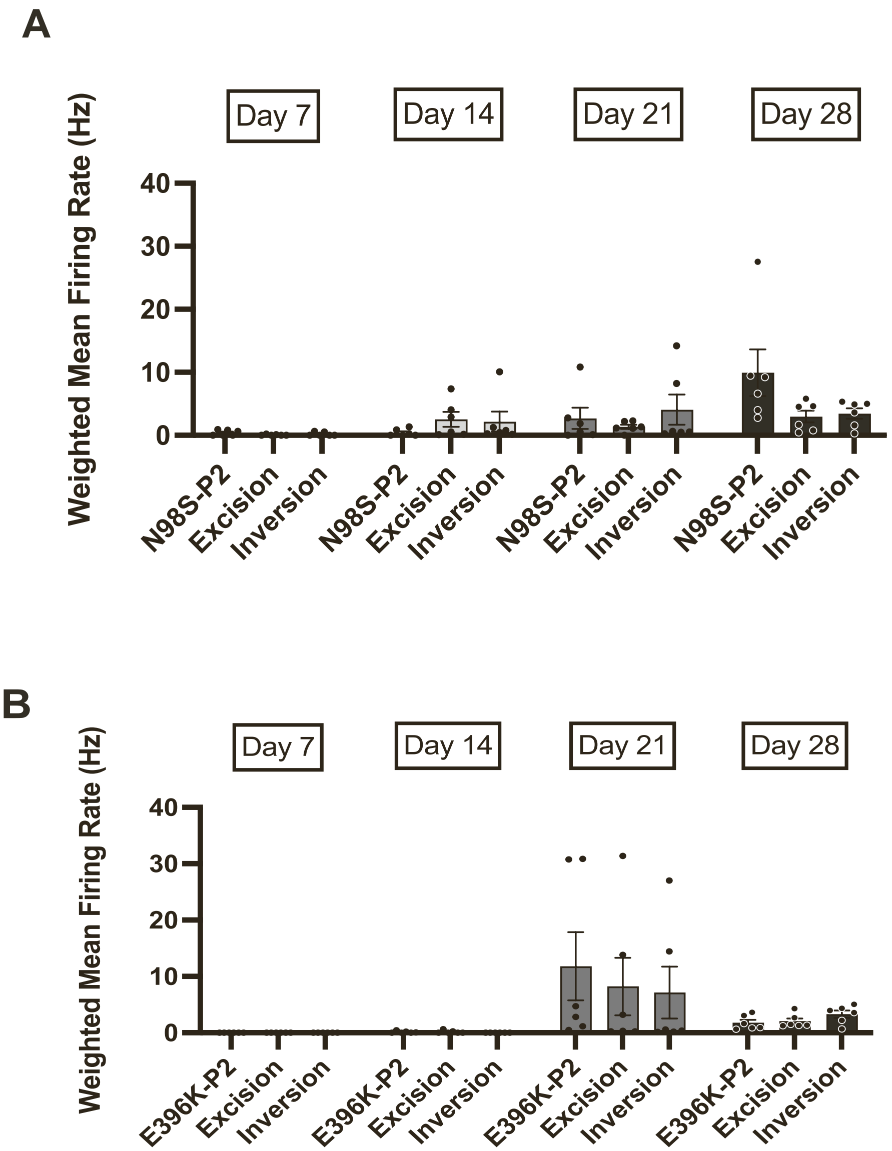

Figure S12: Total *NEFL* gene excision and inversion do not impair i³LMN electrophysiology. Day 3 i³LMNs were seeded on multi-electrode array plates to measure spontaneous action potentials on days 7, 14, 21, and 28 for **(A)** N98S-P2 and **(B)** E396K-P2 series of edited i³LMN. Bar graphs represent mean +/- S.E.M. with each data point representing the mean firing rate from an independent well of i³LMN (n = 6). Two-way ANOVA demonstrated a statistically significant effect of the differentiation day (N98S-P2 series: p = 0.0009, E396K-P2 series: p = 0.0002) but no significant difference between cell lines (N98S-P2 series: p = 0.3258, E396K-P2 series: p = 0.8814).

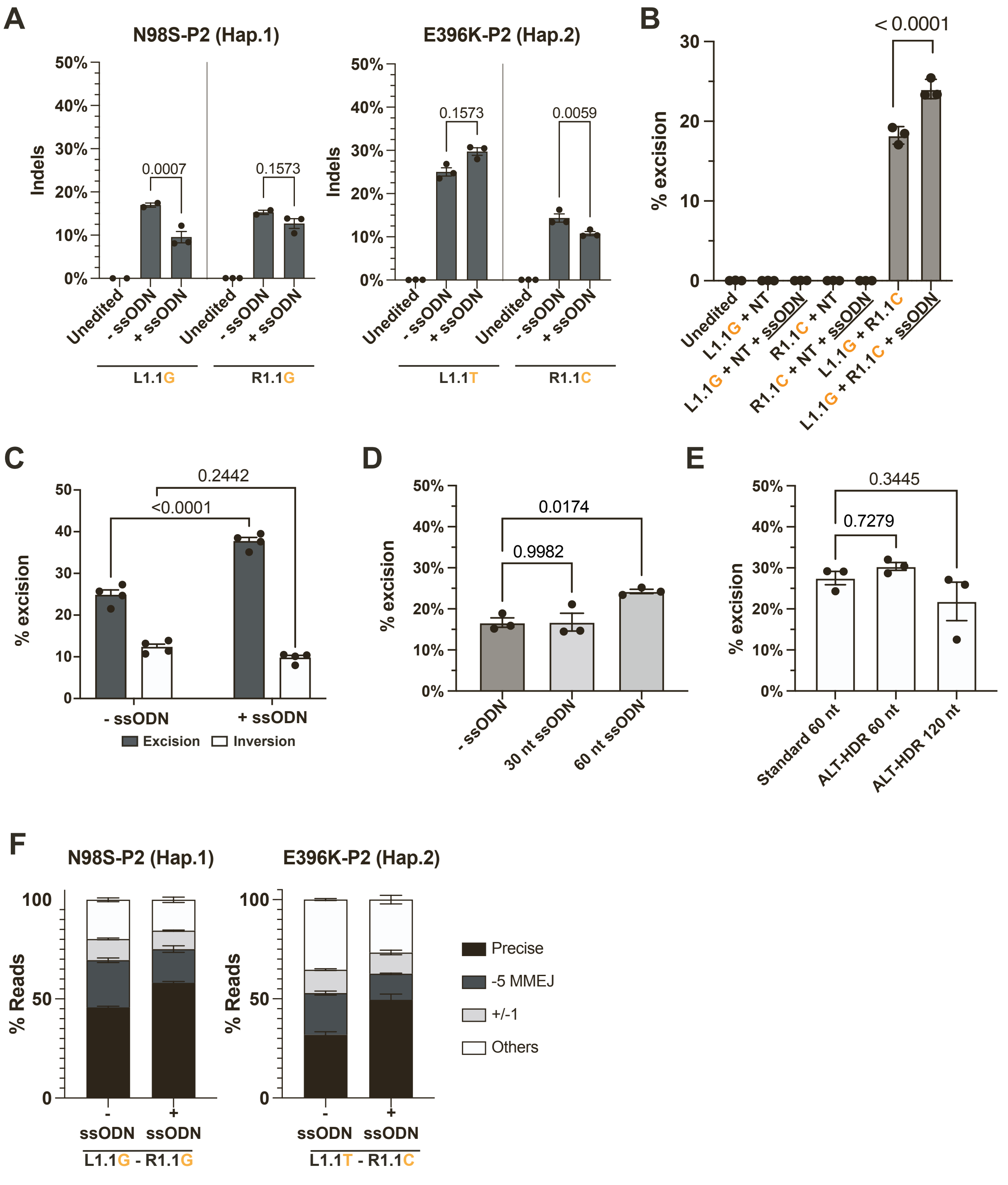

Figure S13: Effect of bridging ssODN on editing outcomes in iPSCs. (A) N98S-P2 and E396K-P2 iPSCs were nucleofected with L1.1 and R1.1 RNP with and without the addition of a 60 nt single-strand oligonucleotide donor (ssODN). PCR amplicons spanning L1.1 and R1.1 target sites were analyzed by NGS amplicon sequencing to quantify indels on alleles that were

not edited by excision or inversion. **(B)** E396K-P2 iPSC were nucleofected with L1.1 and R1.1 RNP, and with replacement of L1.1 or R1.1 with a non-targeting (NT) gRNA, with and without ssODN. Excision frequency was measured by ddPCR. **(C)** N98S-P2 iPSCs were transfected with L1.1 + BA1 RNP with and without ssODN. Excision and inversion frequency were measured by ddPCR. **(D,E)** E396K-P2 iPSCs were transfected with L1.1 + R1.1 RNP and ssODN of different lengths and chemistries. Excision was measured by ddPCR. Comparison of 30 and 60 nt unmodified ssODN shown in (D). Comparison of unmodified (60 nt) and ALT-HDR (Integrated DNA Technologies) modified (60 and 120 nt) ssODN shown in (E). For all experiments, gDNA was collected four days after nucleofection. For (A-E) bar graphs represent mean +/- S.E.M. with individual data points representing independent transfections (n = 3 – 4). Multiple comparisons were done by one- or two-way ANOVA with (A,B) Sidak's, (C) Tukey's, and (D,E) Dunnet's post-tests. **(F)** N98S-P2 (left) or E396K-P2 (right) iPSC were nucleofected with L1.1 and R1.1 RNP with and without the addition of a 60 nt single-strand oligonucleotide donor (ssODN). Excision events were amplified by PCR and sequenced by NGS. Outcomes were categorized as the expected excision repair (precise), 5-nucleotide larger than expected excision which was presumed microhomology-mediated (-5 MMEJ), single nucleotide insertion or deletion at the excision junction (+/-1), or numerous other rare variations (other). Statistical comparisons for the effect of ssODN on each category was performed by two-way ANOVA followed by Sidak's post-test as follows:

N98S-P2 - precise: $p < 0.0001$, -5 MMEJ: $p = 0.0008$, +/-1: $p = 0.8281$

E396K-P2 - precise: $p < 0.0001$, -5 MMEJ: $p = 0.0072$, +/-1: $p = 0.9777$

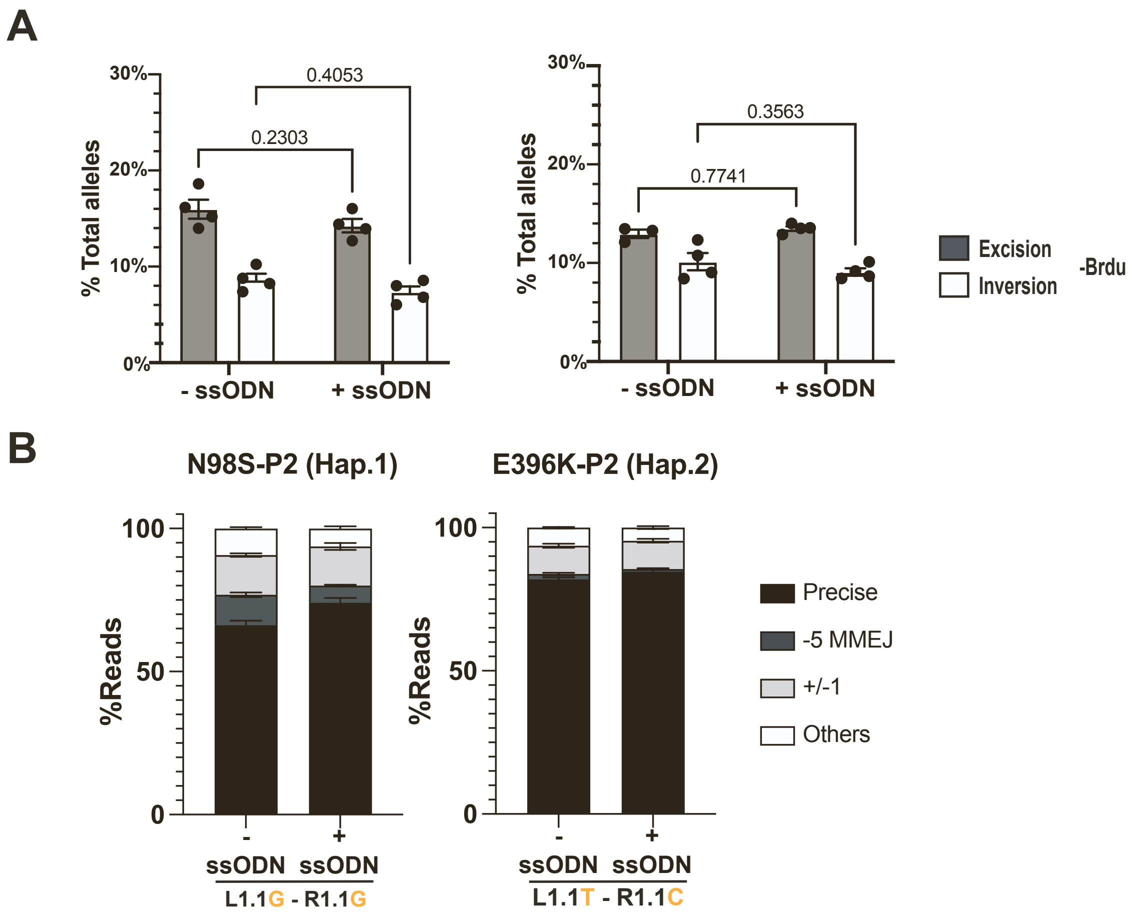

Figure S14: Effect of bridging ssODN on editing outcomes in day 3 i[3]LMNs. (A) N98S-P2 (left) and E396K-P2 (right) day 3 i[3]LMNs were nucleofected with L1.1 and R1.1 RNP with and without the addition of a 60 nt single-strand oligonucleotide donor (ssODN). Brdu was excluded from the media from days 3-4. Excision and inversion frequency were measured by ddPCR. Multiple comparisons were performed by two-way ANOVA with Sidak's post-test. **(B)** Excision events were amplified by PCR and sequenced by NGS. Outcomes were categorized as the expected excision repair (precise), 5-nucleotide larger than expected excision which was presumed microhomology-mediated (-5 MMEJ), single nucleotide insertion or deletion at the excision junction (+/-1), or numerous other rare variations (other). Bar graphs represent mean +/- S.E.M. of independent transfections (n = 4). Statistical comparisons for the effect of ssODN on each category was performed by two-way ANOVA followed by Sidak's post-test as follows: N98S-P2 - precise: $p < 0.0001$, -5 MMEJ: $p = 0.0169$, +/-1: $p = 0.9999$

E396K-P2 - precise: $p < 0.0299$, -5 MMEJ: $p = 0.7560$, +/-1: $p > 0.9999$

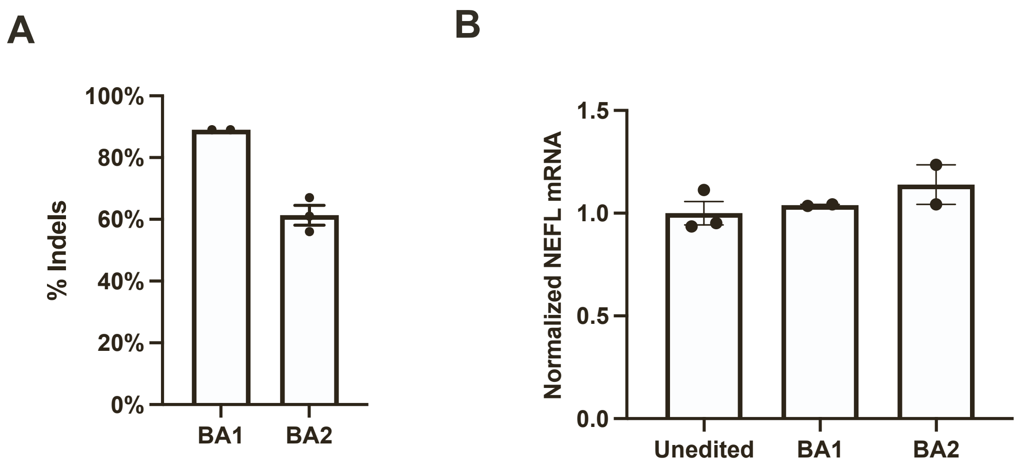

Figure S15: Evaluation of indels produced by biallelic gRNAs targeting *NEFL* intron 1. **(A)** WTC iPSCs were nucleofected with BA1 or BA2 RNP followed by quantification of indels by Sanger sequencing and ICE analysis. Bar graphs represent mean +/- S.E.M. with individual data points representing values from independent transfections (n = 2 – 3). **(B)** Total *NEFL* expression in day 7 i^{3}LMN differentiated from the edited populations shown in (A), as measured by quantitative RT-ddPCR and normalized to *GAPDH*. Bar graphs represent mean +/- S.E.M with individual data points representing independent wells of i^{3}LMNs (n = 2 – 3).

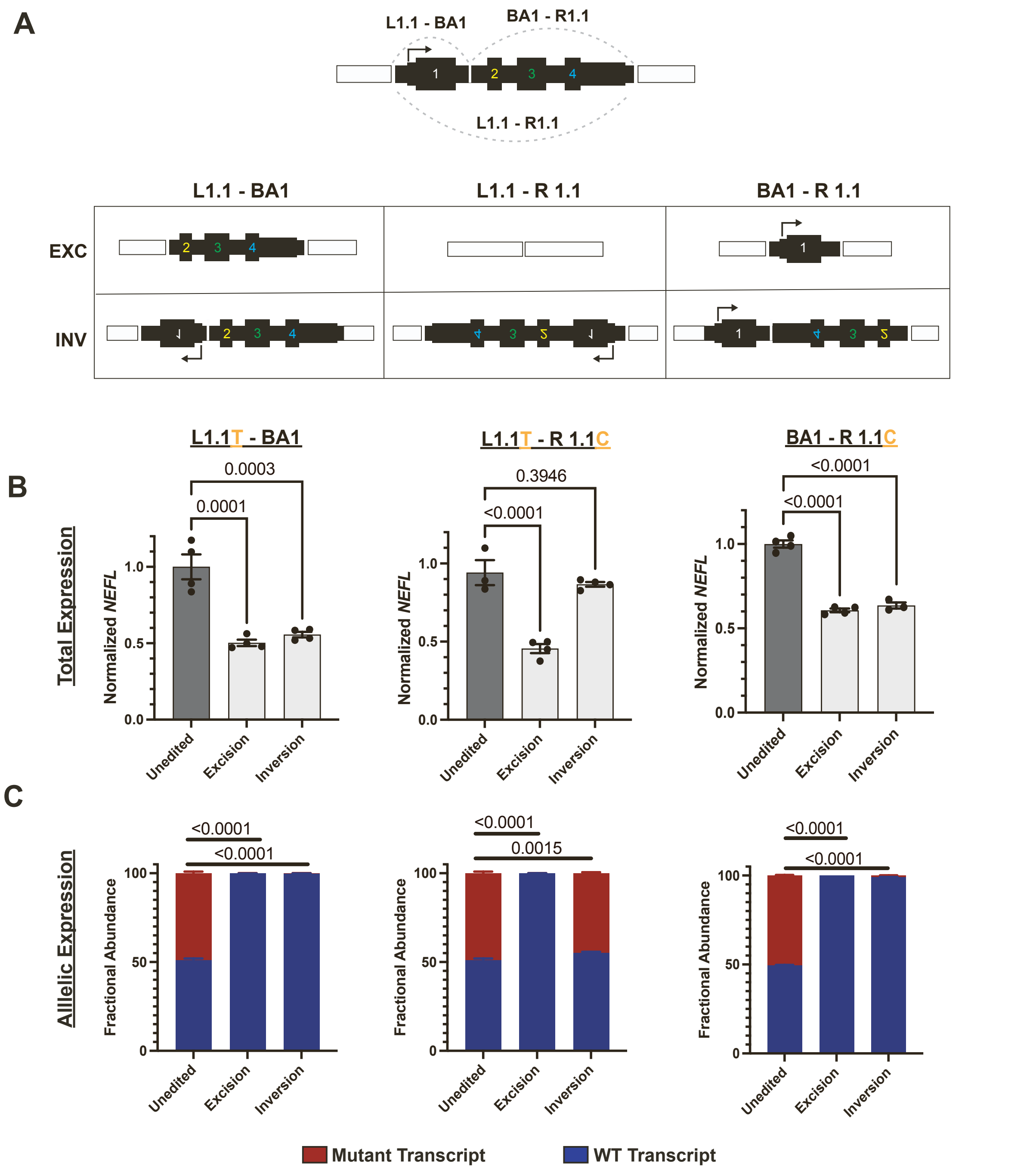

Figure S16: Clonal analysis of editing outcomes reveals variable contributions of excision and inversion to gene inactivation. (A) Schematic of predicted editing outcomes with various combinations of L1.1, R1.1 and intronic biallelic (BA1) gRNAs. E396K-P2 was transfected with each RNP pair and clonal iPSC lines were isolated with each predicted outcome and differentiated into i^{3}LMNs. RNA was isolated on day 7 and measured by

quantitative RT-ddPCR. **(B)** Total *NEFL* expression relative to *GAPDH* and normalized to unedited control. **(C)** Relative allelic expression via allele discrimination ddPCR using a heterozygous SNP in the 3' UTR (rs2976439). Bar graphs represent mean +/- S.E.M. with each data point representing an independent well of i^3LMNs (n = 4). Multiple comparisons were performed by one-way ANOVA followed by Dunnett's post-test.

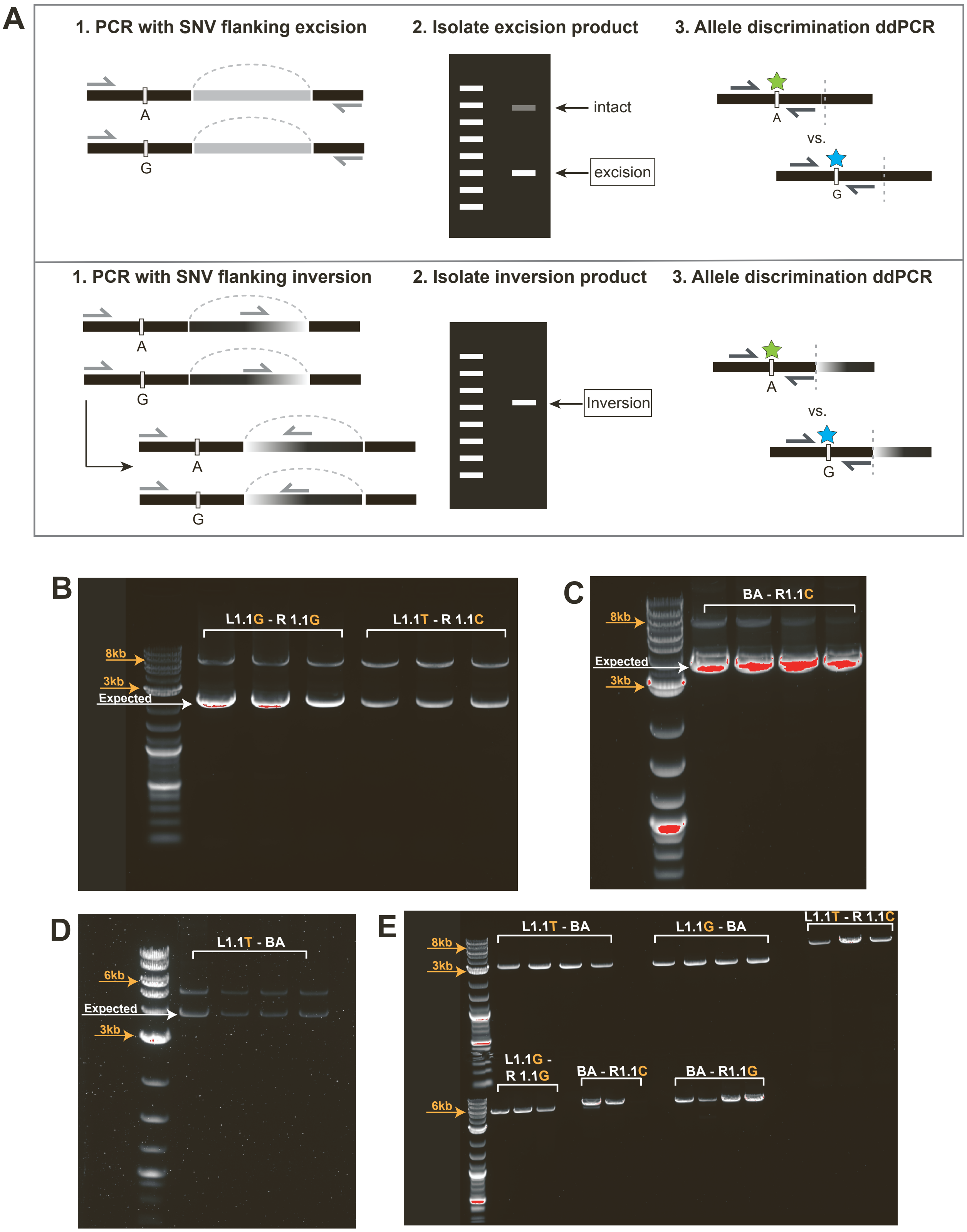
A
1. PCR with SNV flanking excision
A
G
2. Isolate excision product
intact
excision
3. Allele discrimination ddPCR
A
vs.
G
1. PCR with SNV flanking inversion
A
G
A
G
2. Isolate inversion product
Inversion
3. Allele discrimination ddPCR
A
vs.
G
B
L1.1G - R 1.1G
L1.1T - R 1.1C
8kb
3kb
Expected
C
BA - R1.1C
8kb
Expected
3kb
D
L1.1T - BA
6kb
Expected
3kb
E
L1.1T - BA
L1.1G - BA
L1.1T - R 1.1C
8kb
3kb
L1.1G -
R 1.1G
BA - R1.1C
BA - R1.1G
6kb

Figure S17: Multi-step PCR/ddPCR assay to measure allele-specificity of excision and inversion. (A) Assay workflow - 1: Schematic of PCR designed to amplify excised (top) or inverted (bottom) alleles with flanking heterozygous variant. 2: Gel purification to isolate excision or inversion products. 3: Schematic of allele discrimination ddPCR used to quantify specificity. **(B-D)** Examples of gel electrophoresis of PCR products used for excision specificity. **(E)** Examples of gel electrophoresis of PCR products used for inversion specificity.

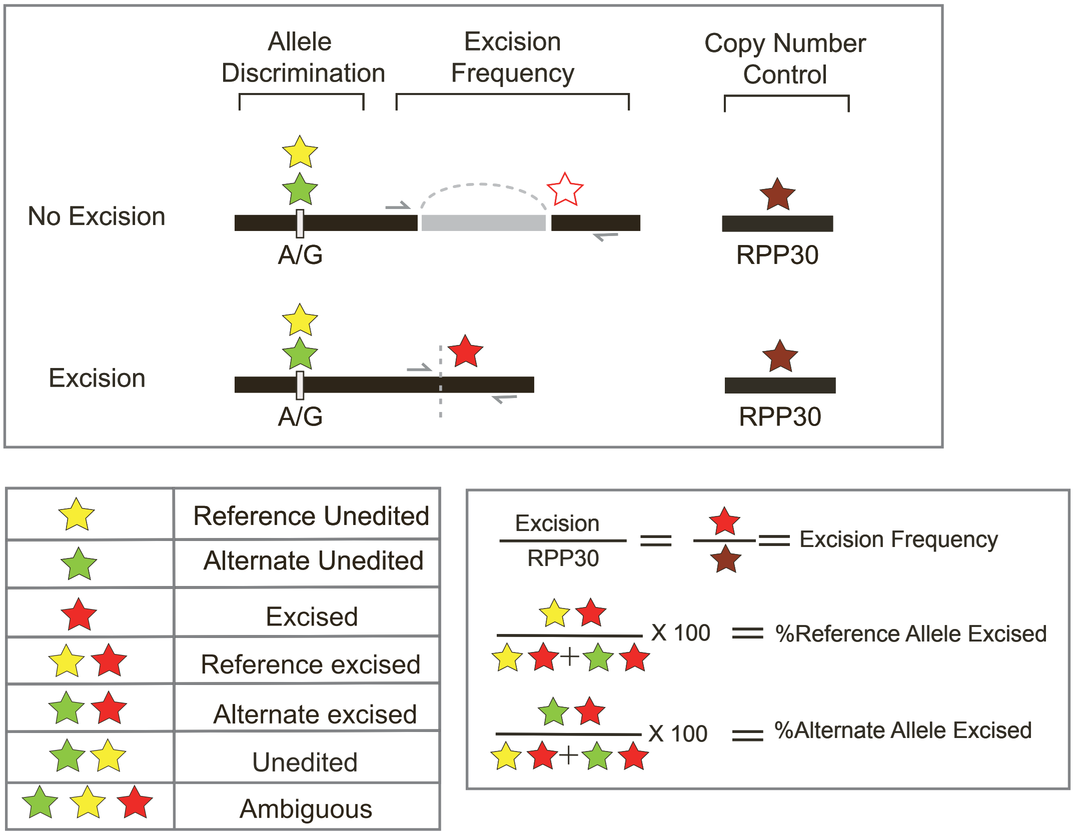

Figure S18: Single-step assay to measure excision frequency and specificity via multiplexed four-color digital PCR. Top: Schematic of multiplexed assay design with four probes (Green = FAM, Yellow = Hex, Red = ROX, Maroon = Cy5). **Bottom-left:** Partition occupancy observed for excision specificity measurements. **Bottom-right**: Calculation used to quantify excision specificity and frequency.

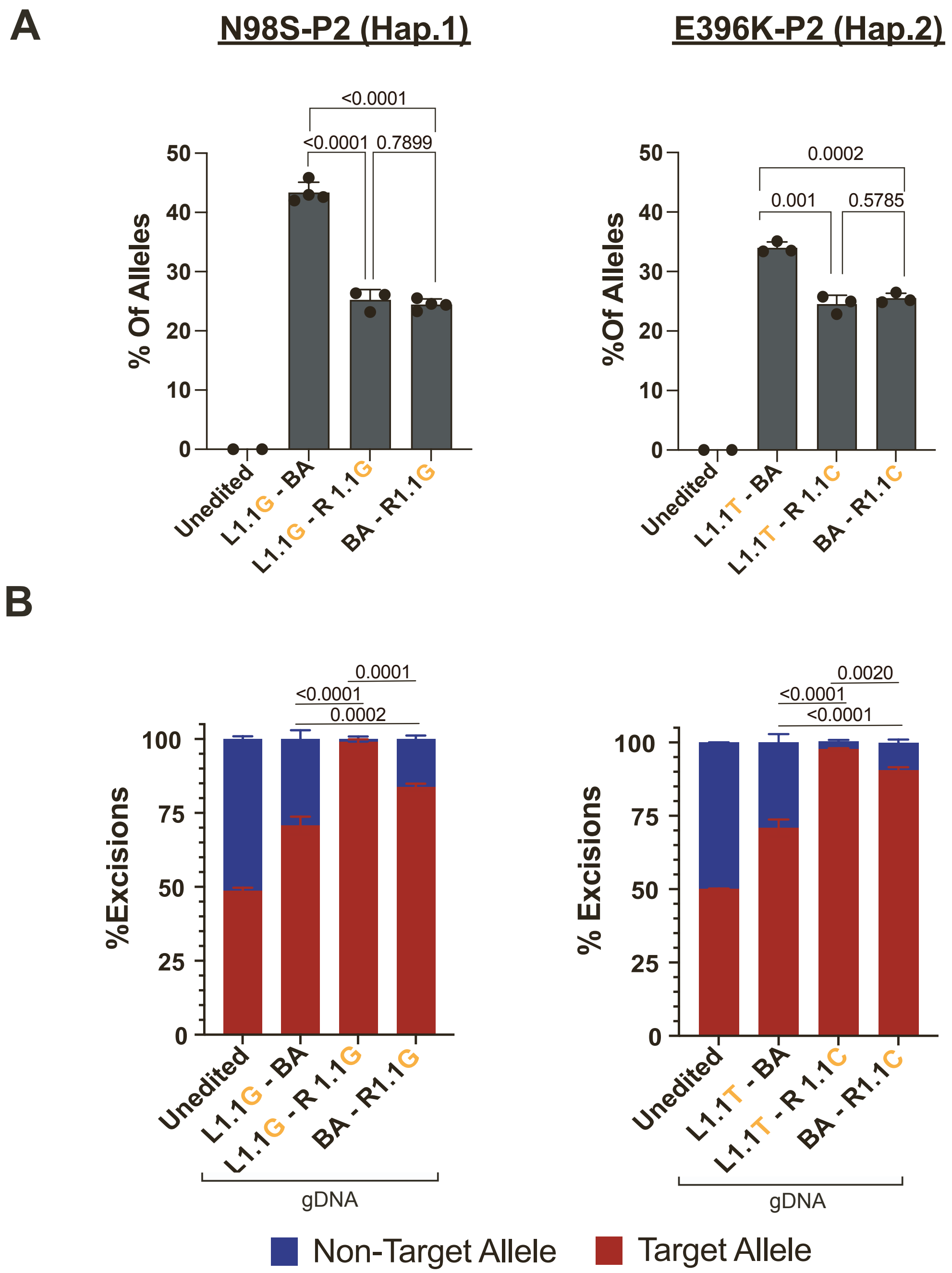

Figure S19: Quantification of excision frequency and specificity via multiplexed four-color digital PCR. The same samples from Figure 5 were assayed using the alternate method described in Figure S18. **(A)** Excision frequency as measured by the ratio of excision (ROX+) signal over RRP30 (Cy5+) signal. **(B)** Excision specificity measured via dPCR allele-discrimination assay for a heterozygous SNP (rs2979685, ref = HEX, alt = FAM) located 5' of

NEFL. Unedited control represents the fractional abundance of HEX vs. FAM in ROX-negative partitions to demonstrate equal abundance of the two alleles at baseline, while edited samples represent the fractional abundance of HEX vs. FAM in ROX-positive partitions that contain an excision event. Bar graphs represent mean +/- S.E.M. with individual data points representing independent transfections (n = 3 – 4). Multiple comparisons were performed by one-way ANOVA followed by Tukey's post-test.

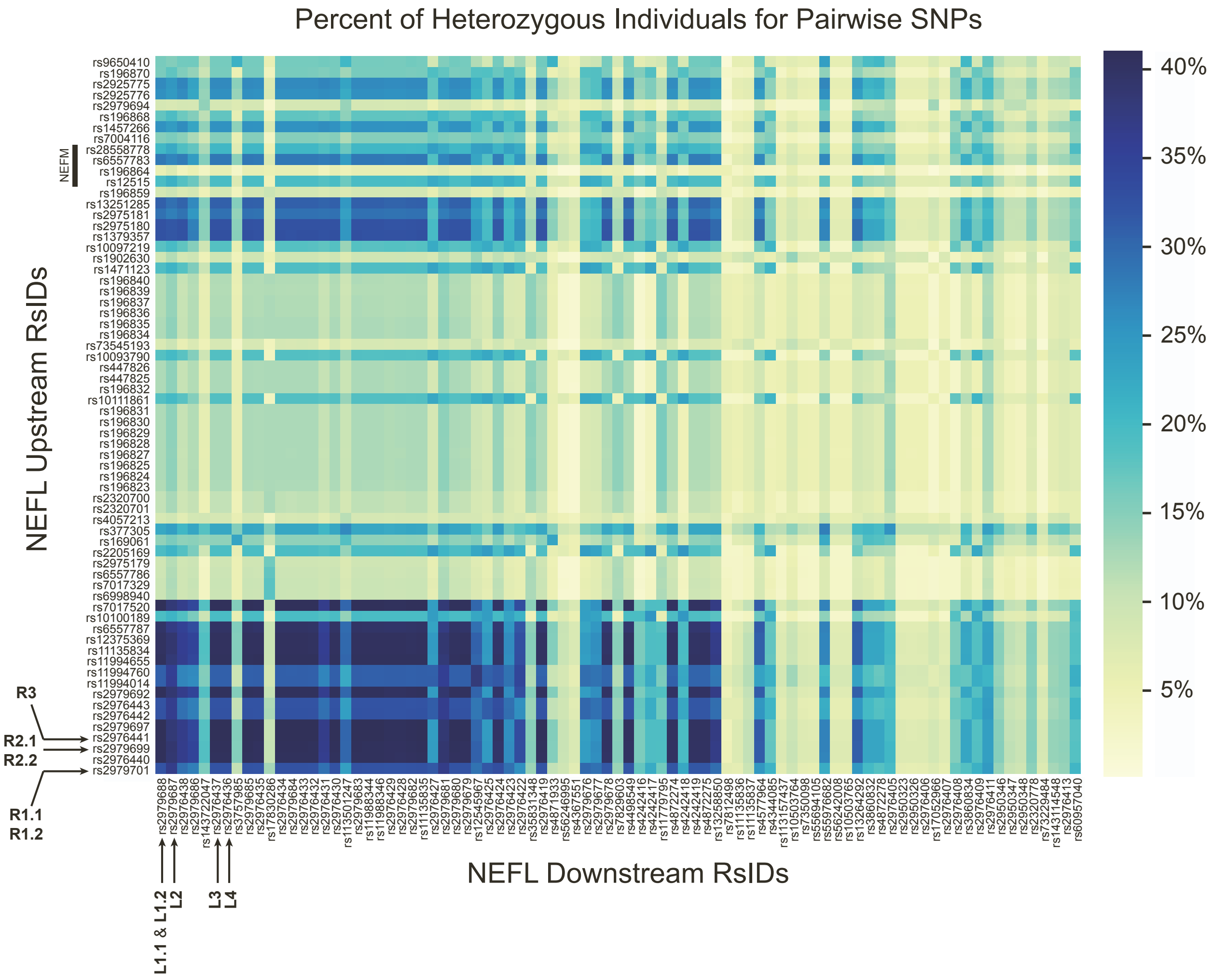

Figure S20: Percentage of individuals heterozygous for SNP pairs flanking *NEFL*. Variant data from 1000 Genomes phase 3, starting with minor allele frequency 0.1-0.9 in global population and within 50 kb of the *NEFL* gene**.** Horizontal axis indicates SNPs downstream (3') of *NEFL* increasing in linear distance from left to right. Vertical axis indicates SNPs upstream (5') of *NEFL* increasing in linear distance from bottom to top. The location of *NEFM* (upstream of *NEFL*) is annotated with a line along the vertical axis. Darker blue colors indicate a higher percentage of heterozygous individuals. Arrows indicate the SNPs targeted by gRNAs in this study. Note that *NEFL* is transcribed right to left from the bottom strand in the reference genome, opposite from our gRNA naming convention.

Table S1: Genomic off-target analysis

gRNA	#	chr	start (hg38)	End (hg38)	Strand	CFD score	Description	Results: N98S-P2 exc	Results: N98S-P2 inv
R1.1G	1	10	78632335	78632357	-	0.49777778	exon:KCNMA1	No mutations	No mutations
R1.1G	2	3	40733428	40733450	+	0.3799606	intergenic:RP11-528N21.1-HMGN2P24	No mutations	No mutations
R1.1G	3	5	8208980	8209002	+	0.34821429	intergenic:RP11-215I16.1-RP11-480D4.1	No mutations	No mutations
R1.1G	4	6	18441294	18441316	+	0.31533434	intron:RNF144B	No mutations	No mutations
R1.1G	5	8	48092200	48092222	+	0.26848498	intergenic:NDUFA5P12-RP11-1134I14.4	No mutations	No mutations
R1.1G	6	11	79198180	79198202	-	0.38787879	intergenic:TENM4-RP11-258O13.1	No mutations	No mutations
R1.1G	7	12	34271299	34271321	+	0.38956522	intergenic:RP11-847H18.3-RP11-313F23.3	Cell line has a 5 bp variant. No other mutations detected.	Cell line has a 5 bp variant. No other mutations detected.
R1.1G	8	4	72155681	72155703	+	0.38181818	intergenic:SLC4A4-RP11-1J11.1	No mutations	No mutations
R1.1G	9	5	133553321	133553343	+	0.36363636	intergenic:CTD-2410N18.4/CDKL3/PPP2CA-PPP2CA	No mutations	No mutations
R1.1G	10	6	68788813	68788835	-	0.32223776	intergenic:RP11-542F9.1-RP11-406O16.1	No mutations	No mutations
R1.1G	11	4	189766672	189766694	-	0.30198176	intergenic:RP11-756P10.2-RP11-818C3.1	No mutations	No mutations
**gRNA**	**#**	**chr**	**start (hg38)**	**End (hg38)**	**Strand**	**CFD score**	**Description**	**Results: E396K-P2 exc**	**Results: E396K-P2 inv**
L1.1T	1	5	2501128	2501150	+	0.4982699	intergenic:Y_RNA-RP11-129I19.2	No mutations	No mutations
L1.1T	2	11	43929675	43929697	-	0.29658922	intergenic:ALKBH3-ALKBH3-AS1	No mutations	No mutations
L1.1T	3	13	102135513	102135535	+	0.27731092	intron:ITGBL1	No mutations	No mutations
R1.1C	4	12	57160521	57160543	+	0.46928467	intron:HSD17B6	No mutations	No mutations
R1.1C	5	10	92818471	92818493	-	0.45389474	intergenic:DDX18P6-LINC00502	No mutations	No mutations
R1.1C	6	3	156692992	156693014	-	0.35	intergenic:LEKR1-KLF3P2	No mutations	No mutations
R1.1C	7	3	80744740	80744762	-	0.27575758	intergenic:RP11-481N16.1-RP11-47P18.1	PCR failed	PCR failed
R1.1C	8	3	4047631	4047653	+	0.44426162	intergenic:PNPT1P1-SUMF1	No mutations	No mutations

R1.1C	9	2	57033442	57033464	+	0.56384439	intergenic:AC008173.1-snoU13	Homozygous alternate SNP. No other mutations detected.	Homozygous alternate SNP. No other mutations detected.
R1.1C	10	5	67112973	67112995	-	0.51948052	intergenic:RP11-434D9.1-RP11-83M16.6	No mutations	No mutations
R1.1C	11	2	56267847	56267869	+	0.39751553	intron:AC011306.2	No mutations	No mutations
R1.1C	12	4	1103514	1103536	-	0.37151703	intron:RNF212	No mutations	No mutations
R1.1C	13	1	173767314	173767336	+	0.34208145	intergenic:RN7SKP160-CENPL	No mutations	No mutations
R1.1C	14	1	159346828	159346850	-	0.32814779	intergenic:OR10J8P-OR10J9P	No mutations	No mutations
R1.1C	15	4	99726082	99726104	-	0.26526316	intergenic:BTF3P13-EIF4E	No mutations	No mutations
R1.1C	16	10	54100207	54100229	+	0.26352941	intergenic:DKK1-RP11-346D6.4	No mutations	No mutations
**gRNA**	**#**	**chr**	**start (hg38)**	**End (hg38)**	**Strand**	**CFD score**	**Description**	**Results: N98S-P2 FS**	**Results: N98S-P2 Cor**
N98S	1	1	148316436	148316458	+	0.7143	intergenic: RP11-495P10.8/RP11-495P10.5/RP11-495P10.7-RP11-495P10.3	Sequencing Failed	Sequencing Failed
N98S	2	12	5752303	5752303	-	0.4412	intron: ANO2	PCR failed	PCR failed
N98S	3	10	75661072	75661094	-	0.3956	intergenic: RP11-310J24.3-RP11-367B6.2	No mutations	No mutations
N98S	4	20	48137627	48137649	+	0.3652	intergenic: AL139351.1-LINC00494	No mutations	No mutations
N98S	5	18	39020019	39020041	-	0.3649	intergenic: RN7SKP182-RNU6-706P	No mutations	No mutations
N98S	6	4	3605528	3605550	-	0.3563	intergenic: LINC00955-RP3-368B9.2	No mutations	No mutations
N98S	7	9	65426450	65426472	+	0.3563	intergenic: FOXD4L5-Y_RNA	No mutations	No mutations
N98S	8	8	1925519	1925541	-	0.3545	intron: ARHGEF10	No mutations	No mutations
N98S	9	13	111494882	111494904	+	0.3254	intergenic: TEX29-RP11-65D24.2	No mutations	No mutations
N98S	10	8	120892179	120892201	-	0.3196	intergenic: RP11-713M15.2/SNTB1-RP11-369K17.1	No mutations	No mutations
N98S	11	9	14223924	14223946	+	0.279	intron: NFIB	No mutations	No mutations

Table S2: ssODN sequences for excisions

**L1.1G**	**BA**	CTTGGCTGCAGCAGCGCGCTGCCCCCACTGTTCCGCATTAAAGCTGCCCA GCCCTTGTTG
**L1.1G**	**R1.1G**	CCTTGGCTGCAGCAGCGCGCTGCCCCCACTCACTAACCTATCACAGAGTTA TAGTGAGAA
**BA**	**R1.1G**	GGGAGTGTGCTCCGTGCTGCTGCACCGGCGCACTAACCTATCACAGAGTTA TAGTGAGAA
**L1.1T**	**BA**	CTTGGCTGCAGCAGCGCGCTGCCCCCACTGTTCCGCATTAAAGCTGCCCAG CCCTTGTTG
**L1.1T**	**R1.1C**	CCTTGGCTGCAGCAGCGCGCTGCCCCCACTGACTAACCTATCACAGAGTTAT AGTGAGAA
**BA**	**R1.1C**	GGGAGTGTGCTCCGTGCTGCTGCACCGGCGGACTAACCTATCACAGAGTTAT AGTGAGAA

Table S3: Antibodies

**Name/Antigen**	**Fluorophore**	**Vendor**	**Catalog / Clone #**	**Dilution / *μL per 1E6 cells**
NfL	N/A	Millipore	AB9568	1:1000 for CX7, 1:500 for Keyence
Beta3-Tubulin	N/A	ThermoFisher	480011	1:250
HB9	N/A	DSHB	81.5c10	1:200
Goat anti-rabbit	AF488	Invitrogen	A11034	1:500
Goat anti-mouse	AF594	Invitrogen	A11032	1:500
Live/Dead	NIR	Thermofisher	L34957A	1:2500
Tra-1-60	BV421	BD	562711	0.625*
SSEA-3	FITC	BD	560236	5*
Tra-1-81	PE	BD	560161	5*
SSEA-4	PE-Cy7	Biolegend	330420	0.625*
SSEA-1	APC-Cy7	Biolegend	323048	0.625*
Nanog	AF647	Biolegend	674210	2*
Oct ¾	BV421	BD	560306	5*
Sox2	AF488	BD	245610	5*

Table S4: CLYBL hNIL genotyping junction PCR primer sequences

**Junction PCR Primers for hNIL CLYBL integration and Post-CRE Junction PCR Primers**	
hNIL CLYBL 5' Junction F	CAGACAAGTCAGTAGGGCCA
hNIL CLYBL 5' Junction R	AGAAGACTTCCTCTGCCCTC

hNIL CLYBL 3' Junction F	CACCAGCAACCTGACGTTTT
hNIL CLYBL 3' Junction R	TTTTATAGGCGCCCACCGTA
CLYBL WT F	TGACTAAACACTGTGCCCCA
CLYBL WT R	AGGCAGGATGAATTGGTGGA
CF28	CCTCAGCCCAGTTTCCACTTG
CF29	GGCTATGAACTAATGACCCCGT

Table S5: hNIL copy number custom 20x ddPCR primer/ probe mixes

**Name**	**F Primer**	**R Primer**	**Probe**
Neomycin Assay	CATGGCTGATGCAATGCG	TCGCTTGGTGGTCGAATG	CGCTTGATCCGGCTACCTGCC
TRE3G Assay	TACGGTGGGCGCCTATAA	AGTGGTACGGAAAGTTGGTATAAG	AGATCGCCTGGAGCAATTCCACAA

Table S6: Sequences of gRNA genomic targets

**sgRNA Name**	**Target**	**Sequence**	**Allele Specific (AS) or Biallelic (BA)**
N98S	N98S	AGCTGGCGAAGCGGTCACTG	AS
E396K	E396K	CAGGAAACTCTTGGAAGGCA	AS
L1.1G	rs2979688	GATCACGGCACGCCGGCCAG	AS
L1.2G	rs2979688	AGCGCGCTGCCCCCACTGGC	AS
L2T	rs2979687	TCACGGGGTCTGGGCAATGC	AS
L3C	rs2976437	TCTACATATGGGTAATTGGG	AS
L4A	rs2976436	ACCCATATGTAGATGAAGCA	AS
R1.1G	rs2979701	TCTGTGATAGGTTAGTGTAG	AS
R1.2G	rs2979701	CTGTGATAGGTTAGTGTAGA	AS
R2.1T	rs2979699	TACCAGGGTGACTGGAGTGC	AS
R2.2T	rs2979699	TTCCAGCACTCCAGTCACCC	AS
R3G	rs2976441	GCTTAAATGTCATTCTCTAA	AS
L1.1T	rs2979688	GATCACGGCACGCCGTCCAG	AS

L1.2T	rs2979688	AGCGCGCTGCCCCCACTGGA	AS
R1.1C	rs2979701	TCTGTGATAGGTTAGTCTAG	AS
R1.2C	rs2979701	CTGTGATAGGTTAGTCTAGA	AS
BA1	Intron 1	GCAGCTTTAATGCGGAACGC	BA
BA2	Intron 1	CCTTTATTTAGTAGGTAGAC	BA
NT	Non-Targeting	GGCCAAACGTGCCCTGACGG	NT

Table S7: Digital PCR assays for excision and inversion frequency

F primer	R primer	Probe	Excision Assay
HW_ddXR_79_F	HW_ddXR_63_R	HW79	L3-R1.1, L3-R1.2, L4-R1.1, L4-R1.2
HW_ddXR_79_F	HW_ddXR_55_R	HW79	L3-R2.1, L3-R2.2, L4-R2.1, L4-R2.2
HW_ddXR_79_F	HW_ddXR_53_R	HW79	L3-R3, L4-R3
HW_ddXR_74_F	HW_ddXR_63_R	HW63	L2-R1.1
HW_ddXR_72_F	HW_ddXR_63_R	HW63	L1.1-R1.1, L1.2-R1.1
CM_ddXR_4790_F	CM_ddXR_11390_R	HW69	L1.1-R3, L1.2-R3
CM_ddXR_4590_F	CM_ddXR_11390_R	HW74	L2-R3
CM_ddXR_4790_F	CM_ddXR_11270_R	HW69	L1.1-R2.1, L1.1-R2.2, L1.2-R2.1, L1.2-R2.2
CM_ddXR_4590_F	CM_ddXR_11270_R	HW74	L2-R2.1, L2-R2.2
CM_ddXR_4890_F	CM_ddXR_10970_R	HW63	L1.1-R1.2, L1.2-R1.2
CM_ddXR_4650_F	CM_ddXR_10970_R	HW63	L2-R1.2
PD_ddXR_72/73F_anchored	PD_ddXR_HW15_R	PD_ddXR_72/63_probe	L1.1 - BA
HW_ddXR_15/63F	HW_ddXR_63_R	HW63	BA - R1.1
NEFL_EX4common_exc_F	NEFL_EX4common_exc_R	NEFLexon4	*NEFL* Loss of Signal

F primer	R primer	Probe	Inversion Assay
HW_ddXR_79_F	CM_iddXR_63_R	HW79	L3-R1.1, L3-R1.2, L4-R1.1, L4-R1.2
HW_ddXR_79_F	CM_iddXR_55_R	HW79	L3-R2.1, L3-R2.2, L4-R2.1, L4-R2.2
HW_ddXR_79_F	CM_iddXR_53_R	HW79	L3-R3, L4-R3
CM_iddXR_74_F	HW_ddXR_63_R	HW63	L2-R1.1
CM_NEFL_4996_R	HW_ddXR_63_R	HW63	L1.1-R1.1, L1.2-R1.1
CM_iddXR_11250_F	CM_iddXR_11430_R	HW13	L1.1-R3, L1.2-R3
CM_ddXR_4590_F	CM_iddXR_4840_R	HW74	L2-R3
CM_iddXR_11110_F	CM_iddXR_11310_R	HW13	L1.1-R2.1, L1.1-R2.2, L1.2-R2.1, L1.2-R2.2
CM_ddXR_4590_F	CM_iddXR_4800_R	HW74	L2-R2.1, L2-R2.2
CM_iddXR_10800_F	CM_iddXR_10950_R	HW63	L1.1-R1.2, L1.2-R1.2
CM_iddXR_10810_F	CM_iddXR_10950_R	HW63	L2-R1.2
PD_ddXR_72/73F_anchored	PD_iddXR_HW15_R	PD_ddXR_72/63_probe	L1.1 - BA
HW_iddXR_15/63F	HW_ddXR_63_R	HW63	BA - R1.1

Table S8: Specificity assays for inversions and excisions

Guide Pair	F Primer	R Primer	Allele discrimination assay	Assay Type
L1.1 - BA	NEFL_forASaround rs2979685	PD_7984_R	rs2979685	Excision Specificity
L1.1 - R1.1	NEFL_forASaround rs2979685	HW_ddXR_63_R	rs2979685	
BA - R1.1	NEFL_forASaround rs2979685	HW_ddXR_63_R	rs2979685	
L1.1 - BA	NEFL_forASaround rs2979685	HW_NEFL_5740_F	rs2979685	Inversion Specificity

L1.1 - R1.1	NEFL_forASaround rs2979685	BMJS_NEFL_7758F	rs2979685	
BA - R1.1	NEFL_forASaround rs2979685	HW_ddXR_63_R	rs2979685	

Table S9: Primer and probe sequences for excision and inversion frequency / specificity assays

**CATEGORY**	**NAME**	**SEQUENCE (5' → 3')**
Primer	CM_ddXR_4790_F	GGCATGGGATCTCAGAGAAA
Primer	CM_ddXR_11390_R	CAAAGAATTTGACCCACTAGAAGAG
Primer	CM_ddXR_4590_F	GAGGTTCCTGGTGGGAAA
Primer	CM_ddXR_11270_R	AAAGATGAGTGCTCCAGAAA
Primer	CM_ddXR_4890_F	CGCAGAATCCTCGCCTT
Primer	CM_ddXR_10970_R	CCCTGGGAGAAGGGTTAGA
Primer	CM_ddXR_4650_F	GAGGTGACGGGATACAGAAA
Primer	HW_ddXR_79_F	TATGCAGACTCACACACACTG
Primer	HW_ddXR_72_F	GCAGAATCCTCGCCTTGG
Primer	HW_ddXR_74_F	GGGCAACTTAAGGATCCAAGT
Primer	HW_ddXR_63_R	GTGGTGGCAGTATAAATTGAAAGA
Primer	HW_ddXR_55_R	ATCCTGTGACAGATGGGAGAA
Primer	HW_ddXR_53_R	TTCAAAGAATTTGACCCACTAGAAG
Primer	CM_iddXR_74_F	GCTCAGAGGGCCCTGATTTT
Primer	CM_iddXR_63_R	CTGTGGTCAGTGCCCCTTTT
Primer	CM_iddXR_55_R	CTGCCTAGTGCTGACTCCTG
Primer	CM_iddXR_53_R	CTCCACTTCCAGCACTCCAG
Primer	CM_NEFL_4996_R	CCGTTCTGCCACCCCTATTT
Primer	CM_iddXR_11250_F	GAGGATGGATGGCTGTGTG
Primer	CM_iddXR_11110_F	GAGGATGGATGGCTGTGTG
Primer	CM_iddXR_10800_F	TATTTATACGCCGGGAGGCT
Primer	CM_iddXR_10810_F	CCCTCACTCATTTCCCTTCTG
Primer	CM_iddXR_11430_R	AGATGCTAATGGCAAGAATCAA
Primer	CM_iddXR_4840_R	CTCCCATCTGTCACAGGATTT
Primer	CM_iddXR_11310_R	AATCTGAAGGGTCAGTAGGAAC
Primer	CM_iddXR_4800_R	CTGACTCCTGCCTAGTTCTCTA
Primer	CM_iddXR_10950_R	GTGGTGGCAGTATAAATTGAAAGAT
Primer	NEFL_EX4common_exc_F	GCATGGACCACGCTTATGA
Primer	NEFL_EX4common_exc_R	CACCGAAGGTTCAAAGGACTAT
Primer	PD_ddXR_72/73F_anchored	CCCTCTGAGCAAAGTGGAAA

Primer	HW_ddXR_15/63F	TGTAGTCTGGGAGTGTGCT
Primer	HW_iddXR_15/63F	CCCAATTCCCACGTCTTCC
Primer	NEFL_forASaroundrs2979685	GGCTGTCGTGGAGTATGAGG
Primer	PD_ddXR_HW15_R	CAGGTCAGTAGAGAGCTGAT
Primer	HW_ddXR_63_R	GTGGTGGCAGTATAAATTGAAAGA
Primer	PD_iddXR_HW15_R	TGTAGTCTGGGAGTGTGCT
Primer	PD_7984_R	CTCCTCTTGGACATGGCTGG
Primer	HW_ddXR_72_F	GCAGAATCCTCGCCTTGG
Primer	CM_NEFL_4996_R	CCGTTCTGCCACCCCTATTT
Primer	HW_NEFL_5740F	TCGACAGCTTGATGGACGAA
Primer	BMJS_NEFL_7758F	TCCTGCTTGCCTTTGTGTTTAG
Probe	HW79	TGAGGTTTGCAGGGAGCAGGTTAA
Probe	HW63	ACACCTCCATGTCTTAGATCCTTCCACA
Probe	HW13	CAGGCTGCGTCAGG
Probe	HW69	TCTGAGCAAAGTGGAAAGGACGACC
Probe	HW74	CTGCGAGGTGACGGGATACAGAAA
Probe	PD_ddXR_72/63_probe	CTGCGAGGTGACGGGATACAGAAA
Probe	NEFLexon4	ACGGCAATGTGAATCA

Table S10: Primer sequences for next generation sequencing and sanger sequencing

**Name**	**Sequence 5' → 3'**
BMJS_NGS_10733F	TCCCTACACGACGCTCTTCCGATCTcagctAgttctctttcagtattc cctctcc
BMJS_NGS_10965R	GTTCAGACGTGTGCTCTTCCGATCTcagctAggttagagtggtggc agtataaa
BMJS_NGS_4810F	TCCCTACACGACGCTCTTCCGATCTcagctAccctctgagcaaagt ggaaa
BMJS_NGS_5025R	GTTCAGACGTGTGCTCTTCCGATCTcagctAgaggatggatggct gtgtg
QC65_NGS-Fwd_NF-L1	CTTTCCCTACACGACGCTCTTCCGATCTNNNNaaagtggaaa ggacgaccgccg
QC70_NGS-Rev_NF-R1	GGAGTTCAGACGTGTGCTCTTCCGATCTggagaccaagagtag gccctggg

HW_NEFL_4388F	TCCCTATGTGTTAAGCAGCTC
HW_NEFL_5932R	GCGGCTCTTGAACCATTCCT
HW_NEFL_10113F	ACACGCCCCATGTATCCTTT
HW_ddXR_63R	GTGGTGGCAGTATAAATTGAAAGA
