# Supplementary material for: Haplotype editing with CRISPR-Cas9 as a therapeutic approach for dominant-negative missense mutations in NEFL
Source: Mol Ther. 2025 Nov 19;34(3):1633–51. doi: 10.1016/j.ymthe.2025.11.026 (PMC12974165; doi:10.1016/j.ymthe.2025.11.026)
Supplement: Document S2. Article plus supplemental information [file mmc2.pdf]

Molecular Therapy
Original Article

Haplotype editing with CRISPR-Cas9 as a therapeutic approach for dominant-negative missense mutations in *NEFL*

Poorvi H. Dua,[1,2] Bazilco M.J. Simon,[2] Chiara B.E. Marley,[1,2] Carissa M. Feliciano,[1,2] Hannah L. Watry,[2] Quinn T. Cowan,[2] Dylan Steury,[2,3] Abin Abraham,[4,5,6] Erin N. Gilbertson,[7,8,9] Grace D. Ramey,[7,8] John A. Capra,[8,10,11] Bruce R. Conklin,[2,12,13,14] and Luke M. Judge[1,2]

[1]Department of Pediatrics, University of California, San Francisco, San Francisco, CA 94143, USA; [2]Gladstone Institutes, San Francisco, CA 94158, USA; [3]University of California, Berkeley, Berkeley, CA 94720, USA; [4]Vanderbilt Genetics Institute, Vanderbilt University, Nashville, TN 37232, USA; [5]Vanderbilt University Medical Center, Vanderbilt University, Nashville, TN 37232, USA; [6]Division of Neonatology, Children's Hospital of Philadelphia, Philadelphia, PA 19104, USA; [7]Biomedical Informatics Graduate Program, University of California, San Francisco, San Francisco, CA 94143, USA; [8]Bakar Computational Health Sciences Institute, University of California, San Francisco, San Francisco, CA 94143, USA; [9]Department of Genetics, Yale School of Medicine, New Haven, CT 06520, USA; [10]Department of Epidemiology and Biostatistics, University of California, San Francisco, San Francisco, CA 94143, USA; [11]Department of Bioengineering and Therapeutic Sciences, University of California, San Francisco, San Francisco, CA 94143, USA; [12]Department of Ophthalmology, University of California, San Francisco, San Francisco, CA 94143, USA; [13]Department of Medicine, University of California, San Francisco, San Francisco, CA 94143, USA; [14]Innovative Genomics Institute, Berkeley, CA 94720, USA

Inactivation of disease alleles by allele-specific editing is a promising approach to treat dominant-negative genetic disorders, provided the causative gene is haplosufficient. We previously edited a dominant *NEFL* missense mutation causing Charcot-Marie-Tooth type 2E (CMT2E) with inactivating frameshifts and rescued disease-relevant phenotypes in induced pluripotent stem cell (iPSC)-derived motor neurons. However, a multitude of different *NEFL* missense mutations cause CMT2E. Here, we addressed this challenge by targeting common single-nucleotide polymorphisms in *cis* with *NEFL* disease mutations for gene excision. We validated this haplotype editing approach in two iPSC lines with different missense mutations and demonstrated phenotypic rescue in iPSC-motor neurons. Surprisingly, our analysis revealed that gene inversion, a frequent by-product of excision editing, failed to reliably disrupt mutant allele expression. We deployed novel molecular assays to optimize our approach and achieve therapeutic levels of editing in immature iPSC-motor neurons. Finally, population genetics analysis demonstrated the power of haplotype editing to enable therapeutic development for the greatest number of patients. Our data serve as an important case study for many dominant genetic disorders amenable to this approach.

INTRODUCTION

The discovery and advancement of gene-editing technologies are revolutionizing how scientists and clinicians approach treatments for genetic diseases.[1] The CRISPR-Cas system is a powerful gene-editing tool with two fundamental components, a programmable guide RNA (gRNA) that encodes sequences complementary to a genomic locus, and a Cas enzyme that induces a double-stranded break (DSB) in DNA when its associated gRNA binds to the target site.[2] DSB repair pathways can generate short insertions or deletions (indels) at the cut site that disrupt the DNA sequence; this simplest form of gene editing is the basis of the first approved gene-editing therapy for hemoglobinopathies.[3,4] Hemoglobinopathies and other conditions tested in ongoing clinical trials are representative of the small number of disorders where the disruption of both alleles of a coding gene or regulatory element could lead to therapeutic benefit with minimal toxicity.[5–8] However, the majority of genetic disorders will require more complex and precise editing strategies. For example, editing dominant missense mutations requires single-nucleotide specificity to avoid disrupting the normal allele and further impairing cellular function. We and other groups have demonstrated that allele-specific editing to selectively disrupt a mutant allele is a promising strategy for dominant-negative or gain-of-function disorders where a single allele is adequate for normal function.[9–12]

An example of an autosomal-dominant disorder amenable to allele-specific editing is Charcot-Marie-Tooth disease type 2E (CMT2E), a genetic neuropathy with no current treatment. CMT2E is an axonal neuropathy that leads to peripheral nerve degeneration with motor and sensory impairment that can be severe, often manifesting with progressive weakness starting in childhood.[13] Clinical and

Received 10 February 2025; accepted 14 November 2025;
https://doi.org/10.1016/j.ymthe.2025.11.026.

Correspondence: Bruce R. Conklin, MD, Department of Medicine, University of California, San Francisco, San Francisco, CA 94143, USA.
E-mail: bconklin@gladstone.ucsf.edu
Correspondence: Luke M. Judge, MD PhD, Gladstone Institutes, San Francisco, CA 94158, USA.
E-mail: luke.judge@ucsf.edu

© 2025 The Authors. Published by Elsevier Inc. on behalf of The American Society of Gene and Cell Therapy.
This is an open access article under the CC BY license (http://creativecommons.org/licenses/by/4.0/).

pathological findings in patients include reduced nerve conduction velocities and compound muscle action potentials, small axon caliber, and reduced or absent neurofilaments in distal axons,[14] findings that have been recapitulated in mouse models.[15–17] CMT2E is caused by mutations in the *NEFL* gene,[13] which encodes the neurofilament light chain protein (NfL) that forms the core of hetero-oligomeric neurofilaments critical for axonal growth and function.[18] Missense mutations in *NEFL* act via dominant-negative mechanisms whereby mutant NfL interferes with the proper assembly, transport, or function of neurofilament oligomers.[19–22] Interestingly, loss-of-function mutations in *NEFL* are recessive, and people heterozygous for these mutations exhibit normal neurological function, evidence that selective inactivation of dominant missense mutations in *NEFL* should be well tolerated in patients.[23,24] Motor neurons differentiated from patient-derived induced pluripotent stem cells (iPSCs) have been demonstrated to recapitulate features of CMT2E, including abnormal distribution of neurofilament proteins *in vitro*.[25,26] We previously utilized this model to demonstrate rescue of aberrant neurofilament accumulation in cell bodies and release into media after inactivating the *NEFL* N98S mutant allele via selective introduction of frameshift-producing indels at the mutation site.[10] However, patients with CMT2E can present with any one of more than 50 different causative missense mutations distributed over all *NEFL* exons (Figure 1A). This diversity of causative mutations motivated our search for a mutation-agnostic editing strategy that would be therapeutic for a greater proportion of the patient population.

In this study, we achieved mutation-agnostic and allele-specific editing via a dual-gRNA CRISPR system that targets commonly inherited non-coding variants in the human population. CRISPR editing using paired gRNA can efficiently induce large-scale excision or inversion of the intervening sequence.[27–30] Excision-based editing has progressed to clinical trials to revert inherited retinal dystrophy or eliminate HIV proviral genomes, highlighting the therapeutic potential of this concept.[31,32] Furthermore, allele-specific excision has been proposed as a therapeutic strategy for repeat expansion disorders such as frontotemporal dementia-amyotrophic lateral sclerosis and Huntington disease[33–35] and dominant missense mutations causing corneal dystrophy.[36,37] Here, we evaluate allele-specific editing in two unrelated patient-derived iPSC lines with disease mutations linked to the two most common haplotypes in the global population. We utilize novel molecular assays and clonal analysis of editing outcomes to rigorously interrogate and optimize haplotype editing in a robust disease model. Our results provide proof of principle for haplotype editing as a therapeutic strategy for any dominant missense mutation causing CMT2E. While our studies are focused on mutations in *NEFL* and their effects on motor neurons, the approach and discoveries we describe are broadly applicable to a wide variety of dominant genetic disorders.

RESULTS

Mutation-specific gRNAs induce indels with varying efficiency

Our previous study developed a robust iPSC-derived model of genetic motor neuropathy in which we established allele-specific editing as a promising therapeutic strategy for dominant mutations in *NEFL*.[10] That study used a single CMT2E iPSC line with the N98S missense mutation (N98S-P1). To test whether the same approach would generalize to other *NEFL* mutations and genetic backgrounds, we generated CMT2E iPSC lines from an unrelated N98S patient of different sex and ethnic background (N98S-P2), and from two related patients with the E396K missense mutation (E396K-P1 and E396K-P2). Both mutations are classified as definitively pathogenic, validated by functional studies with *in vitro* and *in vivo* models.[10,13–17,22,25] We engineered these patient-derived iPSCs with integrated, inducible, and isogenic (i^3) transcription factors so we could rapidly and efficiently differentiate them into lower motor neurons (i^3LMNs).[38] We observed that E396K-P2 performed qualitatively better during differentiation, with more consistent expression of pluripotency markers compared with E396K-P1 (Figure S1), so we prioritized E396K-P2 for subsequent experiments in i^3LMNs.

Our established allele-specific editing strategy used a single mutation-specific gRNA and high-fidelity Cas9 derived from *Streptococcus pyogenes* (HiFiCas9)[39] that allowed for the introduction of frameshift indels in the coding region of the mutant *NEFL* allele. This, in turn, induced nonsense-mediated decay of the mutant transcript. We isolated clonal iPSC lines with isogenic correction (N98S-P2-cor) as well as frameshift indels at the N98S mutation site (N98S-P2-fs) as described previously (Figures S2A–S2D).[10] When we differentiated the frameshift line into i^3LMNs, we saw that the expression of total *NEFL* mRNA was reduced by approximately 50% compared with unedited N98S-P2, with a near-total extinction of the mutant allele (Figures S2C and S2D). Our group previously developed an automated imaging analysis assay to measure the pathologic NfL accumulation in the cell bodies of N98S mutant motor neurons.[10] Using this assay, we confirmed that the allele-specific frameshift rescued the aberrant accumulation of NfL in motor neuron cell bodies, consistent with our original results in the N98S-P1 patient line (Figure S2E). Additionally, we confirmed that spontaneous action potentials were similar in clonal N98S-P2-fs i^3LMNs compared with unedited N98S-P2 and N98S-P2-corrected i^3LMNs, indicating that gene editing did not disrupt basic neuronal function (Figure S2F).

We attempted to extend this editing strategy to the E396K mutation (Figure S3A). However, compared to the high editing efficiency in both N98S patient lines, we detected fewer than 5% indels at the target locus in both E396K patient lines, as measured by amplicon sequencing (Figures S3B and S3C). We confirmed the nuclease activity of the E396K gRNA-HiFiCas9 ribonucleoprotein (RNP) using an *in vitro* cleavage assay (Figure S3D). Alternate hypotheses to explain the low editing efficiency for targeting E396K include chromatin modifications that could impair Cas9 binding to the target site in cells but are absent in our *in vitro* assay. It is also possible that DSBs at the E396K site may be prone to error-free repair in iPSCs. Considering that patients with CMT2E have been reported with any one of >50 different missense mutations in *NEFL* (Figure 1A)

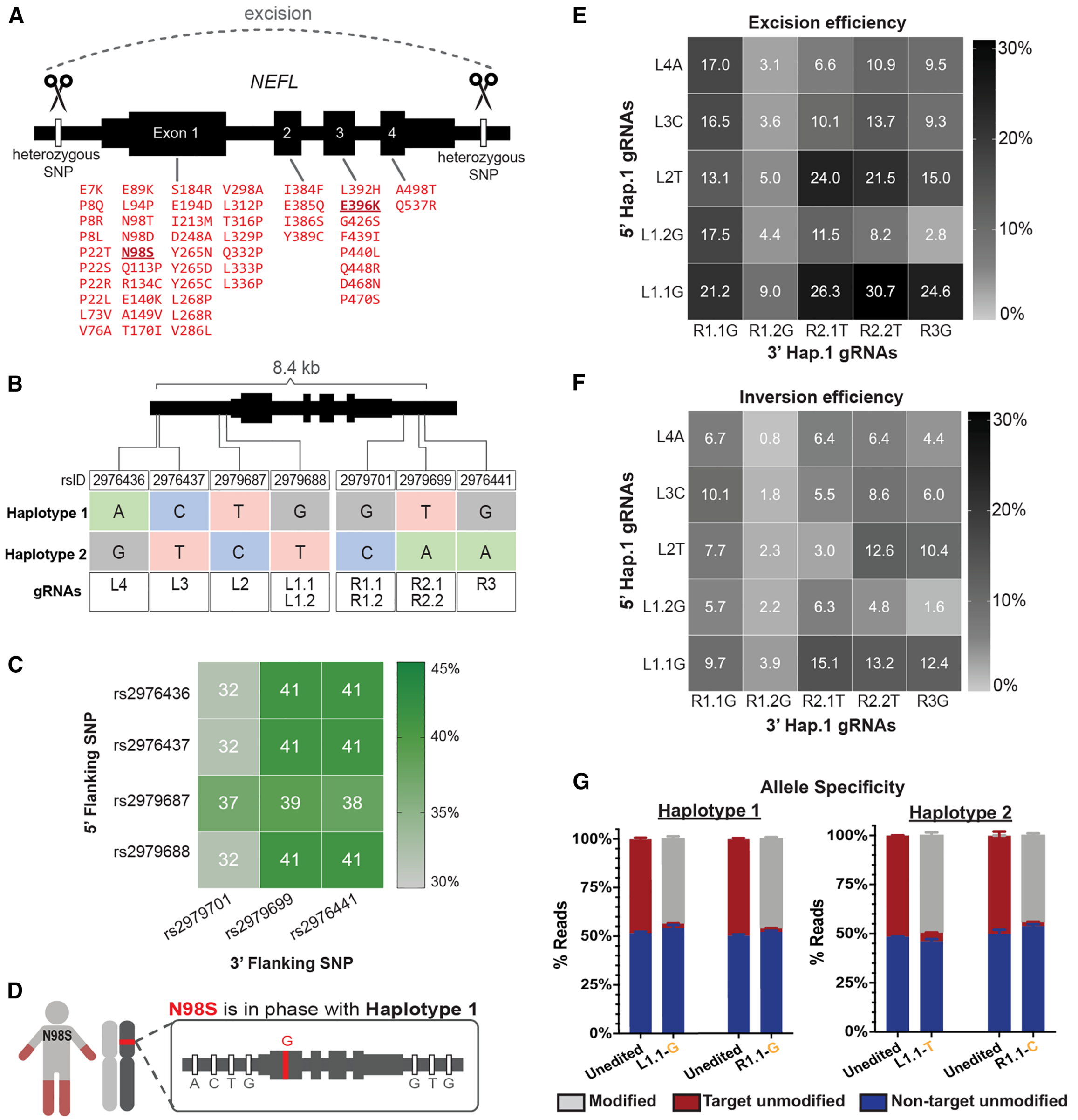

Figure 1. Common SNPs flanking the *NEFL* locus can be targeted to excise the N98S dominant allele

(A) Schematic of mutation-agnostic approach to excise any pathogenic *NEFL* mutation (indicated in red) using pairs of SNP-targeting gRNA-HiFiCas9 (scissors). (B) gRNAs targeting SNPs immediately 5′ (L) or 3′ (R) of *NEFL* and numbered by increasing distance from the gene body. Decimal numbers distinguish gRNA targeting the same SNP using different protospacer-adjacent motifs. Variant sequences shown define two haplotypes present in each heterozygous cell line. (C) Heatmap showing the percentage of individuals from 1kGP heterozygous for pairs of SNPs targeted by our panel of gRNAs. (D) Schematic of the mutation in N98S-P2 in phase with SNPs defining haplotype 1. (E and F) Heatmaps of excision (E) and inversion (F) frequencies measured by ddPCR after paired gRNA-HiFiCas9 transfections in N98S-P2 iPSCs. Data represent the mean of duplicate transfections. (G) Quantification of allele-specific editing with individual gRNAs via NGS amplicon sequencing. Graphs represent mean ± SEM of independent transfections (n = 4).

and that new mutations continue to be discovered, we reasoned that determining the mechanism of low editing efficiency and optimizing gRNAs for each possible mutation would be a laborious and impractical approach. We therefore looked for an alternative approach that would be allele specific but also mutation agnostic.

Haplotype-specific gRNA pairs excise the *NEFL*-N98S allele with varying efficiency

To find other ways to inactivate *NEFL* mutant alleles, we examined whole-genome sequencing of our four patient cell lines. We uncovered many heterozygous variants flanking *NEFL* in three out of four

of our cell lines, excluding N98S-P1 (Figure S4). Since these variants are in non-coding intergenic regions, indels at any one of the variant sites alone would be unlikely to inactivate the allele. Therefore, we designed gRNAs to target pairs of variants flanking *NEFL* with the goal of excising the entire mutant gene (Figure 1B). We validated the relevance of these allele-specific gRNAs for the broader human population by analyzing phased genomes from the 1000 Genomes Project (1kGP).[40] We found that 32%–41% of individuals are heterozygous for pairs of common single-nucleotide polymorphisms (SNPs) that could be targeted for allele-specific gene excision (Figure 1C). Finally, we determined that the disease mutation in N98S-P2 is in phase with the reference allele for each variant, which we refer to as haplotype 1 (Figure 1D).

We tested the haplotype excision strategy first with N98S-P2 so we could compare it with our original N98S mutation-targeting strategy. We paired each gRNA upstream of *NEFL* with each downstream gRNA and transfected N98S-P2 iPSCs with the resulting SNP-specific HiFiCas9 RNPs. We measured the frequency of excision by droplet digital PCR (ddPCR) using our established methodology[27] and found that it varied from 2.8% to 30.7% depending on the gRNA pair (Figure 1E). We also detected measurable levels of inversion in the same samples, ranging from 0.8% to 15.1% (Figure 1F). The most efficient combinations produced combined excision and inversion editing at >40% of total alleles, with 50% being the maximum expected if editing is perfectly specific for the target allele. Pairs containing the L1.1G gRNA induced high levels of both excision and inversion, whereas pairs containing the R1.2G gRNA induced remarkably low levels of both. We recognized that the design of our ddPCR assay would fail to detect larger than expected excisions or other editing events that disrupt binding of the primer or probe target sequences. Loss-of-signal ddPCR can quantify unexpectedly large deletions in single-guide RNA (sgRNA) editing experiments,[41,42] so we compared the results of our gain-of-signal excision assay to a loss-of-signal assay for 13 different gRNA pairs. We observed a consistent trend toward higher excision rates with the loss-of-signal assay, consistent with the presence of larger than expected excisions in all edited samples (Figure S5). The magnitude of difference was variable across samples, with a modest discrepancy in the sample with the highest editing efficiency: 21.2% vs. 26.9% for L1.1G-R1.1G (Figure S5). We previously showed that a loss-of-signal assay is less accurate and tends to overestimate excisions when the frequency is less than 10%, which may contribute to the larger discrepancy for some of the lower-efficiency combinations.[27]

We reasoned that producing the smallest possible excision would be advantageous for minimizing the risk of unintended adverse effects. Since the L1.1G-R1.1G was among the highest in total editing efficiency (21.2% excision and 9.7% inversion; Figures 1E and 1F), we performed additional evaluation of the gRNA targeting these SNPs closest to the 5′ and 3′ ends of *NEFL*. To validate allele specificity of the individual gRNA targeting each haplotype, we transfected N98S-P2 or E396K-P2 iPSCs with RNPs targeting their corresponding mutant haplotype and measured indels via deep amplicon sequencing, followed by analysis using CRISPResso2. This program includes a quantification feature that deconvolutes sequencing reads by allele based on the sequence of the heterozygous variant harbored by each allele.[43] Both haplotype versions of L1.1 and R1.1 were efficient at producing indels, with no evidence of editing at the non-target allele (Figures 1G and S6). Interestingly, both haplotype versions of L1.2 induced significant editing of the non-target allele despite the SNP being located in the protospacer-adjacent motif (PAM)-proximal seed region (Figure S6) known to be important for single-nucleotide specificity.[44] Both haplotype versions of R1.2 induced fewer indels than R1.1 (Figure S6C), consistent with the low excision and inversion frequencies we observed using R1.2G in the pairwise array (Figures 1E and 1F). Overall, these findings show that efficient and specific haplotype editing with L1.1 and R1.1 gRNA-HiFiCas9 is feasible at the *NEFL* locus. Next, we sought to investigate the therapeutic potential of this strategy in our i^{3}LMN disease model of CMT2E.

Haplotype-specific excision of N98S and E396K alleles restores NfL subcellular distribution in patient iPSC-derived motor neurons

To examine the effect of allele-specific haplotype editing on *NEFL* mRNA expression, we transfected N98S-P2 iPSCs with L1.1G and R1.1G RNPs targeting haplotype 1 (Figure 2A) and differentiated them into i^{3}LMNs. We measured mRNA transcript levels by quantitative RT-ddPCR and found that the edited population had decreased total *NEFL* mRNA compared with the unedited control, with an expected decrease in the fraction of mutant mRNA (Figures 2B and 2C). To determine the effect of excision vs. inversion on *NEFL* expression, we isolated clonal iPSC lines with excision or inversion of the N98S allele and differentiated them into i^{3}LMNs. As expected, neurons derived from the excision clone showed no expression of the N98S allele and an overall 50% decrease in total *NEFL* mRNA (Figures 2B and 2C). Interestingly, neurons derived from the inversion clone demonstrated minimal change in total or mutant *NEFL* mRNA, suggesting that inversion induced by this CRISPR editing strategy fails to separate the gene's coding region from its promoter and other regulatory elements.

The mutation in E396K-P1 and E396K-P2 is in phase with the alternate alleles, which we refer to as haplotype 2 (Figure S7A). To confirm that haplotype editing is also effective at targeting haplotype 2, we repeated the experiment in E396K-P2 iPSCs, using the L1.1T and R1.1C gRNAs specific to haplotype 2 (Figure S7B). We differentiated the pooled population of edited E396K-P2 iPSCs into i^{3}LMNs and again observed decreased total *NEFL* mRNA compared with the unedited control, with a corresponding decrease in the fraction of mutant mRNA (Figures S7C and S7D). When examining edited clones with excision or inversion, we confirmed our previous finding that the excision abolished expression of the mutant E396K allele and reduced total *NEFL* mRNA by 50%, while the inversion produced minimal changes in mutant or total *NEFL* expression (Figures S7C and S7D).

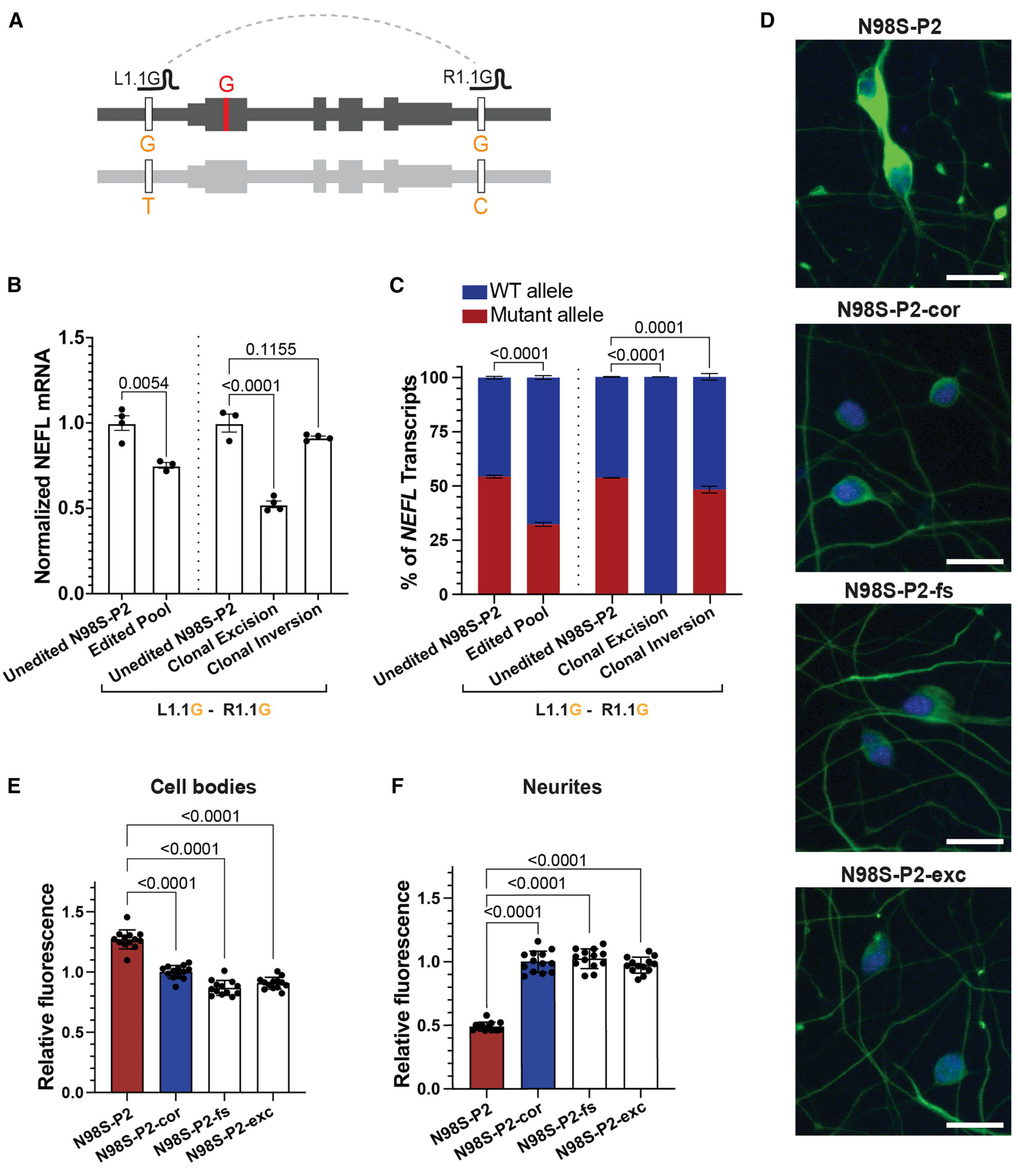

Figure 2. Allele-specific excision of *NEFL* inactivates mutant allele expression and rescues phenotypes in patient iPSC-motor neurons

(A) Schematic of SNP-targeted excision of N98S allele. Edited iPSCs from patient N98S-P2 were differentiated into i^3LMNs; RNA isolated on day 7 and measured by quantitative RT-ddPCR. (B) Total *NEFL* expression relative to *GAPDH* and normalized to unedited control. (C) Relative allelic expression as determined by ddPCR using a heterozygous SNP in the 3′ UTR (rs2976439). Bar graphs for (B) and (C) indicate mean ± SEM normalized to unedited control, with each data point representing an independent well of i^3LMNs (n = 3–4). Dashed vertical lines indicate separate experiments for a mixed population of edited cells vs. clones with specific editing outcomes. Comparisons were performed by unpaired two-tailed t test; multiple comparisons were performed by one-way ANOVA with Dunnett's post-test. (D) Representative images of day 7 i^3LMNs stained with anti-NfL (green) and anti-HB9 (blue) antibodies. Scale bars, 20 μM. (E) Quantification of mean NfL fluorescence intensity in i^3LMN cell bodies from clones with specified editing outcomes. (F) Quantification of total NfL fluorescence intensity in i^3LMN neurites normalized to β3-tubulin$^+$ neurite area. Bar graphs for (E) and (F) represent mean fluorescent intensity ± SEM from 15 independent wells of i^3LMNs normalized to isogenic corrected control (N98S-P2-cor). Each data point represents the mean value from 15 images per well of i^3LMNs. Multiple comparisons were done by one-way ANOVA with Dunnett's post-test.

One manifestation of *NEFL*-associated pathology is the accumulation of neurofilaments in the cell body of motor neurons.[10,15,25] To determine whether allelic inactivation via excision rescued this phenotype, we stained i^{3}LMNs derived from N98S-P2 iPSCs for NfL and the nuclear motor neuron marker HB9 and measured the NfL fluorescence signal with our established image analysis pipeline.[10] We compared N98S-P2 excision to the isogenic frameshift, corrected, and unedited controls, and found that excision was as effective as frameshift at normalizing NfL intensity in motor neuron cell bodies (Figures 2D and S8A). We performed the same analysis comparing E396K-P2 excision with E396K-P2 inversion, unedited control, and an unrelated healthy control line (wild-type C [WTC]). We observed a pattern similar to that in the N98S series, although the E396K phenotype was more subtle (Figures S8B and S8C), consistent with functional comparisons of the effects of these mutations on neurofilament assembly.[22]

In N98S mutant mice, aggregation of neurofilaments in neuronal cell bodies is associated with severe depletion of neurofilaments in axons.[16] We questioned whether we could detect a similar neurofilament protein depletion in neurites of our motor neurons and whether inactivation of the mutant allele could restore this distribution despite the decrease in total transcript levels. To test this hypothesis, we developed a protocol to simultaneously measure NfL in cell bodies and neurites from a single imaging assay by including anti-β3-tubulin to identify neurites and replacing anti-HB9 with a general nuclear stain to identify cell bodies (Figures S9 and S10). Furthermore, we incorporated a fully automated microscopy platform to increase the throughput and content of our dataset in an unbiased manner. We designed complementary CellProfiler[45] pipelines to quantify fluorescence intensity in the cell bodies and neurites from this imaging platform (Figures S9 and S10). This analysis recapitulated our finding that frameshift and excision of the N98S allele similarly rescue the pathologic accumulation of NfL in the cell body, confirming the validity of this assay (Figure 2E). Consistent with the *in vivo* results from mice, we observed a dramatic depletion of NfL in N98S neurites compared to the isogenic corrected control (Figure 2F). Furthermore, excision or frameshift inactivation of N98S restored NfL levels within neurites (Figure 2F). We previously showed that inactivation of *NEFL*-N98S leads to reduced total NfL protein in i^{3}LMNs.[10] Our findings here demonstrate that the reduced accumulation of NfL in cell bodies also reflects redistribution to other cellular locations, with likely important functional consequences.

To evaluate whether off-target genome editing could confound the biological behavior of edited clones, we sequenced bioinformatically predicted off-target sites in each clonal line derived from N98S-P2 and E396K-P2 and did not detect evidence of CRISPR-induced mutations (Table S1). Additionally, we determined that the excision or inversion of *NEFL* did not affect the expression of *NEFM*, which encodes the co-regulated neurofilament-medium chain that is adjacent to the *NEFL* locus (Figure S11). Finally, we performed electrophysiological analysis on differentiated motor neurons using a multi-electrode array. Spontaneous action potentials were similar in all excision and inversion clones and their unedited N98S-P2 and E396K-P2 controls, suggesting that haplotype editing did not disrupt basic neuronal electrophysiological function (Figure S12).

Including a single-stranded bridging oligonucleotide boosts excision efficiency by 30%–50% in proliferating iPSCs

Although haplotype editing at our target SNP sites induced significant levels of both excisions and inversions, only the excision inactivated the mutant allele. Therefore, we investigated methods to increase the frequency of excision outcomes. Previous work indicated that a 100-nt single-stranded oligonucleotide donor (ssODN) could induce long-range excision in the context of a single DSB induced by zinc-finger nucleases.[46] Reasoning that a similar strategy might also enhance excisions produced by paired Cas9 RNPs, we transfected N98S-P2 and E396K-P2 iPSCs with L1.1 and R1.1 RNP and included a 60-nt ssODN designed to mimic the excision repair outcome (Figure 3A). We measured both excision and inversion frequency by ddPCR and found a nearly 50% increase in excision frequency in N98S-P2 iPSCs and a nearly 33% increase in E396K-P2 iPSCs, while inversion frequency showed no significant change in either patient line (Figure 3B). We also investigated indel outcomes at the individual target sites and found that they varied inconsistently with the addition of the bridging oligo (Figure S13A). We were unable to detect excision when combining the 60-nt bridging ssODN with either sgRNA-HiFiCas9 RNP alone (Figure S13B) despite the sensitivity of our ddPCR assay to detect excision at a frequency of 0.1%.[27] The discrepancy between our results and the previous published study could be due to differences in the types of cell lines, ssODN design, and nucleases used for the experiments.

To confirm that the effect of the bridging ssODN is not unique to this specific excision outcome, we repeated the experiment with a smaller excision removing only the first exon. We observed a similar increase in excision efficiency and no effect on inversion (Figure S13C). We decreased the length of the ssODN to 30 nt, which eradicated the boost in efficiency, while increasing the length of the ssODN to 120 nt or adding stabilizing chemical modifications did not significantly improve the frequency of excisions (Figures S13D and S13E). We next investigated whether the addition of the bridging ssODN alters the nature of the excision repair junction, using next-generation sequencing (NGS) amplicon sequencing analysis. The single most common excision outcome in both N98S-P2 and E396K-P2 cell lines was the precise expected repair junction (45.7% and 31.8% of all events, respectively), followed by a 5-nt larger than expected excision consistent with microhomology-mediated repair (23.8% and 21%, respectively), and single-nucleotide additions or deletions at the repair junction (10.7% and 11.8%, respectively; Figure S13F). Inclusion of the 60-nt bridging ssODN increased precise excision events (58.1% and 49.5%, respectively; Figures 3C and S13F), while the presumed microhomology-mediated repair outcome was significantly decreased (17% and 13.1%, respectively). Single-nucleotide indels were not significantly changed by inclusion of the ssODN (Figure S13F).

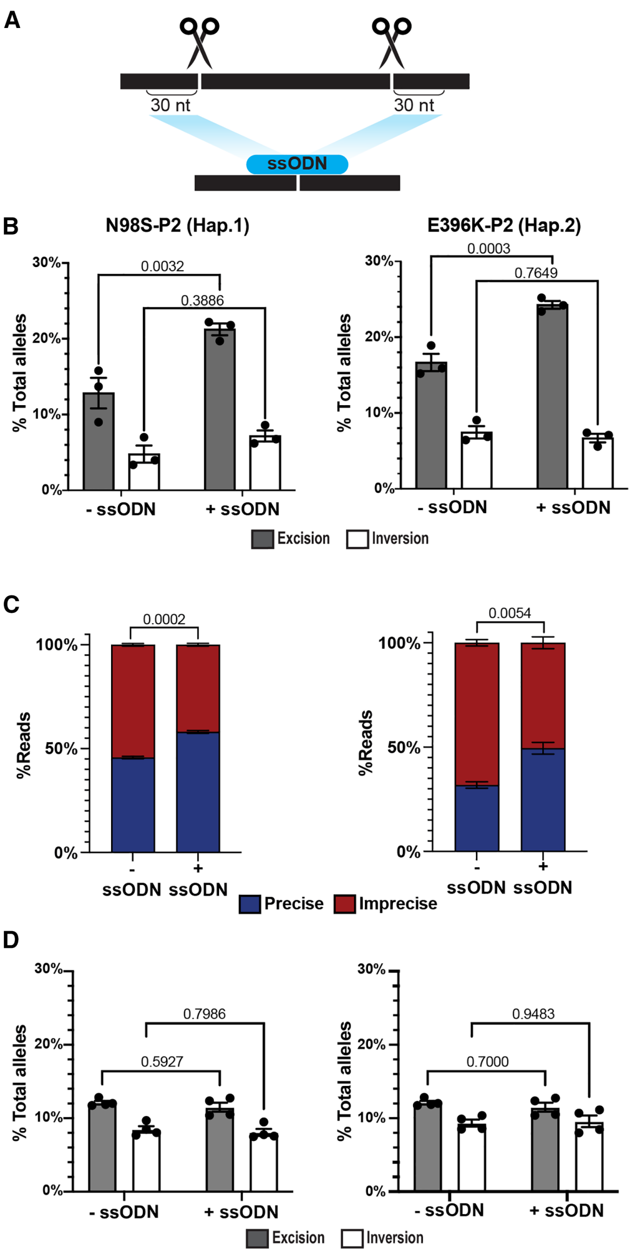

Figure 3. Excision frequency and precision is increased by single-stranded oligonucleotide bridging donors in iPSCs

(A) Schematic of bridging single-stranded oligonucleotide (ssODN) design complementary to the expected excision repair product. (B) Excision and inversion frequencies ± ssODN quantified by ddPCR after transfection of L1.1-R1.1 RNPs in iPSCs. (C) NGS amplicon sequencing of excision junctions in iPSCs ± ssODN with quantification of precise and imprecise excision repair events. (D) Excision and inversion frequencies ± ssODN quantified by ddPCR after transfection of L1.1-R1.1 RNPs in d3 i^{3}LMNs. Bar graphs represent mean ± SEM with individual data points representing independent transfections (*n* = 3–4). Comparisons of multiple variables in (B)–(D) were performed by two-way ANOVA, followed by Šídák's test for selected variables.

To investigate the potential relevance of this method for haplotype excision in neurons, we sought to determine whether a bridging ssODN can increase excision frequency in postmitotic cells. For this experiment, we leveraged the dissociation step at day 3 in the i^{3}LMN differentiation protocol, which allowed us to study editing outcomes in immature but postmitotic i^{3}LMNs using the same reagents and delivery methods as for iPSCs. We nucleofected L1.1 and R1.1 RNP ± a 60-nt ssODN in N98S-P2 and E398K-P2 day 3 i^{3}LMNs, followed by analysis on day 7. We observed a similar baseline level of excision and inversion editing compared to iPSCs but with no significant effect of the ssODN on either outcome (Figure 3D). At day 3, there is a minor population of dividing cells that are eliminated through the addition of bromodeoxyuridine (BrdU).[38] To rule out the possibility that BrdU could interfere with the effect of the ssODN during DNA repair, we repeated the experiment while omitting BrdU from the day 3 media and again observed no effect from the ssODN (Figure S14A). Finally, we used NGS amplicon sequencing to examine the excision repair junctions after editing N98S-P2 and E396K-P2 day 3 i^{3}LMNs, which revealed a more marked predominance of precise repair events than is seen in iPSC (66.2% and 81.9%, respectively; Figure S14B). There was a small but statistically significant increase in precise excision events upon addition of the ssODN in both cell lines to 74.1% and 84.6%, respectively (Figure S14B). Our results indicate that the simple addition of a 60-nt ssODN can increase both the efficiency and the precision of large DNA excisions in iPSCs, but with limited effect in postmitotic cells. Thus, we pursued alternative strategies to enhance productive editing outcomes in our system.

Incorporating a bi-allelic intronic gRNA enables both excisions and inversions to inactivate the mutant allele

We hypothesized that mutant *NEFL* expression was unchanged after L1.1-R1.1 inversion because the transcription start site (TSS) remains linked with its core promoter. To increase the efficacy of our editing approach, we sought to design gRNA pairs that would cause both excisions and inversions to disrupt expression of the mutant alleles. We reasoned that we could achieve this goal by combining either one of our flanking gRNAs (L1.1 or R1.1) with an intragenic gRNA (Figure 4A). However, intragenic SNPs are less common in the population and our cell lines, limiting our ability to design allele-specific intragenic gRNAs. Thus, we designed gRNAs common to both alleles (bi-allelic) in the first intron of *NEFL* and paired them with our SNP-specific gRNAs for partial gene excision and inversion (Figure 4A). Since bi-allelic gRNAs are expected to induce indels on the WT allele with potentially deleterious effects on gene expression and splicing,[47] we first assessed whether indels in the targeted region of the intron would affect *NEFL* expression. We tested two bi-allelic gRNAs in the first intron that generated indels at up to 89% efficiency (Figure S15A). Differentiation of the edited populations to i^{3}LMNs demonstrated maintained *NEFL* expression despite the high frequency of intronic indels (Figure S15B). This analysis used an assay designed to detect transcripts with normal splicing of exon 1 to

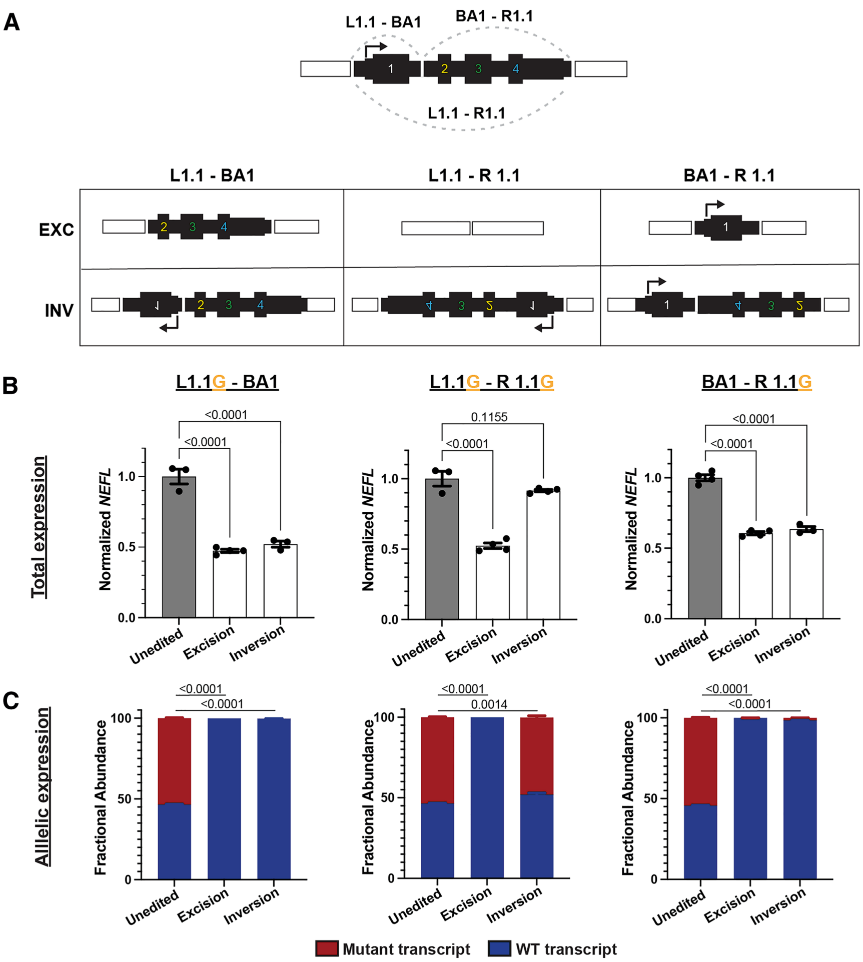

Figure 4. gRNA pairs that include an intronic gRNA induce excisions and inversions that both inactivate mutant alleles

(A) Schematic of predicted editing outcomes with various combinations of L1.1, R1.1, and intronic bi-allelic (BA1) gRNAs. N98S-P2 was transfected with each RNP pair, and clonal iPSC lines were isolated with each predicted outcome and differentiated into i^{3}LMNs. RNA was isolated on day 7 and measured by quantitative RT-ddPCR. (B) Total *NEFL* expression relative to *GAPDH* and normalized to unedited control. (C) Relative allelic expression was assessed via allele discrimination ddPCR using a heterozygous SNP in the 3′ UTR (rs2976439). Bar graphs represent the mean ± SEM, with each individual data point representing an independent well of i^{3}LMNs (n = 3–4). Multiple comparisons were performed by one-way ANOVA with Dunnett's post-test.

exon 2, suggesting that neither transcription nor splicing of *NEFL* were impaired by indels at the intronic target sites.

Next, we paired the bi-allelic gRNA with highest indel-inducing efficiency (BA1) with each of the SNP-specific gRNAs we used previously (L1.1 or R1.1) to determine whether partial gene excision and inversion would lead to mutant allele inactivation. In the case of the L1.1-BA1 excision, the TSS and entire first exon would be deleted, whereas inversion would disrupt the orientation of the TSS and first exon from the rest of the gene. In the case of the BA1-R1.1 excision, the final three exons would be deleted, whereas inversion would disrupt the orientation of the final three exons, including the translation and transcription terminator sequences (Figure 4A). We transfected N98S-P2 iPSCs with RNP containing the L1.1G-BA1 gRNA pair and the BA1-R1.1G gRNA pair, isolated clones with either excision or inversion, and differentiated these clones into i^{3}LMNs. With both L1.1-BA1 and BA1-R1.1 conditions, excision led to 50% reduction in total *NEFL* mRNA and no detectable expression of the N98S allele (Figures 4B and 4C). More notably, inversion also led to a 50% reduction in total *NEFL* transcripts and no detectable expression of the mutant allele. We applied this strategy to the E396K line and obtained comparable results (Figure S16).

High-efficiency and high-specificity therapeutic editing can be achieved with a bi-allelic gRNA paired with an allele-specific gRNA

Our ultimate goal is to optimize an allele-specific editing strategy that produces the most therapeutic events (i.e., inactivation of the mutant *NEFL* allele) with the lowest risk of editing the normal allele. To this end, we first compared the efficiency and specificity of edits generated with L1.1-BA1, L1.1-R1.1, and BA1-R1.1. We transfected each of these three gRNA pairs into N98S-P2 and E396K-P2 iPSCs (Figure 5A) along with a 60-nt bridging ssODN designed to mimic each predicted excision outcome (see Table S2) and measured the excision and inversion efficiency by ddPCR in polyclonal iPSC populations. In both N98S-P2 and E396K-P2 iPSCs, the pairs that included the bi-allelic gRNA produced more combined excisions plus inversions than did the pair of allele-specific gRNAs (Figure 5C). Notably, the gRNA pair L1.1G-BA1 in N98S-P2 produced combined excision and inversion edits in approximately 60% of alleles. Since allele-specific editing should ideally occur on a maximum of 50% of total alleles, we hypothesized that the introduction of a bi-allelic gRNA was leading to undesired excision and/or inversion of the non-target allele. Thus, we sought to thoroughly investigate the specificity of each gRNA combination.

To rigorously measure specificity, we developed new variations of our ddPCR-based methodology. We designed PCR assays to amplify each excision and inversion breakpoint along with a flanking SNP; the amplified fragments were isolated by gel electrophoresis and used as the template for allele-discrimination ddPCR (Figures 5B

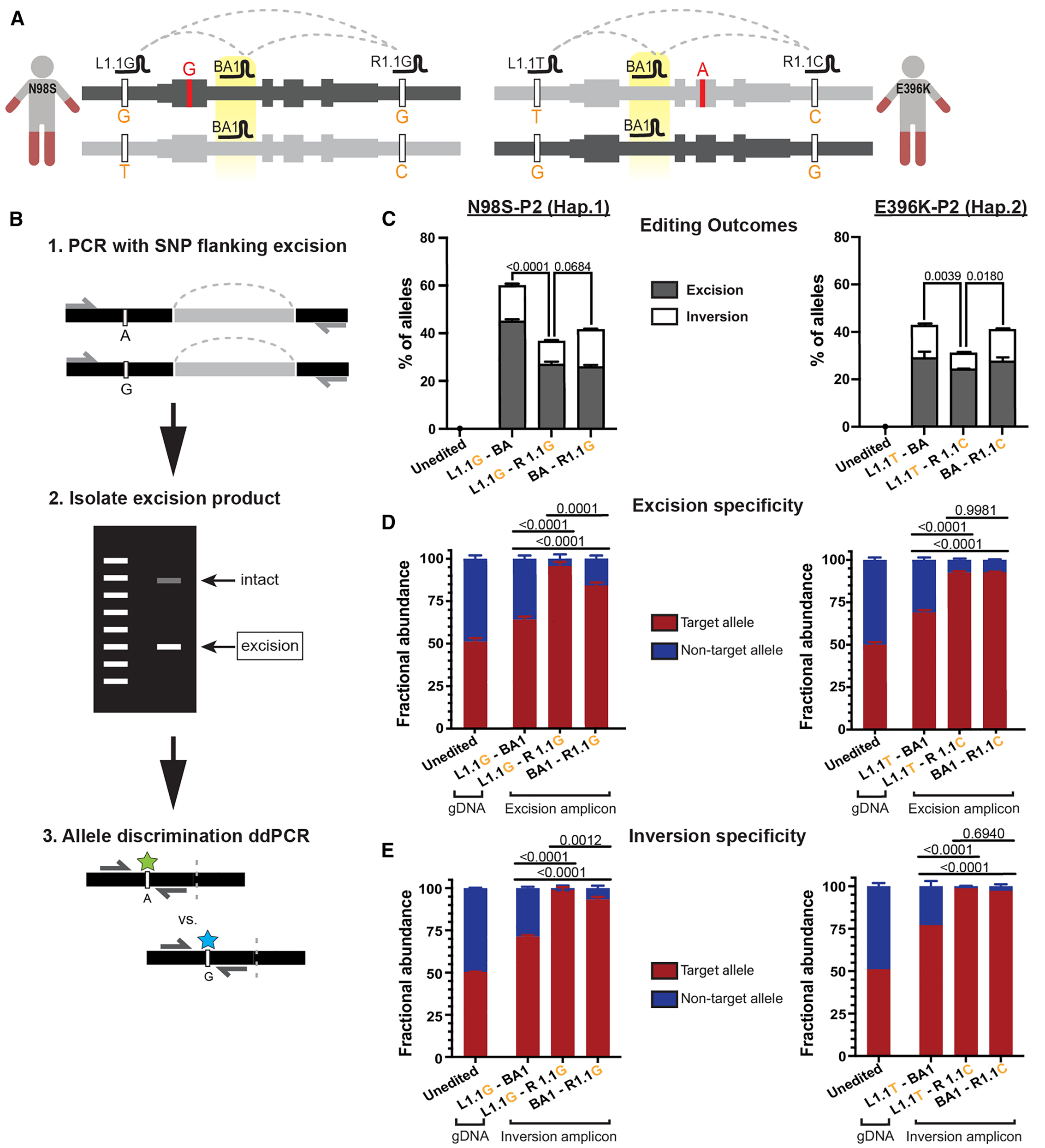

Figure 5. Optimization of allele-specific haplotype editing in CMT2E patient iPSCs

(A) Schematic of haplotype-specific editing in N98S-P2 (haplotype 1, dark gray) and E396K-P2 (haplotype 2, light gray) with various combinations of L1.1, R1.1, and intronic bi-allelic (BA1) gRNAs. A bridging ssODN designed to match the unique excision junction was included in each transfection. (B) Schematic of assay design and workflow for measurement of excision specificity. (C) Quantification of excision and inversion frequency via ddPCR. (D and E) Quantification of allele specificity of excision and inversion via a ddPCR allele discrimination assay to a linked heterozygous SNP (rs2979685) located 5′ of the *NEFL* gene. Unedited controls demonstrate equal abundance of variant alleles in gDNA at baseline, while edited samples represent fractional abundance of alleles in excision or inversion events. All graphs represent mean ± SEM of independent transfections (n = 3–4). Multiple comparisons were performed by one-way ANOVA with (C) Dunnett's post-test and (D and E) Tukey's post-test.

and S17). As expected, excision and inversion with the combination of two allele-specific gRNAs were highly specific for the target haplotype in both cell lines, with 92%–99% of edits occurring on the target allele (Figures 5D and 5E). However, pairing L1.1 with BA1 resulted in a significant decrease in the specificity of either excision or inversion to 64%–77%. This result is consistent with the higher overall editing efficiency we observed with this pair (Figure 5B). Interestingly, the specificity of excision and inversion was relatively preserved when pairing R1.1 with BA1 (84%–97%). In particular, targeting haplotype 2 in the E396K-P2 cell line with BA1-R1.1C

A

	N98S-P2 iPSCs		N98S-P2 D3 iLMNs	
Excision Frequency (%)	12.7	18.1	16.4	15.0
Inversion Frequency (%)	6.7	10.9	17.4	13.8
**Total Haplotype Editing (%)**	**19.4**	**29.0**	**33.8**	**28.8**
**Excision Specificity**	**89.8**	**92.3**	**91.0**	**92.0**

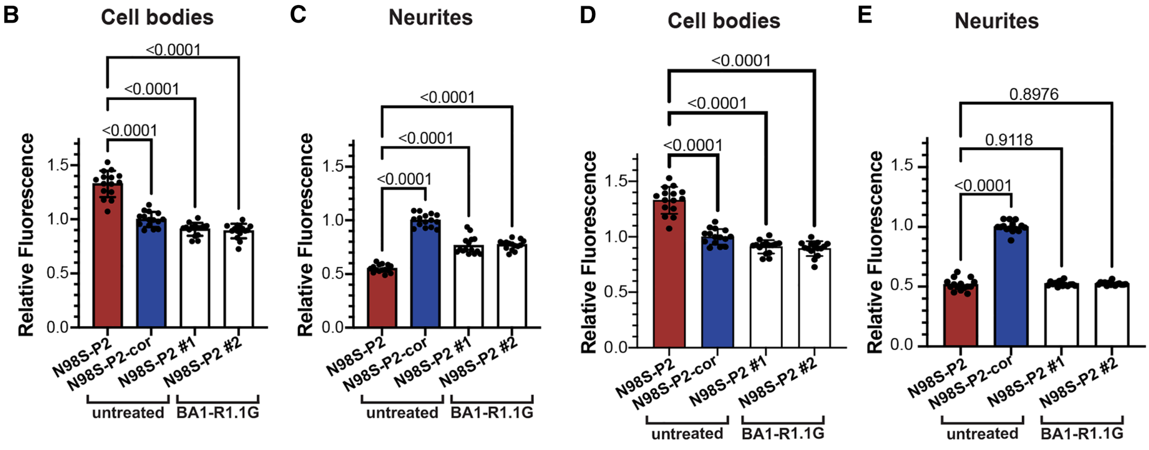

Figure 6. Modeling therapeutic haplotype editing in iPSC pools and day 3 i^{3}LMNs

N98S-P2 iPSCs were transfected in duplicate with BA1-R1.1G and maintained as two independent edited pools, followed by differentiation to i^{3}LMN until day 7. Separately, N98S-P2 day 3 i^{3}LMNs were transfected in duplicate with BA1-R1.1G, followed by continued differentiation to day 7. In both cases, unedited N98S-P2 and N98S-P2-cor clones were differentiated in parallel as controls. (A) Haplotype editing efficiency and specificity was measured by ddPCR in N98S-P2 day 7 i^{3}LMNs after nucleofection of iPSC or day 3 i^{3}LMNs. Each column represents a sample from an independent edited population. (B) Quantification of mean NfL fluorescence intensity in day 7 i^{3}LMN cell bodies after editing in iPSC. (C) Quantification of total NfL fluorescence intensity in day 7 i^{3}LMN neurites normalized to β3-tubulin$^+$ neurite area after editing in iPSC. (D) Quantification of mean NfL fluorescence intensity in day 7 i^{3}LMN cell bodies after editing in day 3 i^{3}LMNs. (E) Quantification of total NfL fluorescence intensity in day 7 i^{3}LMN neurites normalized to β3-tubulin$^+$ neurite area after editing in day 3 i^{3}LMNs. Bar graphs for (B)–(E) represent mean fluorescent intensity ± SEM from 15 independent wells of i^{3}LMNs, normalized to isogenic corrected control (N98S-P2-cor). Each data point represents the mean value from 15 images per well of i^{3}LMNs. Multiple comparisons were done by one-way ANOVA with Dunnett's post-test.

showed equivalent specificity to the allele-specific pair L1.1T-R1.1C (Figures 5D and 5E), indicating that a single SNP-specific gRNA paired with a bi-allelic gRNA can be sufficient for highly allele-specific excision and inversion in some cases. Importantly, the differential specificity of L1.1 and R1.1 for excision and inversion when combined with BA1 was not predicted by testing the specificity of the individual gRNAs (Figure 1G) and was only revealed by our novel assay.

To confirm these results with a separate complementary assay, we designed a multiplexed digital PCR assay with four differentially labeled probes (Figure S18). This assay recapitulated our previously described method utilizing the linkage of an allele-discrimination assay to an excision-specific amplicon to measure allele specificity in excisions[27] but with greater precision due to the use of non-overlapping fluorophores. Furthermore, the multiplexed digital PCR allowed us to simultaneously quantify efficiency and specificity. The results corroborated data from the previous separate assays for efficiency and specificity in a single-step reaction without gel electrophoresis (Figures S18 and S19). This method allows the complete assay to be completed in 96-well format in 2 h, enabling high-throughput arrayed screens to optimize haplotype editing reagents for both efficiency and specificity.

We hypothesized that the improvements in disruptive haplotype editing with the BA1-R1.1 combination could lead to phenotypic improvement in edited i^{3}LMNs without clonal selection, and selected N98S-P2 to model this therapeutic editing strategy due to its more robust phenotype. We performed two independent nucleofections of N98S-P2 iPSCs with BA1-R1.1G + ssODN, followed by differentiation of the pooled populations to i^{3}LMNs, along with N98S-P2 and N98S-P2-cor as controls. Excision efficiency in day 7 i^{3}LMNs was 12.7% and 18.1% in the two pools, with an inversion efficiency of 6.7% and 10.9%, respectively. Specificity for excision of the target haplotype was 89.8% and 92.3%, respectively (Figure 6A). Analysis of day 7 i^{3}LMNs using our established CellProfiler pipeline demonstrated that abnormalities in NfL intensity in both neurites and cell bodies were significantly ameliorated in the edited pools compared with unedited N98S-P2 (Figures 6B and 6C). NfL intensity in the cell bodies of edited pools was decreased to levels as low as the isogenic corrected control, while the neurite NfL intensity was partially restored. Finally, we tested

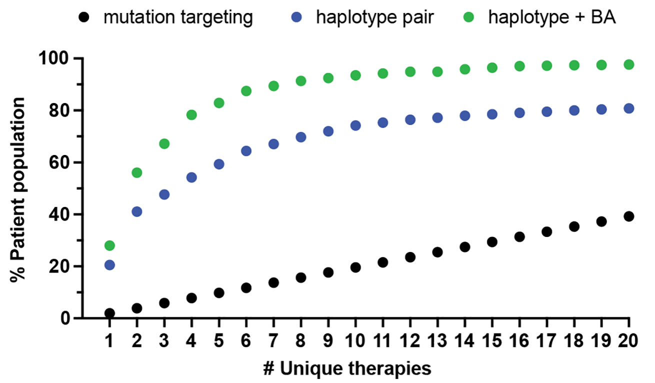

Figure 7. Calculation of patient population coverage with haplotype editing as compared to mutation-targeted editing

Data points illustrate the cumulative number of patients treated with the addition of increasing numbers of unique molecular therapies. Black circles represent mutation-targeted Cas/gRNA and assume 51 pathogenic mutations in *NEFL* that are equally prevalent. Blue circles represent haplotype excision with two SNP-targeting Cas/gRNAs. Green circles represent haplotype excision with one SNP-targeted Cas/gRNA and a constant bi-allelic Cas/gRNA. Combinations that would excise the neighboring *NEFM* gene are excluded from this analysis.

whether we could achieve comparable levels of haplotype editing and phenotypic rescue via editing in day 3 i^3LMNs. We performed two independent nucleofections of N98S-P2 day 3 i^3LMNs and continued the differentiation until day 7, along with unedited N98S-P2 and N98S-P2-cor as controls. Excision efficiency was 16.4% and 15% in the two pools, with an inversion efficiency of 17.4% and 13.8%, respectively. Specificity for excision of the target haplotype was 91% and 92%, respectively (Figure 6A). In this experiment, we again detected a significant decrease in NfL intensity in cell bodies, although the NfL intensity in neurites remained unchanged (Figures 6D and 6E). In summary, we demonstrated that we can achieve similar haplotype editing efficiency and specificity in iPSC and in post-mitotic day 3 i^3LMNs, resulting in measurable changes in protein distribution in bulk populations of neurons.

Leveraging common haplotypes and paired gRNA editing considerably reduces the number of gRNA-Cas combinations needed to treat the majority of CMT2E patients

To quantify the power of haplotype editing for *NEFL,* we used data from the 1kGP[40] and calculated the percentage of CMT2E patients that could be treated with a given number of unique allele-specific therapies. For mutation-targeted editing, we assumed that there are 51 mutations (Figure 1A) that occur with similar frequency in the patient population, since the relative prevalence of different mutations causing CMT2E has not been systematically defined. For haplotype editing, we assumed that SNPs are distributed similarly in CMT2E patients as in the general human population and calculated the percentage of individuals heterozygous for individual SNPs or pairs of SNPs flanking the entire gene (Figure S20). Finally, assuming that each mutation or SNP can be targeted with an optimized Cas/gRNA, we calculated the percentage of individuals who could be treated with increasing numbers of unique Cas/gRNA molecular therapies (Figure 7). Using mutation-specific gRNAs, 26 unique molecular therapies would be needed to treat >50% of patients, whereas for haplotype editing with common SNP-targeted gRNA, 4 unique therapies achieve a similar yield (Figure 7). The yield of haplotype excision is markedly improved through the incorporation of a constant reagent like our BA1 gRNA. Pairing BA1 with a single allele-specific Cas/gRNA flanking either side of the gene allows 4 unique therapies to treat >75% of patients.

DISCUSSION

Genetic disorders caused by autosomal-dominant missense mutations represent a significant challenge for gene therapy, which must counteract the disease allele without affecting the normal allele, even though the two differ by a single nucleotide. We have shown in our previous published work that allele-specific inactivation of the *NEFL* N98S mutation, a dominant missense mutation that causes CMT2E, was achievable with an engineered Cas9 and a mutation-targeting gRNA. This editing strategy rescued CMT2E pathology in patient-derived iPSC-motor neurons, the cell type most affected in the disease. The present study demonstrated that we can achieve similar success with pairs of gRNAs targeting common single-nucleotide variants flanking the mutant *NEFL* allele in a mutation-agnostic manner. We showed the validity of this approach for two separate *NEFL* dominant mutations (N98S and E396K) linked to the most common haplotypes in the human population. By taking advantage of naturally occurring and commonly inherited variants, our haplotype editing approach circumvents the need to develop bespoke therapies for each missense mutation, greatly reducing the number of therapies needed to treat a majority of the CMT2E patient population. For simplicity, our comparison of haplotype editing to mutation targeting (Figure 7) assumed equal prevalence of each mutation in the population. In the clinical literature, N98S and E396K have been reported more frequently than other *NEFL* mutations,[48] although these data are subject to multiple sources of bias that confound definitive measurement of their relative prevalence. Furthermore, our analysis of the mutation burden for *NEFL* is likely a significant underestimate of the true number of pathogenic mutations, as there are 386 total *NEFL* missense variants currently reported in ClinVar (https://www.ncbi.nlm.nih.gov/clinvar).[49,50] Larger genes with an even more complex mutational burden are also compelling candidates for haplotype editing. For example, *MFN2* is the most common cause of CMT2, with 738 total missense mutations and 154 currently classified as pathogenic or likely pathogenic by ClinVar. We anticipate that our approach, using *NEFL* as a proof-of-concept example, can translate to a variety of other diseases.

The simplest haplotype editing strategy would be to target coding variants to selectively induce frameshift-producing indels on the desired allele, an attractive option for genes with frequent coding variants.[51] However, the majority of coding variants occur at low frequency in the human population, as we observed for *NEFL.*[52] In addition, frameshift editing can fail to achieve the desired outcome

for various reasons, including low indel rates that we observed when targeting E396K, or failure to induce nonsense-mediated decay of the transcript with a risk of generating truncated mutant proteins.[53] Excision is a more complex but also widely generalizable strategy to target the abundant non-coding variants in the human genome. Additionally, excision is not limited to coding exons but can be applied to various other sequences, such as intronic splicing mutations, repeat expansions, and regulatory elements.[28,29,33,54] Gene editing therapy for Leber congenital amaurosis 10 leverages the combination of excision and inversion for therapeutic outcomes,[28,29] but many other gene-editing studies using paired Cas9-gRNA have ignored the frequency or functional impact of inversions. Here, we showed that inversion is a frequent editing outcome with variable effects on gene expression. We identified multiple strategies to optimize therapeutic editing outcomes, including inversion, while also increasing generalizability for maximum impact on the patient population.

Other groups have utilized heterozygous variants for allele-specific editing, most of them focused on variants that generate a novel PAM to recruit the Cas enzyme to the intended allele.[34–37,51,55] Our strategy incorporated variant-specific sequences in the seed region of the gRNA protospacer to discriminate between alleles, allowing us to target both haplotypes by altering a single nucleotide in the otherwise identical gRNA. We showed that this strategy can achieve excellent specificity for both indel and excision editing, although we observed important gRNA-dependent variation. We tested a relatively small number of gRNAs using a single Cas enzyme with promising results, but there are thousands of potential SNP combinations to target for haplotype excision (Figure S20). Larger-scale experiments will be important to nominate the best reagents for therapeutic development and could provide insight into genetic and epigenetic factors that maximize the desired editing outcomes. This highlights the importance of our innovations to rigorously evaluate multiple candidates for efficiency and specificity in high throughput, especially as engineered Cas enzymes with minimal PAM requirements allow us to target a larger number of variant sequences.[56–58] Few previously published studies have evaluated allele-specific excision, and to our knowledge, no study has evaluated inversion specificity. Two studies used PCR and Sanger sequencing analysis to provide a qualitative assessment of excision specificity.[35,36] Another used deep amplicon sequencing to evaluate excision specificity by monitoring a heterozygous variant adjacent to the excision site, which provides a high degree of precision and sensitivity.[34] However, due to the limitations of short-read sequencing, only a small number of excisions they tested had variants in requisite proximity for this analysis. Our allele-discrimination digital PCR approach is conceptually similar and highly precise, but more versatile for monitoring haplotype-specific variants distal to the editing site. Furthermore, this novel method allowed us to achieve highly specific excisions and inversions using a single allele-specific gRNA, while revealing differential gRNA specificity that was otherwise undetectable. Our data highlight the utility of pairing an allele-specific gRNA with a universal bi-allelic gRNA targeting any non-coding locus tolerant of small indels. In addition to streamlining the process of therapeutic development (Figure 7), this strategy also creates opportunities to design editing strategies for genes with fewer heterozygous variants.

One limitation of our study for translation to therapeutic development for CMT2E is that most editing experiments were performed in iPSCs rather than directly in neurons. These methods may be directly applicable for therapeutic editing approaches in cells such as hematopoietic stem cells that are also proliferative and can be edited *ex vivo*. Editing in iPSC also conferred advantages for optimizing therapeutic editing in neurons, as it allowed us to rigorously evaluate independent editing outcomes through clonal isolation and functional testing in differentiated i^3LMNs. Importantly, we applied these improvements to achieve haplotype editing in post-mitotic day 3 i^3LMNs that was comparable to that in iPSCs, with measurable effects at the NfL protein level (Figure 6). The unchanged levels of NfL protein in neurites after editing in day 3 i^3LMNs may reflect a failure to achieve steady state given the short time interval and early phase of differentiation. Follow-up studies will be important to evaluate the reversibility and kinetics of NfL protein mis-localization in more mature neurons, especially given the long protein half-life.[59]

Despite similar overall editing efficiency in iPSCs and day 3 i^3LMNs, our data suggest that mechanisms of DNA repair during haplotype editing are likely to be significantly different in neurons (or other post-mitotic cells) compared with dividing cells. This is highlighted by the discrepant effects of ssODN on excision efficiency, differential contributions of specific excision repair outcomes, and the ratio of excision to inversion (Figures 3 and 6). Consistent with this, our group has recently demonstrated that cell-type-specific DNA repair pathways also lead to differences in indel outcomes in iPSC-derived neurons.[60] Future studies should investigate opportunities to maximize the efficiency and specificity of haplotype editing in neurons, as well as evaluate the potential for adverse genomic effects, including chromosomal rearrangements and large deletions that have been observed with nuclease-based editing in other cell types.[41,61–64] In summary, the innovations described here provide a platform to optimize haplotype editing for the human genome to maximize safety and efficacy for the greatest number of patients. Ultimately, therapeutic application of our approach for CMT2E will also require experiments in animal models to optimize delivery vectors and editing reagents for spinal motor neurons *in vivo*.

MATERIALS AND METHODS

Identification of genomic variants and *NEFL* mutation phasing

We compiled a list of disease-causing missense mutations from two sources: (1) annotated in ClinVar[49] (https://www.ncbi.nlm.nih.gov/clinvar) as pathogenic or likely pathogenic and (2) annotated in the Inherited Neuropathy Variant Browser[65] (https://neuropathybrowser.zuchnerlab.net) with a publication or clinical report.

To identify variants from patient samples high-molecular-weight gDNA was isolated from N98S-P1, N98S-P2, E396K-P1, and E396K-P2 iPSC lines using the Blood & Cell Culture DNA Midi

Kit (Qiagen, catalog no. 13343). DNA from each line was sent to the University of California, Berkeley (UC Berkeley) QB3 Genomics for whole-genome sequencing. Variant calling analysis was completed by Gladstone Institutes Bioinformatics Core, resulting in VCF compressed files for each cell line with variants that passed quality check.

To determine phasing of mutations and common variants, genomic DNA from E396K-P1 and E396K-P2 was extracted using the DNeasy Blood and Tissue Kit (Qiagen, catalog no. 69506). A PCR reaction was performed with primers flanking the E396K mutation and heterozygous variant rs2976439. PCR amplicons were run on a 1% agarose gel, and a band of the expected size was extracted using the QIAquick Gel Extraction Kit (Qiagen, catalog no.28704). The resulting amplicon was cloned into competent bacteria using the TOPO-TA Cloning Kit (Thermo Fisher Scientific, catalog no.450641). The transformation reaction was plated on LB agar with ampicillin and supplied with 120 μL X-Gal (20 mg/mL), Xgal (APExBIO, catalog no. A2539), and 40 μL 100 mM isopropyl β-D-1-thiogalactopyranoside (Gold Bio, catalog no. I2481C). After overnight incubation, white colonies were picked and grown in 5 mL LB Broth supplemented with ampicillin (Fisher BioReagents, catalog no. BP176025). The QIAprep Spin Miniprep Kit (Qiagen, catalog no. 27106) was used to isolate plasmid DNA with amplicon inserts and sent for sequencing. Sequencing of multiple clones confirmed that the E396K mutation was linked to the variant allele of rs2976439 in both cell lines.

The phasing of the N98S-P2 line was identified during the characterization of the N98S-P2-frameshift line. A TaqMan SNP genotyping assay targeting the rs79736124 SNP in the 3′ UTR of *NEFL* (Thermo Fisher, catalog no. C_105316276_10) was used to measure allele-specific mRNA expression. We observed a loss of reference allele expression in the clone with N98S-specific frameshift, allowing us to confirm that the N98S mutation in the N98S-P2 patient line is in phase with the reference allele for rs79736124.

For both N98S-P2 and E396K-P2, the phasing of more distal SNPs was inferred from population-specific haplotype frequencies using the LDHap tool on the NIH LDLink website (https://ldlink.nih.gov/?tab=ldhap).[66] Due to strong linkage disequilibrium across the locus, we inferred that the mutation in N98S-P2 was in phase with the reference allele, and the mutations in E396K-P1 and E396K-P2 were in phase with the alternate allele for each variant shown in Figure 1. All experimental results were consistent with these inferred linkage predictions.

iPSC generation and maintenance

WTC is an extensively characterized and utilized iPSC line from a healthy individual[67] that is the parental line for the Allen Institute Cell Collection (http://www.allencell.org/). Generation of iPSCs from peripheral blood mononuclear cells from the N98S-P2 patient was reported previously.[27] Fibroblasts from the E396K-P1 and E396K-P2 patients were reprogrammed into iPSCs using Stem Cell Technology's ReproRNA-OKSGM kit (catalog no. 05930). Colonies with characteristic iPSC morphology were manually picked and expanded as independent clones sent for karyotypic analysis. Derivation and use of human iPSCs, including whole-genome sequencing, were approved and performed in accordance with the rules and regulations of the UC San Francisco Committee on Human Research (study no. 10-02521). All subjects provided informed consent prior to participation.

Established iPSCs were cultured on Matrigel (Corning, catalog no. 356231)-coated plates at 37°C, 5% CO_2, and 85% humidity. iPSCs were fed mTeSR Plus (Stem Cell Technologies, catalog no. 100–0276) every other day and passaged every 3–4 days with Accutase (Stem Cell Technologies, catalog no. 07920) or ReLeSR (Stem Cell Technologies, catalog no. 100–0483). After passaging, iPSCs were plated into mTeSR Plus with 10 μM Y-27632 (SelleckChem, catalog no. S1049). For analysis of pluripotency markers, iPSCs were lifted and singularized using Accutase. For analysis of extracellular markers, cells were incubated with conjugated antibodies for 30 min on ice and protected from light. For analysis of intracellular markers, cells were fixed, permeabilized, and stained using the Fix and Perm Kit (Thermo Fisher Scientific) according to the manufacturer's instructions. (See Table S3 for details of antibodies used.) Cells were analyzed using BD FACS Aria. After flow cytometry was performed, analysis was performed using FlowJo software.

hNIL engineering

Patient-derived N98S-P2, E396K-P1, and E396K-P2 iPSC lines were engineered to contain a doxycycline inducible vector expressing human transcription factors NGN2, ISL1, and LHX3 (hNIL) in the CLYBL safe harbor locus as previously described.[10,38] Briefly, after nucleofection of the hNIL vector and CLYBL-targeting transcription activator-like effector nuclease plasmids, red fluorescent iPSC colonies were isolated and genotyped via junction PCR to verify hNIL integration in the CLYBL locus, and via copy-number ddPCR. The ddPCR assay used a custom TRE3G or neomycin copy-number assay with RPP30 copy-number assay (Bio-Rad, catalog no. dHsaCP1000485) as a two-copy control. Clones with homozygous hNIL integration were selected and an mCherry-neomycin resistance selection cassette was removed via nucleofection of a Cre recombinase plasmid (Addgene plasmid, catalog no. 11543). Post-transfection, non-fluorescent clones were further genotyped via junction PCR (Table S4) and copy-number ddPCR using a custom TRE3G copy-number assay and an RPP30 ddPCR copy-number assay (Table S5).

RNP transfections

To prepare the RNP for single-guide editing, 240 pmol gRNA (Synthego) was mixed with 120 pmol Hi-Fi SpCas9 protein (HiFiCas9, Macrolab) and incubated for 30 min at room temperature. For dual-gRNA excision, 120 pmol of each gRNA (Synthego) was complexed separately with 60 pmol HiFiCas9 protein and incubated for 30 min at room temperature. The RNP complexes were combined immediately prior to transfection. All gRNA target sequences are listed in Table S6. After dissociation with Accutase, 3.0×10^5 cells

were resuspended in P3 buffer containing the RNP complex(es). Optionally, 50 pmol ssODN was added to the cell suspension at this step. All ssODN sequences are listed in Table S3 iPSCs, and day 3 i^{3}LMNs were nucleofected using the P3 Primary Cell 4D-Nucleofector X Kit S (Lonza, catalog no. V4XP-3032) with pulse code DS138. After nucleofection, cells were incubated for 5 min at room temperature. iPSCs were plated into mTeSR Plus with 10 μM Y-27632, while i^{3}LMNs were plated into the standard neural induction media used for differentiation. Genomic DNA was extracted from edited and unedited cells 3–4 days post-nucleofection using the DNeasy Blood and Tissue Kit or Quick Extract DNA Extract Solution (Lucigen, catalog no. QE9050).

Isolation of clonal edited lines

The N98S-P2-fs and N98S-P2-cor iPSC lines were generated and maintained as previously described,[10,68] with minor changes to the maintenance conditions (i.e., mTeSR Plus was used in place of Stemfit). To generate excision and inversion iPSC lines, N98S-P2 and E396K-P2 iPSCs were nucleofected with paired RNP complexes as above. After nucleofection, the cells were seeded in a 6-well plate with serial dilution to obtain a range of seeding densities across the plate. Cells were maintained for 7 days or until individual iPSC colonies were large enough for manual clone picking. gDNA of the pooled edited cells was collected, and editing frequencies were assessed via ddPCR. A total of 20–30 individual colonies for each experiment were picked and transferred into a 48-well plate coated with Matrigel (Corning 356231). Once confluent, they were further expanded and gDNA was harvested using Quick Extract DNA Extract Solution (Lucigen, catalog no. QE9050). In general, 8–10 of the selected clones contained the desired excision or inversion event. The genotypes of the iPSC clones were assessed via ddPCR assays for excision and inversion at the target allele (Tables S7–S9). Sanger sequencing of the non-target allele near each gRNA target site was performed to rule out indels and confirmed editing at the desired allele (sequencing primers listed in Table S10). Representative clones containing each desired editing outcome from N98S-P2 and separately from E396K-P2 were expanded for cryopreservation and differentiation.

i^{3}LMN differentiation

Patient-derived iPSC lines engineered with the hNIL transgene were differentiated into i^{3}LMNs as described.[10,38] For gene expression assays, 1.25 $\times$ 10^5 cells/cm^2 were seeded onto poly-D-lysine (Sigma, catalog no. P7405) and laminin-coated 6-well plates on day 3. For imaging assays, approximately 5–8 $\times$ 10^4 cells/cm^2 were seeded into similarly coated 96-well plates on day 3.

In vitro cutting assay

RNP was complexed by addition of 1 mL of 10 μM Cas9 with 3 mL of 10 μM gRNA in 3 mL NebBuffer r3.1 (NEB, catalog no. B6003S) and 20 mL DNAse/RNase free water. This was incubated at room temperature for 10 min. Purified PCR amplicon (3 mL of 125 ng/mL) was added to the reaction and vortexed lightly, followed by a 15-min incubation at 37°C. Proteinase K (1 mL) was added, and the reaction was incubated at room temperature for 10 min. The reaction was run via gel electrophoresis on a 2% agarose gel and stained with SYBR Safe for imaging.

Measuring indels and excision junctions with amplicon sequencing

To measure single-guide editing efficiency and specificity via NGS, the targeted regions were amplified with primers containing Illumina adapter sequences (Table S10) using Q5 High-Fidelity 2× Master Mix (NEB, catalog no. M0492L). PCR amplicons were isolated using PCR cleanup beads from the UC Berkeley DNA Sequencing Facility and eluted in diethylpyrocarbonate-treated water. Samples were submitted to Quintara Biosciences for Illumina-based NGS. FASTQ files were processed with Geneious Prime, and the editing outcomes were analyzed using the allele-specific CRISPResso2 pipeline.[43] For Sanger sequencing, PCR amplicons were submitted to Quintara Biosciences, and sequencing traces were analyzed using Synthego's Inference of CRISPR Edits tool.[69] Excision junction sequencing was performed as described in alternative protocol 1 of Vasquez et al.[70] Excision junctions were amplified with Q5 High-Fidelity 2× Master Mix (NEB, catalog no. M0492L) using primers at a 0.3-μM final concentration (Table S10), with 2 μL genomic DNA as template in a 25-μL reaction. A second round of PCR (10 cycles) added indexes and Illumina P5/P7 sequences. Samples were pooled, purified by gel extraction, quantified by Qubit (double-strandedDNA high-sensitivity assay kit), and sequenced on an Illumina NextSeq 2000.

Measuring excisions and inversions

Excision and inversion frequency were measured via ddPCR according to the methods previously described using the Bio-Rad QX200 System and QuantaSoft software.[27] Briefly, for excision assays, forward and reverse primers flanked the two gRNA target sites. An FAM (single isomer derivative of fluorescein)-labeled probe (IDT) was designed to bind within the PCR amplicon but outside the predicted excision. For inversion assays, the same FAM probe and its adjacent primer were used with a primer oriented between the two gRNA cut sites such that amplification could occur only in the presence of inversion. For loss-of-signal assay an internal primer and probe assay was designed to exon 4. Each excision/inversion reaction included a hexachlorofluorescein-labeled RPP30 copy-number assay (Bio-Rad, catalog no. dHsaCP2500350) as an internal reference for normalization. All primer and probe assay combinations are listed in Table S7, with the sequences in Table S9.

ddPCR specificity assay for excision and inversion

A multi-step PCR-ddPCR assay was designed to quantify the percentage of excision or inversion edits that occurred on each allele, as illustrated in Figure S17. Primers were designed to amplify the excised or inverted DNA, including the SNP rs2979685, which is upstream of the *NEFL* gene. PCR amplicons were run on a 1% agarose gel, and a band of the expected size was extracted using QIAquick Gel Extraction Kit (Qiagen, catalog no. 28706). Purified amplicon DNA (0.01 pg) was used in a 25-μL ddPCR reaction containing a

TaqMan SNP genotyping assay targeting rs2979685 (Thermo Fisher, assay ID: C__11857476_30). Fractional abundance of the FAM vs. VIC (asymmetric xanthene dye) signal determined the percentage of target allele versus non-target allele excised or inverted. All primer and probe combinations are listed in Table S8 with the sequences in Table S9.

dPCR assay for combined excision frequency and specificity

A four-color assay using the QIAcuity One 5plex Device (Qiagen, catalog no. 911021) was used to measure excision frequency and specificity by single-step digital PCR as illustrated in Figure S18. Genomic DNA (25–150 ng) was used in a 12-μL reaction containing a TaqMan SNP genotyping assay for rs2979685 (FAM/VIC), a custom-designed primer and ROX-labeled (carboxy-X-rhodamine) probe mix to detect excision events, and Cy5-labeled RPP30 copy-number control (Bio-Rad, catalog no. dHsaCNS753942929). The reactions were loaded into an 8.5-K partition 96-well plate and processed in a QIAcuity One instrument. The QIAcuity Software Suite was used for data analysis as follows and illustrated in Figure S18. Excision frequency was calculated as the ratio of ROX/Cy5$^+$ partitions. Specificity was calculated by the number of partitions positive for both excision (ROX) and the target allele (FAM or VIC) divided by the total number of double-positive partitions (ROX + FAM or ROX + VIC). Ambiguous partitions that were triple-positive (ROX + FAM + VIC) were excluded from the calculation but consistently represented less than 10% of all ROX$^+$ partitions.

Off-target analysis

Sanger sequencing was used to determine whether indels were produced at predicted off-target sites in the CRISPR-engineered cell lines as previously described. In brief, targeted amplification and sequencing was performed for all genomic sites with a >0.25 cutting frequency determination score.[71] This analysis was performed for unedited N98S-P2 and E396K-P2, N98S-P2-fs, N98S-P2-cor, N98S-P2-ex, N98S-P2-inv, E369K-P2-ex, and E369K-P2-inv. All sequences that deviated from reference were present in both the edited and unedited controls, consistent with preexisting variants in the parental lines.

Gene expression analysis by ddPCR

RNA was extracted from i^{3}LMNs using the Quick-RNA Miniprep Kit (Zymo Research, catalog no. R1055), according to the manufacturer's instructions. RNA concentrations were quantified using the NanoDrop spectrophotometer. Reverse transcription was performed using the iScript cDNA Synthesis Kit (Bio-Rad, catalog no. 1708891). To determine the total mRNA expression, i^{3}LMN cDNA (0.5 ng) was amplified in a 25-μL ddPCR reaction using commercially available FAM-labeled TaqMan gene expression assays for *NEFL* (Thermo Fisher, catalog no. Hs00196245_m1) or *NEFM* (Thermo Fisher, catalog no. Hs00193572_m1) in combination with HEX-labeled *GAPDH* gene expression assay (Bio-Rad, catalog no. 10031255) as an internal control for normalization. To determine allele-specific *NEFL* mRNA expression, i^{3}LMN cDNA (0.5 ng) was amplified in a 25-μL ddPCR reaction with TaqMan SNP genotyping assay targeting rs79736124 SNP in the 3′ UTR of *NEFL* (Thermo Fisher, catalog no. C_105316276_10). All ddPCR reactions were analyzed using the BioRad QX200 System and QuantaSoft software. For total mRNA expression of *NEFL* and *NEFM*, the ratio of FAM$^+$ to HEX$^+$ droplets was calculated to normalize to *GAPDH*. Fractional abundance of FAM$^+$ vs. VIC$^+$ droplets was used to quantify allele-specific *NEFL* expression.

Immunofluorescent staining

Immunofluorescent staining was conducted as previously described,[10] with the following modifications: i^{3}LMNs were cultured in clear-bottom imaging 96-well plates and fixed with an equivalent volume of 4% paraformaldehyde (PFA; Electron Microscopy Sciences, catalog no. 50-980-487) in PBS added directly to the cell culture media. To minimize lifting of the neuronal culture, the plate was held upright so that the media pooled at the bottom of the well; using a multichannel pipette, media was slowly skimmed off the top of the pool and added slowly to the edge of the pool. Excessive drying of the wells was avoided. Cells were incubated in PFA at room temperature for 20 min, then washed with PBS with 0.1% Triton X-100 (PBS-T, Sigma) once quickly, followed by two 15-min washes. The cells were blocked and permeabilized for 1 h at room temperature with 5% normal goat serum (Thermo Fisher Scientific, catalog no. PCN5000) in PBS-T. The cells were incubated in primary antibodies at appropriate dilutions (Table S3) in 3% normal goat serum in PBS-T at 4°C overnight. The following day, the cells were washed with PBS at room temperature once quickly, followed by three 10-min washes. The cells were incubated in fluorescent conjugated secondary antibodies diluted at 1:500 in 3% normal goat serum in PBS-T for 1 h at room temperature. The cells were washed with PBS once quickly, followed by three 10-min washes. The first 10-min wash contained DAPI dye (Invitrogen, catalog no. D1306).

Microscopy and imaging analysis

Images were taken on a Keyence BZ-9000 fluorescence microscope with a 40× objective or the automated Cell Insight CX7 LZR Pro HCS Platform at 20× (Thermo Fisher Scientific). The CX7 microscope has a field size of 444.54 × 444.54 μm, and the camera acquisition mode is 1,104 × 1,104 (2 × 2 binning). To maximize the consistency and reproducibility of this analysis, all neurons for each experiment were fixed and stained simultaneously using a single aliquot and dilution of each antibody prior to imaging. Five (Keyence) or 15 (CX7) images were acquired per well. All images for each experiment were acquired in a single session with constant illumination and exposure parameters.

The cell bodies and neurites of i^{3}LMNs were segmented using CellProfiler pipelines as illustrated in Figures S9 and S10. To measure fluorescence intensity in the cell body, the CellProfiler pipeline defined the nuclei of neurons with HB9 (original) or with DAPI (modified). The diameter of each nucleus was expanded by 10 pixels to encompass the entirety of the cell body. After thresholding, this inferred cell body mask was overlaid onto the NfL channel. Objects were filtered by compactness and shape to eliminate objects that are

not cell bodies and mean intensity was measured. To measure fluorescence intensity in the neurites, the pipeline similarly identified DAPI+ nuclei and expanded the objects by 15 pixels to encompass the cell body and axon hillock. NfL and β3-tubulin channels were thresholded, and an inverted mask of the inferred cell bodies was overlaid on the thresholded β3-tubulin channel to produce a neurite mask. The neurite mask was overlaid on the thresholded NfL channel, and total fluorescence intensity was measured and divided by the total area of the neurite mask to normalize for neurite density.

Multi-electrode array

N98S-P2 and its associated frameshift, corrected, excision, and inversion lines, along with E396K and its associated excision and inversion lines were differentiated into i^{3}LMNs and seeded in 24-well CytoView MEA plates (Axion Biosystems, catalog no. M384-tMEA-24W) on day 3 as previously described.[10] BrainPhys (STEMCELL Technologies, catalog no. 05791) was used as basal media starting on day 7. Spontaneous neural activity was measured for 15 min on days 7, 14, 21, and 28 using the Axion Biosystem Maestro Edge MEA systems and analyzed as previously described.[10]

Statistical analysis

All statistical analyses were performed using GraphPad Prism (version 10.4.1). Pairwise comparisons were done by unpaired two-tailed *t* tests. Multiple comparisons with a single variable were performed by one-way ANOVA followed by the recommended post-test as follows: Dunnett's test for comparisons of multiple treatments to a control, Tukey's test for comparisons of each treatment to every other treatment, and Šídák's test for comparisons of pre-selected pairs of treatments. Comparisons of multiple variables were performed by two-way ANOVA followed by Šídák's test for selected variables.

Computational methods

The frequency of heterozygotes in the 1000 Genomes Phase 3 dataset was used to select guide-pair candidates.[40] Common variants (allele frequencies between 0.1 and 0.9) were considered in the 50-kb regions on either side of *NEFL*. This resulted in a total of 151 SNPs: 66 upstream and 85 downstream of the *NEFL* gene body. Then, for each flanking pair of SNPs, the number of individuals out of the total of 2,548 that were heterozygous for both SNPs was identified. To explore the linkage disequilibrium patterns in the region, these 151 SNs, plus 3 common (0.1 < allele frequency <0.9) SNPs from within the *NEFL* gene, were computed across all 1000 Genomes populations and visualized using the LDMatrix tool on the NIH LDLink website (https://ldlink.nih.gov/?tab=ldmatrix).[66]

To identify a small collection of SNP pairs that enable editing a large number of diverse individuals, a greedy algorithm was applied to select SNP pairs. At each iteration, the pair of SNPs was chosen that was heterozygous in the largest number of individuals not covered by a previously selected pair. Those individuals were then removed from consideration, and the process was repeated until an acceptable proportion of individuals was covered or a maximum number of SNP pairs was reached.

1000 Genomes variants (in VCF format) are available at (ftp.1000genomes.ebi.ac.uk/vol1/ftp/data_collections/1000_genomes_project/release/20190312_biallelic_SNP_and_INDEL/).[40]

DATA AND CODE AVAILABILITY

All experimental data generated or analyzed during this study are available from the corresponding authors upon reasonable request.

ACKNOWLEDGMENTS

We thank Michael Shy for providing primary dermal fibroblasts from patients with E396K mutations. We thank Wendy Runyon, Hana Ghanim, and Angela Liu for technical assistance with iPSC reprogramming and integration of hNIL transgenes. We thank Anke Meyer-Franke and the Gladstone Assay Development and Drug Discovery Core for advice and technical assistance with automated microscopy. We thank Jane Srivastava and the Gladstone Flow Cytometry Core for analysis of pluripotency markers. We thank the Gladstone Stem Cell Core, Gladstone Bioinformatics Core, Gladstone Histology and Light Microscopy Core, and the UCSF Center for Advanced Technology (CAT) for their resources and services. We thank Bria Macklin, Zachary Nevin, Gokul Ramadoss, and Françoise Chanut for helpful discussions and review of the manuscript. L.M.J. and B.R.C. received funding from the Charcot-Marie-Tooth Association and NIH grants U01-ES032673 and R01-NS119678. J.A.C. received funding from NIH grant R35GM127087. G.D.R. was supported by NIH grant F31AG090013-01. A.A. was supported by NIH grant T32GM007347 and AHA fellowship 20PRE35080073. Q.T.C. was funded by CIRM Regenerative Medicine Research Training Program grant no. EDUC4-12766. Sequencing performed at the UCSF CAT was supported by UCSF Program for Breakthrough Biomedical Research, Research Resource Program Institutional Matching Instrumentation Award, and NIH 1S10OD028511-01 grants.

AUTHOR CONTRIBUTIONS

L.M.J. and B.R.C. conceived the project. P.H.D., B.M.J.S., C.B.E.M., H.L.W., and L.M.J. designed the experiments. P.H.D., C.M.F., C.B.E.M., B.M.J.S., and H.L.W. completed the cell line generation and characterization. P.H.D., B.M.J.S., C.M.F., C.B.E.M., and Q.T.C. performed the gene editing experiments and assays. P.H.D. and B.M.J.S. performed the gene expression analysis. P.H.D., C.B.E.M., and B.M.J.S. performed the neuron differentiations and neuron imaging. P.H.D. and C.B.E.M. performed the multielectrode array analysis. C.B.E.M., P.H.D., and D.S. designed and executed the CellProfiler analysis pipelines. A.A., E.N.G., G.D.R., and J.A.C. performed the computational analyses. P.H.D., B.M.J.S., and L.M.J. prepared the figures and wrote the manuscript, with assistance from all authors.

DECLARATION OF INTERESTS

B.R.C. is a founder of Tenaya Therapeutics (https://www.tenayatherapeutics.com/), a company focused on finding treatments for heart failure, including genetic cardiomyopathies, and holds equity in Tenaya. L.M.J. has received royalty payments for cell lines licensed to Tenaya Therapeutics related to genetic cardiomyopathy and heart failure research.

SUPPLEMENTAL INFORMATION

Supplemental information can be found online at https://doi.org/10.1016/j.ymthe.2025.11.026.

REFERENCES

1. Kan, M.J., and Doudna, J.A. (2022). Treatment of genetic diseases with CRISPR genome editing. JAMA *328*, 980–981. https://doi.org/10.1001/jama.2022.13468.
2. Doudna, J.A., and Charpentier, E. (2014). Genome editing. The new frontier of genome engineering with CRISPR-Cas9. Science *346*, 1258096. https://doi.org/10.1126/science.1258096.
3. Wu, Y., Zeng, J., Roscoe, B.P., Liu, P., Yao, Q., Lazzarotto, C.R., Clement, K., Cole, M.A., Luk, K., Baricordi, C., et al. (2019). Highly efficient therapeutic gene editing of

human hematopoietic stem cells. Nat. Med. *25*, 776–783. https://doi.org/10.1038/s41591-019-0401-y.

4. Philippidis, A. (2024). CASGEVY makes history as FDA approves first crispr/cas9 genome edited therapy. Hum. Gene Ther. *35*, 1–4. https://doi.org/10.1089/hum.2023.29263.bfs.

5. Intellia Therapeutics (2023). Study to Evaluate Safety, Tolerability, Pharmacokinetics, and Pharmacodynamics of NTLA-2001 in Patients With Hereditary Transthyretin Amyloidosis With Polyneuropathy (ATTRv-PN) and Patients With Transthyretin Amyloidosis-Related Cardiomyopathy (ATTR-CM). https://clinicaltrials.gov/study/NCT04601051.

6. Intellia Therapeutics (2024). MAGNITUDE: a Phase 3 Study of NTLA-2001 in Participants with Transthyretin Amyloidosis with Cardiomyopathy (ATTR-CM). https://clinicaltrials.gov/study/NCT06128629.

7. Intellia Therapeutics (2024). NTLA-2002 in Adults With Hereditary Angioedema (HAE) (NTLA-2002). https://clinicaltrials.gov/study/NCT05120830.

8. Verve Therapeutics, Inc. (2025). Open-label, Phase 1b, Single-ascending Dose and Optional re Dosing Study to Evaluate the Safety of VERVE-101 Administered to Patients With Heterozygous Familial Hypercholesterolemia, Atherosclerotic Cardiovascular Disease, and Uncontrolled Hypercholesterolemia. https://clinicaltrials.gov/study/NCT05398029.

9. György, B., Nist-Lund, C., Pan, B., Asai, Y., Karavitaki, K.D., Kleinstiver, B.P., Garcia, S.P., Zaborowski, M.P., Solanes, P., Spataro, S., et al. (2019). Allele-specific gene editing prevents deafness in a model of dominant progressive hearing loss. Nat. Med. *25*, 1123–1130. https://doi.org/10.1038/s41591-019-0500-9.

10. Feliciano, C.M., Wu, K., Watry, H.L., Marley, C.B.E., Ramadoss, G.N., Ghanim, H.Y., Liu, A.Z., Zholudeva, L.V., McDevitt, T.C., Saporta, M.A., et al. (2021). Allele-Specific Gene Editing Rescues Pathology in a Human Model of Charcot-Marie-Tooth Disease Type 2E. Front. Cell Dev. Biol. *9*, 723023. https://doi.org/10.3389/fcell.2021.723023.

11. Liu, X., Qiao, J., Jia, R., Zhang, F., Meng, X., Li, Y., and Yang, L. (2023). Allele-specific gene-editing approach for vision loss restoration in RHO-associated retinitis pigmentosa. eLife *12*, e84065. https://doi.org/10.7554/eLife.84065.

12. Bolduc, V., Sizov, K., Brull, A., Esposito, E., Chen, G.S., Uapinyoying, P., Sarathy, A., Johnson, K.R., and Bönnemann, C.G. (2024). Allele-specific CRISPR-Cas9 editing inactivates a single nucleotide variant associated with collagen VI muscular dystrophy. Mol. Ther. Nucleic Acids *35*, 102269. https://doi.org/10.1016/j.omtn.2024.102269.

13. Jordanova, A., De Jonghe, P., Boerkoel, C.F., Takashima, H., De Vriendt, E., Ceuterick, C., Martin, J.J., Butler, I.J., Mancias, P., Papasozomenos, S.C., et al. (2003). Mutations in the neurofilament light chain gene (NEFL) cause early onset severe Charcot-Marie-Tooth disease. Brain. *126*, 590–597. https://doi.org/10.1093/brain/awg059.

14. Pisciotta, C., Bai, Y., Brennan, K.M., Wu, X., Grider, T., Feely, S., Wang, S., Moore, S., Siskind, C., Gonzalez, M., et al. (2015). Reduced neurofilament expression in cutaneous nerve fibers of patients with CMT2E. Neurology *85*, 228–234. https://doi.org/10.1212/WNL.0000000000001773.

15. Adebola, A.A., Di Castri, T., He, C.-Z., Salvatierra, L.A., Zhao, J., Brown, K., Lin, C.-S., Worman, H.J., and Liem, R.K.H. (2015). Neurofilament light polypeptide gene N98S mutation in mice leads to neurofilament network abnormalities and a Charcot-Marie-Tooth Type 2E phenotype. Hum. Mol. Genet. *24*, 2163–2174. https://doi.org/10.1093/hmg/ddu736.

16. Lancaster, E., Li, J., Hanania, T., Liem, R., Scheideler, M.A., and Scherer, S.S. (2018). Myelinated axons fail to develop properly in a genetically authentic mouse model of Charcot-Marie-Tooth disease type 2E. Exp. Neurol. *308*, 13–25. https://doi.org/10.1016/j.expneurol.2018.06.010.

17. Pérez-López, D.O., Shively, A.A., Llorente Torres, F.J., Abu-Salah, M.T., Garcia, M.L., Arnold, W.D., Lorson, M.A., and Lorson, C. (2025). Novel neurofilament light (Nefl) E397K mouse models of Charcot-Marie-tooth type 2E (CMT2E) present early and chronic axonal neuropathy. Hum. Mol. Genet. *34*, 1541–1552. https://doi.org/10.1093/hmg/ddaf116.

18. Yuan, A., Rao, M.V., and Nixon, R.A. (2017). Neurofilaments and neurofilament proteins in health and disease. Cold Spring Harb. Perspect. Biol. *9*, a018309. https://doi.org/10.1101/cshperspect.a018309.

19. Zhao, J., Brown, K., and Liem, R.K.H. (2017). Abnormal neurofilament inclusions and segregations in dorsal root ganglia of a Charcot-Marie-Tooth type 2E mouse model. PLoS ONE *12*, e0180038. https://doi.org/10.1371/journal.pone.0180038.

20. Perez-Olle, R., Jones, S.T., and Liem, R.K.H. (2004). Phenotypic analysis of neurofilament light gene mutations linked to Charcot-Marie-Tooth disease in cell culture models. Hum. Mol. Genet. *13*, 2207–2220. https://doi.org/10.1093/hmg/ddh236.

21. Pérez-Ollé, R., López-Toledano, M.A., Goryunov, D., Cabrera-Poch, N., Stefanis, L., Brown, K., and Liem, R.K.H. (2005). Mutations in the neurofilament light gene linked to Charcot-Marie-Tooth disease cause defects in transport. J. Neurochem. *93*, 861–874. https://doi.org/10.1111/j.1471-4159.2005.03095.x.

22. Stone, E.J., Uchida, A., and Brown, A. (2019). Charcot-Marie-Tooth disease Type 2E/1F mutant neurofilament proteins assemble into neurofilaments. Cytoskeleton (Hoboken) *76*, 423–439. https://doi.org/10.1002/cm.21566.

23. Fu, J., and Yuan, Y. (2018). A novel homozygous nonsense mutation in NEFL causes autosomal recessive Charcot-Marie-Tooth disease. Neuromuscul. Disord. *28*, 44–47. https://doi.org/10.1016/j.nmd.2017.09.018.

24. Yum, S.W., Zhang, J., Mo, K., Li, J., and Scherer, S.S. (2009). A novel recessive Nefl mutation causes a severe, early-onset axonal neuropathy. Ann. Neurol. *66*, 759–770. https://doi.org/10.1002/ana.21728.

25. Saporta, M.A., Dang, V., Volfson, D., Zou, B., Xie, X.S., Adebola, A., Liem, R.K., Shy, M., and Dimos, J.T. (2015). Axonal Charcot-Marie-Tooth disease patient-derived motor neurons demonstrate disease-specific phenotypes including abnormal electrophysiological properties. Exp. Neurol. *263*, 190–199. https://doi.org/10.1016/j.expneurol.2014.10.005.

26. Maciel, R., Correa, R., Bosso Taniguchi, J., Prufer Araujo, I., and Saporta, M.A. (2020). Human tridimensional neuronal cultures for phenotypic drug screening in inherited peripheral neuropathies. Clin. Pharmacol. Ther. *107*, 1231–1239. https://doi.org/10.1002/cpt.1718.

27. Watry, H.L., Feliciano, C.M., Gjoni, K., Takahashi, G., Miyaoka, Y., Conklin, B.R., and Judge, L.M. (2020). Rapid, precise quantification of large DNA excisions and inversions by ddPCR. Sci. Rep. *10*, 14896. https://doi.org/10.1038/s41598-020-71742-z.

28. Ruan, G.-X., Barry, E., Yu, D., Lukason, M., Cheng, S.H., and Scaria, A. (2017). CRISPR/Cas9-Mediated Genome Editing as a Therapeutic Approach for Leber Congenital Amaurosis 10. Mol. Ther. *25*, 331–341. https://doi.org/10.1016/j.ymthe.2016.12.006.

29. Maeder, M.L., Stefanidakis, M., Wilson, C.J., Baral, R., Barrera, L.A., Bounoutas, G.S., Bumcrot, D., Chao, H., Ciulla, D.M., DaSilva, J.A., et al. (2019). Development of a gene-editing approach to restore vision loss in Leber congenital amaurosis type 10. Nat. Med. *25*, 229–233. https://doi.org/10.1038/s41591-018-0327-9.

30. Kaminski, R., Chen, Y., Fischer, T., Tedaldi, E., Napoli, A., Zhang, Y., Karn, J., Hu, W., and Khalili, K. (2016). Elimination of HIV-1 Genomes from Human T-lymphoid Cells by CRISPR/Cas9 Gene Editing. Sci. Rep. *6*, 22555. https://doi.org/10.1038/srep22555.

31. Excision BioTherapeutics (2024). Study of EBT-101 in Aviremic HIV-1 Infected Adults on Stable ART. Study of EBT-101 in Aviremic HIV-1 Infected Adults on Stable ART. https://clinicaltrials.gov/study/NCT05144386.

32. Editas Medicine, Inc. (2022). Single Ascending Dose Study in Participants With LCA10. Open-Label, Single Ascending Dose Study to Evaluate the Safety, Tolerability, and Efficacy of EDIT-101 in Adult and Pediatric Participants With Leber Congenital Amaurosis Type 10 (LCA10), With Centrosomal Protein 290 (CEP290)-Related Retinal Degeneration Caused by a Compound Heterozygous or Homozygous Mutation Involving c.2991+1655A>G in Intron 26 (IVS26) of the CEP290 Gene ("LCA10-IVS26"). https://clinicaltrials.gov/study/NCT03872479.

33. Sachdev, A., Gill, K., Sckaff, M., Birk, A.M., Aladesuyi Arogundade, O., Brown, K.A., Chouhan, R.S., Issagholian-Lewin, P.O., Patel, E., Watry, H.L., et al. (2024). Reversal of C9orf72 mutation-induced transcriptional dysregulation and pathology in cultured human neurons by allele-specific excision. Proc. Natl. Acad. Sci. USA *121*, e2307814121. https://doi.org/10.1073/pnas.2307814121.

34. Shin, J.W., Kim, K.-H., Chao, M.J., Atwal, R.S., Gillis, T., MacDonald, M.E., Gusella, J.F., and Lee, J.-M. (2016). Permanent inactivation of Huntington's disease mutation

by personalized allele-specific CRISPR/Cas9. Hum. Mol. Genet. *25*, 4566–4576. https://doi.org/10.1093/hmg/ddw286.

35. Monteys, A.M., Ebanks, S.A., Keiser, M.S., and Davidson, B.L. (2017). Crispr/cas9 editing of the mutant huntingtin allele in vitro and in vivo. Mol. Ther. *25*, 12–23. https://doi.org/10.1016/j.ymthe.2016.11.010.

36. Christie, K.A., Robertson, L.J., Conway, C., Blighe, K., DeDionisio, L.A., Chao-Shern, C., Kowalczyk, A.M., Marshall, J., Turnbull, D., Nesbit, M.A., and Moore, C.B.T. (2020). Mutation-Independent Allele-Specific Editing by CRISPR-Cas9, a Novel Approach to Treat Autosomal Dominant Disease. Mol. Ther. *28*, 1846–1857. https://doi.org/10.1016/j.ymthe.2020.05.002.

37. Christie, K.A., Courtney, D.G., DeDionisio, L.A., Shern, C.C., De Majumdar, S., Mairs, L.C., Nesbit, M.A., and Moore, C.B.T. (2017). Towards personalised allele-specific CRISPR gene editing to treat autosomal dominant disorders. Sci. Rep. *7*, 16174. https://doi.org/10.1038/s41598-017-16279-4.

38. Fernandopulle, M.S., Prestil, R., Grunseich, C., Wang, C., Gan, L., and Ward, M.E. (2018). Transcription Factor-Mediated Differentiation of Human iPSCs into Neurons. Curr. Protoc. Cell Biol. *79*, e51. https://doi.org/10.1002/cpcb.51.

39. Vakulskas, C.A., Dever, D.P., Rettig, G.R., Turk, R., Jacobi, A.M., Collingwood, M.A., Bode, N.M., McNeill, M.S., Yan, S., Camarena, J., et al. (2018). A high-fidelity Cas9 mutant delivered as a ribonucleoprotein complex enables efficient gene editing in human hematopoietic stem and progenitor cells. Nat. Med. *24*, 1216–1224. https://doi.org/10.1038/s41591-018-0137-0.

40. 1000 Genomes Project Consortium, Auton, A., Brooks, L.D., Durbin, R.M., Garrison, E.P., Kang, H.M., Korbel, J.O., Marchini, J.L., McCarthy, S., McVean, G.A., and Abecasis, G.R. (2015). A global reference for human genetic variation. Nature *526*, 68–74. https://doi.org/10.1038/nature15393.

41. Turchiano, G., Andrieux, G., Klermund, J., Blattner, G., Pennucci, V., El Gaz, M., Monaco, G., Poddar, S., Mussolino, C., Cornu, T.I., et al. (2021). Quantitative evaluation of chromosomal rearrangements in gene-edited human stem cells by CAST-Seq. Cell Stem Cell *28*, 1136–1147.e5. https://doi.org/10.1016/j.stem.2021.02.002.

42. Tsuchida, C.A., Brandes, N., Bueno, R., Trinidad, M., Mazumder, T., Yu, B., Hwang, B., Chang, C., Liu, J., Sun, Y., et al. (2023). Mitigation of chromosome loss in clinical CRISPR-Cas9-engineered T cells. Cell *186*, 4567–4582.e20. https://doi.org/10.1016/j.cell.2023.08.041.

43. Clement, K., Rees, H., Canver, M.C., Gehrke, J.M., Farouni, R., Hsu, J.Y., Cole, M.A., Liu, D.R., Joung, J.K., Bauer, D.E., and Pinello, L. (2019). CRISPResso2 provides accurate and rapid genome editing sequence analysis. Nat. Biotechnol. *37*, 224–226. https://doi.org/10.1038/s41587-019-0032-3.

44. Rabinowitz, R., and Offen, D. (2021). Single-Base Resolution: Increasing the Specificity of the CRISPR-Cas System in Gene Editing. Mol. Ther. *29*, 937–948. https://doi.org/10.1016/j.ymthe.2020.11.009.

45. Carpenter, A.E., Jones, T.R., Lamprecht, M.R., Clarke, C., Kang, I.H., Friman, O., Guertin, D.A., Chang, J.H., Lindquist, R.A., Moffat, J., et al. (2006). CellProfiler: image analysis software for identifying and quantifying cell phenotypes. Genome Biol. *7*, R100. https://doi.org/10.1186/gb-2006-7-10-r100.

46. Chen, F., Pruett-Miller, S.M., Huang, Y., Gjoka, M., Duda, K., Taunton, J., Collingwood, T.N., Frodin, M., and Davis, G.D. (2011). High-frequency genome editing using ssDNA oligonucleotides with zinc-finger nucleases. Nat. Methods *8*, 753–755. https://doi.org/10.1038/nmeth.1653.

47. Wells, A., Heckerman, D., Torkamani, A., Yin, L., Sebat, J., Ren, B., Telenti, A., and di Iulio, J. (2019). Ranking of non-coding pathogenic variants and putative essential regions of the human genome. Nat. Commun. *10*, 5241. https://doi.org/10.1038/s41467-019-13212-3.

48. Stone, E.J., Kolb, S.J., and Brown, A. (2021). A review and analysis of the clinical literature on Charcot-Marie-Tooth disease caused by mutations in neurofilament protein L. Cytoskeleton (Hoboken) *78*, 97–110. https://doi.org/10.1002/cm.21676.

49. Landrum, M.J., Lee, J.M., Riley, G.R., Jang, W., Rubinstein, W.S., Church, D.M., and Maglott, D.R. (2014). ClinVar: public archive of relationships among sequence variation and human phenotype. Nucleic Acids Res. *42*, D980–D985. https://doi.org/10.1093/nar/gkt1113.

50. Landrum, M.J., Chitipiralla, S., Kaur, K., Brown, G., Chen, C., Hart, J., Hoffman, D., Jang, W., Liu, C., Maddipatla, Z., et al. (2025). ClinVar: updates to support classifications of both germline and somatic variants. Nucleic Acids Res. *53*, D1313–D1321. https://doi.org/10.1093/nar/gkae1090.

51. Shin, J.W., Hong, E.P., Park, S.S., Choi, D.E., Seong, I.S., Whittaker, M.N., Kleinstiver, B.P., Chen, R.Z., and Lee, J.-M. (2022). Allele-specific silencing of the gain-of-function mutation in Huntington's disease using CRISPR/Cas9. JCI Insight *7*, e141042. https://doi.org/10.1172/jci.insight.141042.

52. Lek, M., Karczewski, K.J., Minikel, E.V., Samocha, K.E., Banks, E., Fennell, T., O'Donnell-Luria, A.H., Ware, J.S., Hill, A.J., Cummings, B.B., et al. (2016). Analysis of protein-coding genetic variation in 60,706 humans. Nature *536*, 285–291. https://doi.org/10.1038/nature19057.

53. Lindeboom, R.G.H., Vermeulen, M., Lehner, B., and Supek, F. (2019). The impact of nonsense-mediated mRNA decay on genetic disease, gene editing and cancer immunotherapy. Nat. Genet. *51*, 1645–1651. https://doi.org/10.1038/s41588-019-0517-5.

54. Ren, X., Zheng, L., Maliskova, L., Tam, T.W., Sun, Y., Liu, H., Lee, J., Takagi, M.A., Li, B., Ren, B., et al. (2024). CRISPR tiling deletion screens reveal functional enhancers of neuropsychiatric risk genes and allelic compensation effects (ACE) on transcription. Preprint at bioRxiv. https://doi.org/10.1101/2024.10.08.616922.

55. Wu, Y., Liang, D., Wang, Y., Bai, M., Tang, W., Bao, S., Yan, Z., Li, D., and Li, J. (2013). Correction of a genetic disease in mouse via use of CRISPR-Cas9. Cell Stem Cell *13*, 659–662. https://doi.org/10.1016/j.stem.2013.10.016.

56. Kleinstiver, B.P., Prew, M.S., Tsai, S.Q., Nguyen, N.T., Topkar, V.V., Zheng, Z., and Joung, J.K. (2015). Broadening the targeting range of Staphylococcus aureus CRISPR-Cas9 by modifying PAM recognition. Nat. Biotechnol. *33*, 1293–1298. https://doi.org/10.1038/nbt.3404.

57. Kleinstiver, B.P., Sousa, A.A., Walton, R.T., Tak, Y.E., Hsu, J.Y., Clement, K., Welch, M.M., Horng, J.E., Malagon-Lopez, J., Scarfò, I., et al. (2020). Publisher Correction: Engineered CRISPR-Cas12a variants with increased activities and improved targeting ranges for gene, epigenetic and base editing. Nat. Biotechnol. *38*, 901. https://doi.org/10.1038/s41587-020-0587-z.

58. Walton, R.T., Christie, K.A., Whittaker, M.N., and Kleinstiver, B.P. (2020). Unconstrained genome targeting with near-PAMless engineered CRISPR-Cas9 variants. Science *368*, 290–296. https://doi.org/10.1126/science.aba8853.

59. Leckey, C.A., Giovannucci, T.A., Coulton, J.B., He, Y., Sato, C., Ghoshal, N., Vignarajah, T., Jaunmuktane, Z., Barthélemy, N.R., Zetterberg, H., et al. (2025). Stable isotope labeling kinetics of neurofilament light *in vitro* and *in vivo*. Preprint at medRxiv. https://doi.org/10.1101/2025.01.10.24319636.

60. Ramadoss, G.N., Namaganda, S.J., Kumar, M.M., Hamilton, J.R., Sharma, R., Chow, K.G., Workley, L.A., Macklin, B.L., Sun, M., Ha, A.S., et al. (2025). Characterizing and controlling CRISPR repair outcomes in nondividing human cells. Nat. Commun. *16*, 9883.

61. Cullot, G., Boutin, J., Toutain, J., Prat, F., Pennamen, P., Rooryck, C., Teichmann, M., Rousseau, E., Lamrissi-Garcia, I., Guyonnet-Duperat, V., et al. (2019). CRISPR-Cas9 genome editing induces megabase-scale chromosomal truncations. Nat. Commun. *10*, 1136. https://doi.org/10.1038/s41467-019-09006-2.

62. Adikusuma, F., Piltz, S., Corbett, M.A., Turvey, M., McColl, S.R., Helbig, K.J., Beard, M.R., Hughes, J., Pomerantz, R.T., and Thomas, P.Q. (2018). Large deletions induced by Cas9 cleavage. Nature *560*, E8–E9. https://doi.org/10.1038/s41586-018-0380-z.

63. Kosicki, M., Tomberg, K., and Bradley, A. (2018). Repair of double-strand breaks induced by CRISPR-Cas9 leads to large deletions and complex rearrangements. Nat. Biotechnol. *36*, 765–771. https://doi.org/10.1038/nbt.4192.

64. Leibowitz, M.L., Papathanasiou, S., Doerfler, P.A., Blaine, L.J., Sun, L., Yao, Y., Zhang, C.-Z., Weiss, M.J., and Pellman, D. (2021). Chromothripsis as an on-target consequence of CRISPR-Cas9 genome editing. Nat. Genet. *53*, 895–905. https://doi.org/10.1038/s41588-021-00838-7.

65. Saghira, C., Bis, D.M., Stanek, D., Strickland, A., Herrmann, D.N., Reilly, M.M., Scherer, S.S., Shy, M.E.; Inherited Neuropathy Consortium, and Züchner, S. (2018). Variant pathogenicity evaluation in the community-driven Inherited Neuropathy Variant Browser. Hum. Mutat. *39*, 635–642. https://doi.org/10.1002/humu.23412.

66. Machiela, M.J., and Chanock, S.J. (2015). LDlink: a web-based application for exploring population-specific haplotype structure and linking correlated alleles of possible functional variants. Bioinformatics *31*, 3555–3557. https://doi.org/10.1093/bioinformatics/btv402.

67. Kreitzer, F.R., Salomonis, N., Sheehan, A., Huang, M., Park, J.S., Spindler, M.J., Lizarraga, P., Weiss, W.A., So, P.-L., and Conklin, B.R. (2013). A robust method to derive functional neural crest cells from human pluripotent stem cells. Am. J. Stem Cells *2*, 119–131.

68. Miyaoka, Y., Chan, A.H., Judge, L.M., Yoo, J., Huang, M., Nguyen, T.D., Lizarraga, P.P., So, P.-L., and Conklin, B.R. (2014). Isolation of single-base genome-edited human iPS cells without antibiotic selection. Nat. Methods *11*, 291–293. https://doi.org/10.1038/nmeth.2840.

69. Conant, D., Hsiau, T., Rossi, N., Oki, J., Maures, T., Waite, K., Yang, J., Joshi, S., Kelso, R., Holden, K., et al. (2022). Inference of CRISPR Edits from Sanger Trace Data. CRISPR J. *5*, 123–130. https://doi.org/10.1089/crispr.2021.0113.

70. Vasquez, C.A., Cowan, Q.T., and Komor, A.C. (2020). Base Editing in Human Cells to Produce Single-Nucleotide-Variant Clonal Cell Lines. Curr. Protoc. Mol. Biol. *133*, e129. https://doi.org/10.1002/cpmb.129.

71. Doench, J.G., Fusi, N., Sullender, M., Hegde, M., Vaimberg, E.W., Donovan, K.F., Smith, I., Tothova, Z., Wilen, C., Orchard, R., et al. (2016). Optimized sgRNA design to maximize activity and minimize off-target effects of CRISPR-Cas9. Nat. Biotechnol. *34*, 184–191. https://doi.org/10.1038/nbt.3437.

YMTHE, Volume *34*

Supplemental Information

Haplotype editing with CRISPR-Cas9 as a therapeutic approach for dominant-negative missense mutations in *NEFL*

Poorvi H. Dua, Bazilco M.J. Simon, Chiara B.E. Marley, Carissa M. Feliciano, Hannah L. Watry, Quinn T. Cowan, Dylan Steury, Abin Abraham, Erin N. Gilbertson, Grace D. Ramey, John A. Capra, Bruce R. Conklin, and Luke M. Judge

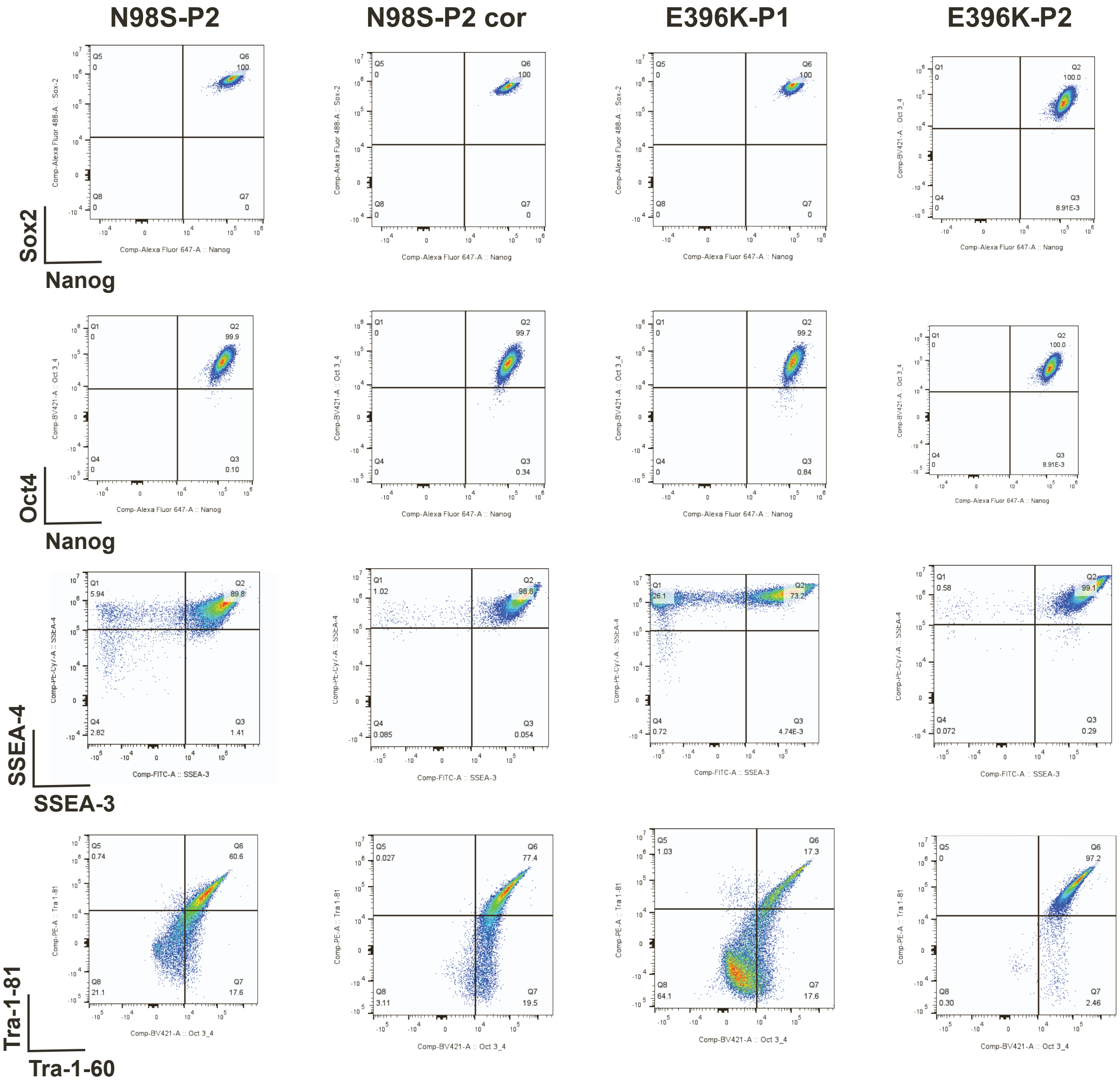

Figure S1: Expression of pluripotency markers in iPSC. Patient-derived iPSC containing the CLYBL-hNIL transgene (N98S-P2, E396K-P1, and E396K-P2) and the isogenic corrected version of N98S-P2 (N98S-P2-cor) were stained with a live/dead marker and antibodies to Sox2, Nanog, Oct 3/4, SSEA-3, SSEA-4, Tra-1-60, and Tra-1-81 (see Table S7 for list of antibodies). Flow cytometry was used to analyze expression of each marker in live cells.

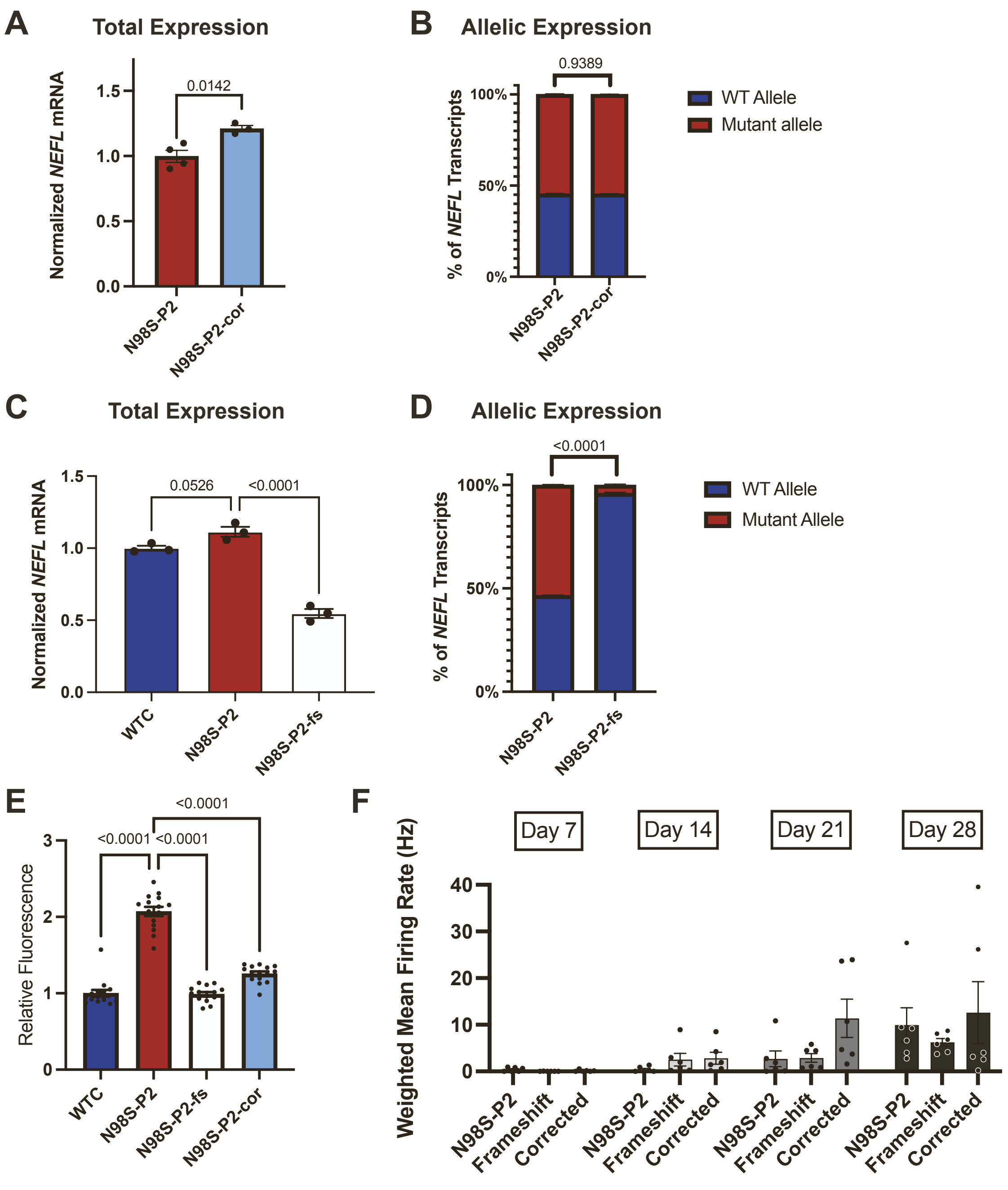

Figure S2: Derivation and characterization of clonal edited iPSC lines derived from N98S-P2 patient line. Transfection of N98S-P2 iPSC with N98S-specific HiFiCas9 RNP produced N98S-P2-fs (contains +1 insertion at the mutation locus). Transfection of N98S-P2 iPSC with N98S-specific HiFiCas9 RNP plus single-strand oligonucleotide donor produced N98S-P2-cor (precise correction of N98S mutation with linked silent mutation to facilitate

genotyping). Clonal iPSC were differentiated into i^{3}LMNs and RNA was extracted on day 7. **(A,C)** Total *NEFL* expression by quantitative RT-ddPCR, normalized to GAPDH. **(B,D)** Relative allelic expression by allele discrimination RT-ddPCR. Bar graphs for A-D represent mean +/- S.E.M. with each data point representing an independent well of i^{3}LMN (n = 3). Statistical comparisons by unpaired two-tailed T-test in (A,B,D) and one-way ANOVA with Dunnett's test for comparison of multiple lines to N98S-P2 in (C). **(E)** Mean NfL fluorescence intensity in the cell bodies of day 7 i^{3}LMNs. Bar graph represents mean +/- S.E.M. of 15 independent wells of i^{3}LMN with each data point representing the mean value from 5 images per well. Statistical comparisons by one-way ANOVA with Dunnett's test for comparison of multiple lines to N98S-P2. **(F)** Day 3 i^{3}LMNs were seeded on multi-electrode array plates to measure spontaneous action potentials in N98S-P2, corrected, and frameshift lines. Spontaneous electrical activity was measured at days 7, 14, 21, and 28. Bar graphs represent mean +/- S.E.M. with each data point representing the mean firing rate from an independent well of i^{3}LMN (n = 6). Statistical comparison by two-way ANOVA demonstrated a statistically significant effect of the differentiation day (p = 0.0002) but no significant difference between cell lines (p = 0.0881).

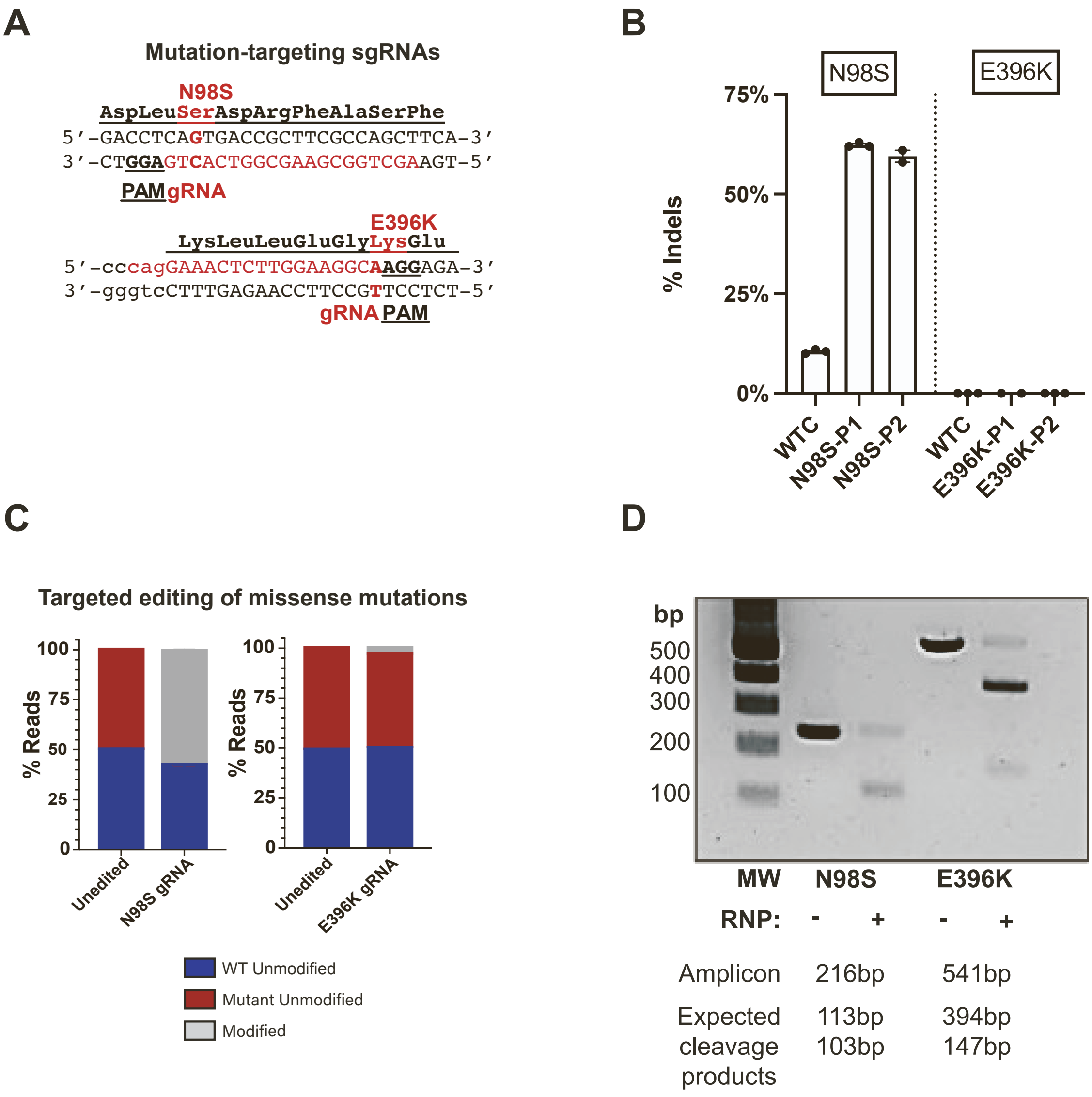

Figure S3: Comparison of the editing and nuclease activity of gRNAs targeting the N98S and E396K mutations. **(A)** Sequences of N98S and E396K mutation-targeting gRNAs (red) with associated PAM sequences (underlined). **(B)** Indel editing efficiency of N98S and E396K mutation-specific gRNAs in multiple patient backgrounds by Sanger sequencing and ICE analysis. Bar graphs represent mean +/- S.E.M with each data pointing indicating an independent transfection (n = 2 – 3). **(C)** NGS amplicon sequencing of N98S and E396K targeted editing in N98S-P2 (left) and E396K-P2 (right) iPSCs. Modified reads contain indels at the target site, WT and mutant unmodified indicate reads without indels on the WT and mutant

alleles, respectively. Bar graphs represent mean +/- S.E.M of independent transfections (n = 3 for N98S, n = 6 for E396K). **(D)** PCR amplicons spanning the N98S or E396K mutations were generated from N98S-P2 and E396K-P2 gDNA, respectively, followed by incubation with corresponding N98S or E396K mutation-specific gRNA and HiFiCas9 RNP. Gel electrophoresis identifies the expected cleavage products. MW = molecular weight DNA ladder.

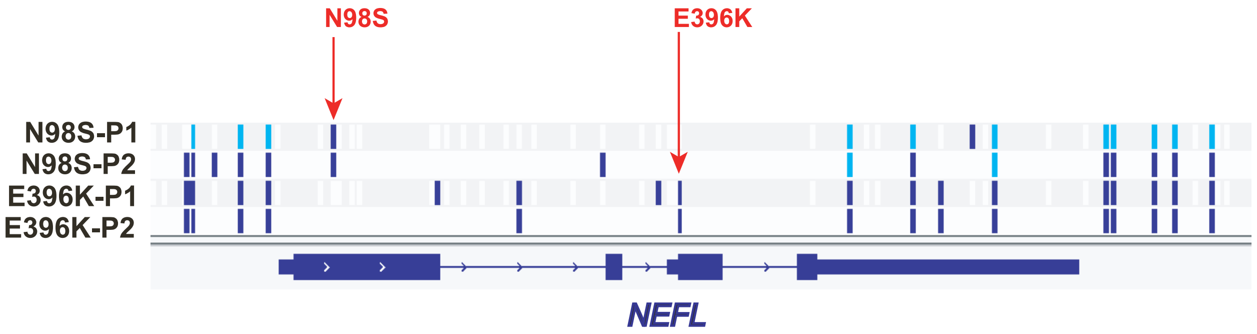

Figure S4: Panel of CMT2E patient iPSC lines with variants flanking the *NEFL* coding region. Whole genome sequencing was performed from patient-derived iPSC. Dark blue lines indicate heterozygous variants and light blue lines indicate homozygous variants. Causative missense mutations are annotated with red arrows. Images generated from vcf files using Integrative Genomics Viewer[1].

1. Robinson, J.T., Thorvaldsdóttir, H., Winckler, W., Guttman, M., Lander, E.S., Getz, G., and Mesirov, J.P. (2011). Integrative genomics viewer. Nat. Biotechnol. *29*, 24–26. 10.1038/nbt.1754.

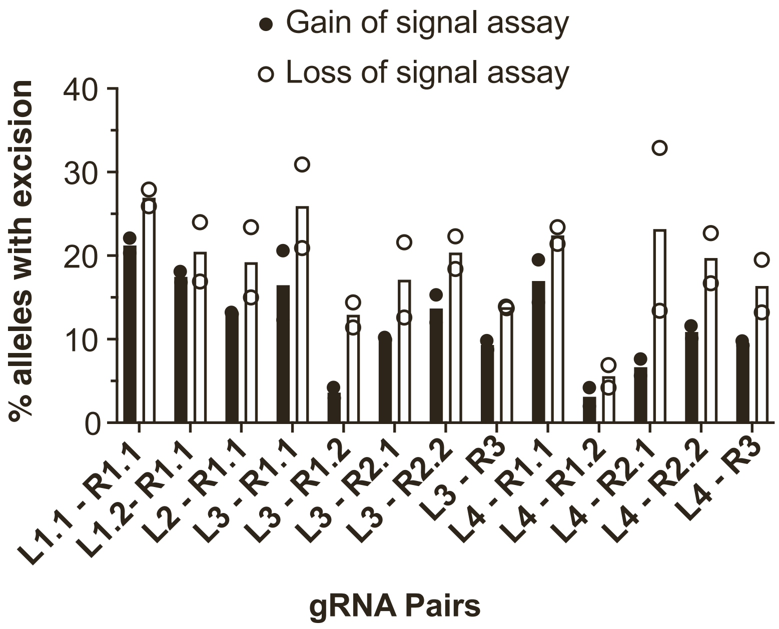

Figure S5: Comparison of ddPCR assays to measure excision by gain or loss of signal. N98S-P2 iPSC were nucleofected with different pairs of gRNA-HiFiCas9 RNP in duplicate, followed by gDNA extraction 4 days later. Excision was quantified for each sample using the assay shown in Figure 1E designed to detect the specific expected excision event (gain of signal, filled circles) and separately using an *NEFL* internal primer/probe assay that would be disrupted by gene excision or any event leading to loss of the genomic locus (loss of signal, open circles). Both assays were normalized to an internal RPP30 genomic copy number assay. For the loss of signal assay, percent excision was calculated by subtracting the normalized *NEFL* signal for each edited sample from unedited N98S-P2 control samples. Bar graphs represent mean values with data points indicating independent duplicate transfections.

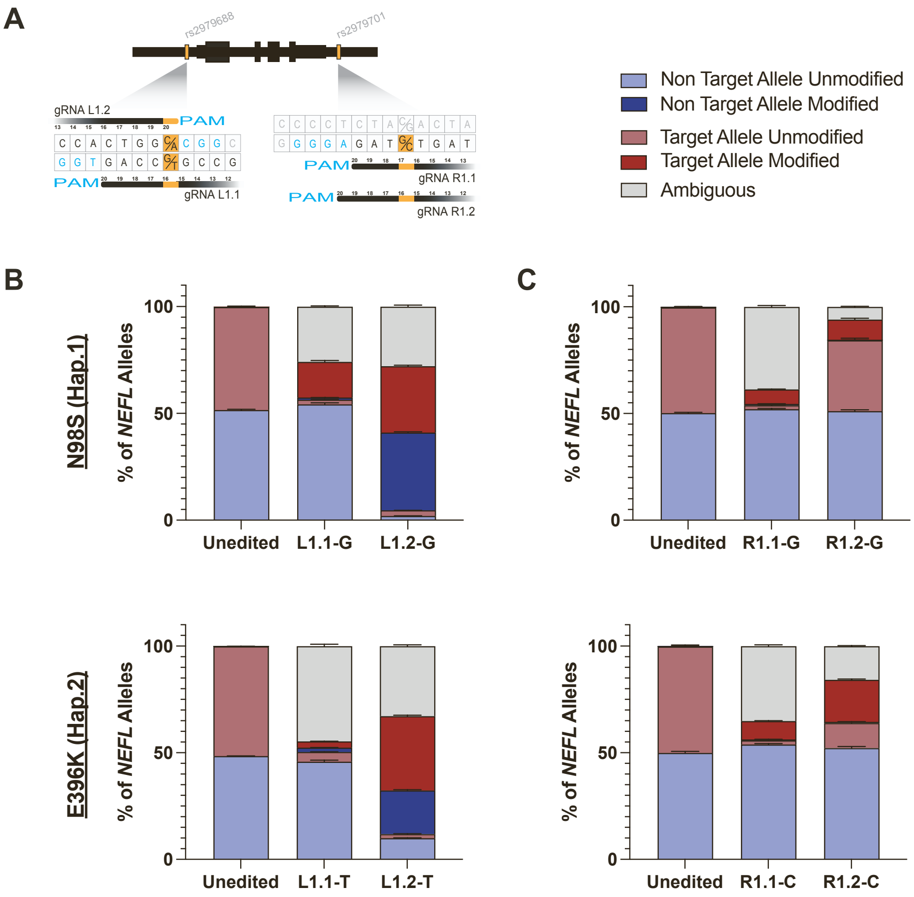

Figure S6: Analysis of allele-specific editing with single gRNA. (A) Schematic of sequences targeted by SNP-specific gRNAs with variants in orange and the associated PAM in blue. **(B,C)** N98S-P2 (top) and E396K-P2 (bottom) iPSC were transfected with the indicated allele-specific gRNA-HiFiCas9 RNPs followed by quantification of editing outcomes by NGS amplicon sequencing and CRISPResso2 analysis. Ambiguous editing events cannot be assigned to either allele due to the deletion of the variant nucleotide. Bar graphs represent the mean +/- S.E.M. of independent transfections (n = 3).

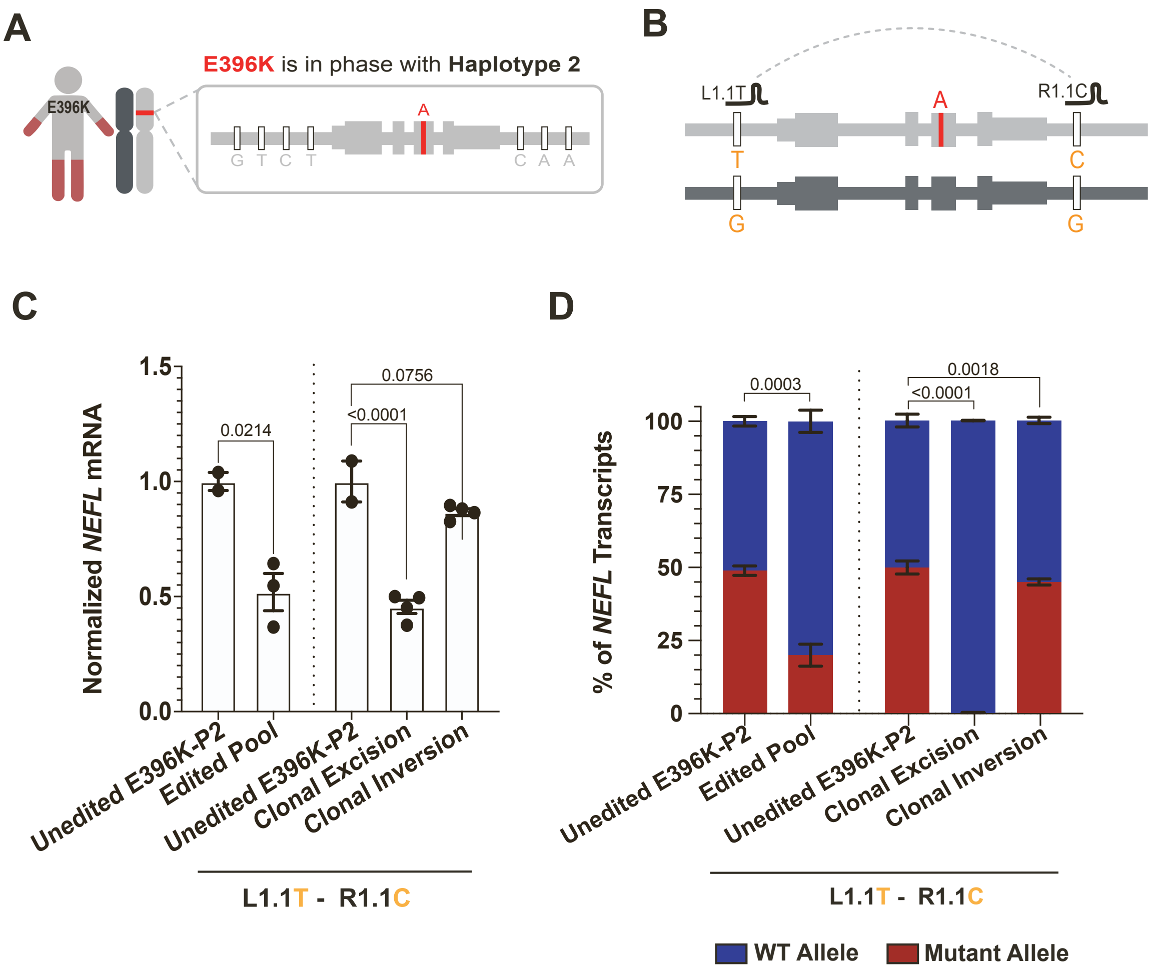

Figure S7: Analysis of *NEFL* gene expression after haplotype editing in E396K-P2 i^{3}LMNs. **(A)** Schematic of phasing of E396K mutation with haplotype 2 **(B)** Schematic of SNP-targeting gRNA pair for E396K haplotype editing. Edited and unedited iPSCs from E396K-P2 were differentiated into i^{3}LMNs, and RNA was extracted on Day 7. **(C)** Total *NEFL* expression was measured by quantitative RT-ddPCR relative to *GAPDH* and normalized to the unedited control. **(D)** Relative expression of wildtype and mutant alleles was measured by allele discrimination ddPCR. Bar graphs represent mean +/- S.E.M. normalized to unedited control, with each data point representing an independent well of i^{3}LMNs (n = 2 – 4). Dashed vertical lines indicate separate experiments for mixed population of edited cells versus clones with specific editing outcomes. Comparisons were performed by unpaired two-tailed T-test; multiple comparisons were performed by one-way ANOVA with Dunnett's post-test.

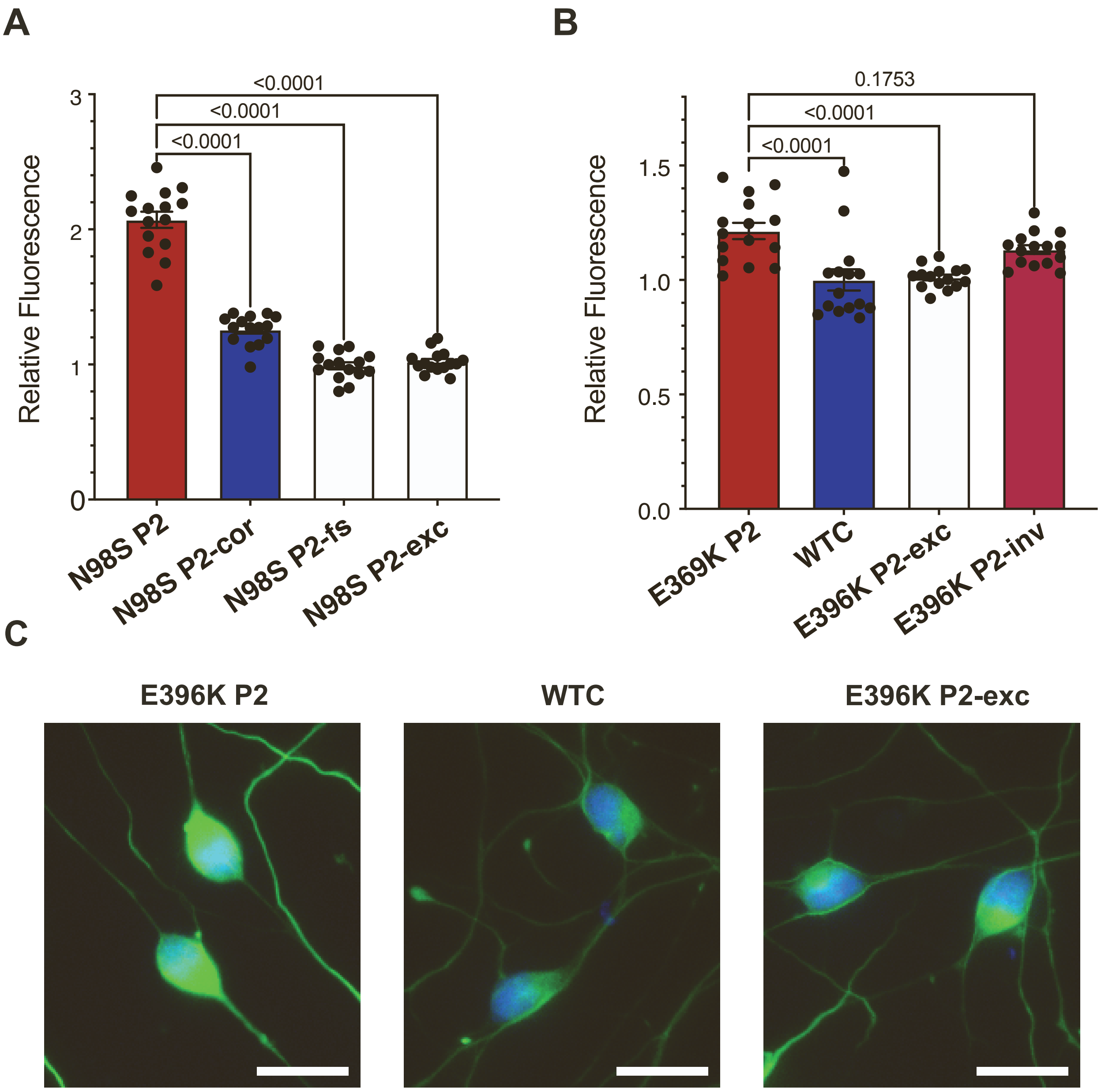

Figure S8: NfL accumulation in cell bodies of control and clonal edited i[3]LMNs.

Quantification of mean NF-L fluorescence intensity in inferred cell bodies in day 7 clonal edited i[3]LMNs derived from **(A)** N98S-P2 and **(B)** E396K-P2. Bar graph represents mean +/- S.E.M. of 15 independent wells of i[3]LMNs with each data point representing the mean NfL intensity from 5 images per well. Statistical comparisons by one-way ANOVA with Dunnett's post-test. **(C)** Representative images of day 7 i[3]LMNs stained with anti-NF-L (green) and anti-HB9 (blue). Scale bars = 20 µM.

Cell Body CellProfiler Pipeline

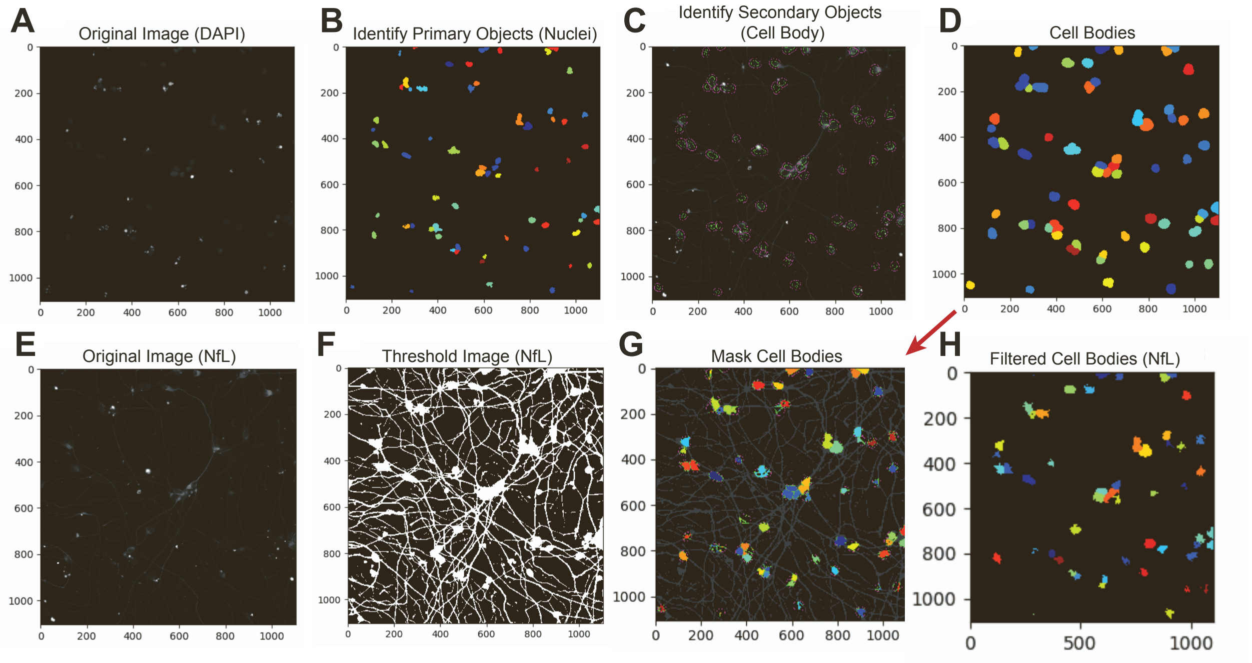

Figure S9: CellProfiler pipeline for measuring NfL intensity in i³LMN cell bodies. **(A-B)** Nuclei (primary objects) were identified from the DAPI-stained image channel. **(C-D)** Cell bodies (secondary objects) were inferred by extending the area of DAPI+ signal by 10 pixels. **(E-F)** The NfL-stained image channel was thresholded and then **(G)** masked with the inferred cell bodies. **(H)** The cell bodies were then filtered by shape and compactness to remove any incorrectly identified objects. Red arrow indicates the application of masks to images in other channels. The mean intensity in the cell body-masked NfL-stained image channel was measured to quantify NfL within cell bodies.

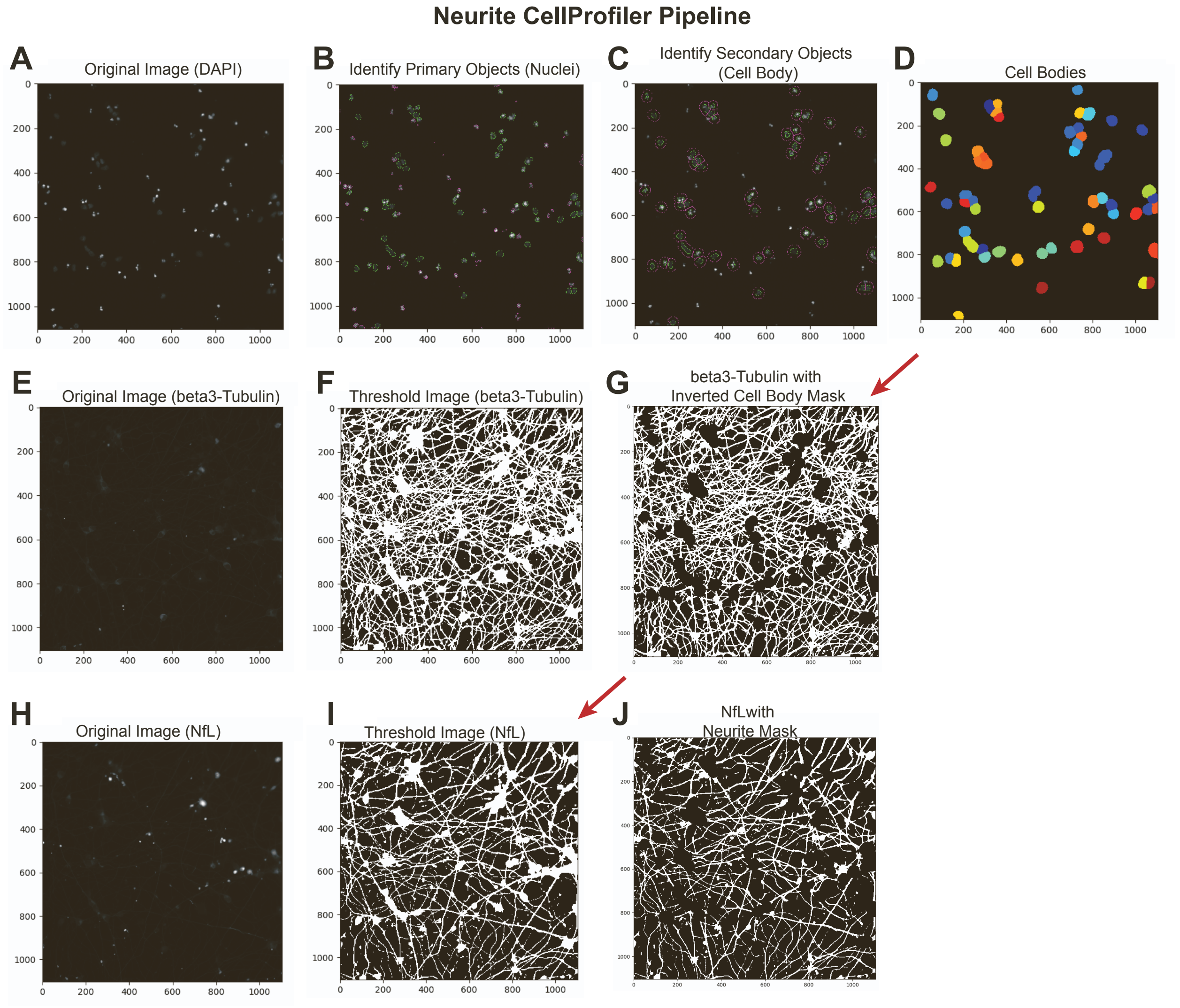

Figure S10: CellProfiler pipeline for measuring NF-L intensity in i^{3}LMN neurites.

(A-B) Nuclei (primary objects) were identified from the DAPI-stained image channel. **(C-D)** Cell bodies (secondary objects) were inferred by extending the area of the DAPI+ signal by 15 pixels. **(E-F)** The beta3-tubulin-stained image channel was thresholded, and **(G)** an inverted cell body mask was applied to define the region encompassed by neurites. **(H-I)** The NfL-stained image channel was then thresholded and **(J)** masked with the beta3-tubulin neurite image. Red arrows indicate the application of masks to images in other channels. Total intensity in the neurite-masked NfL-stained image channel was calculated and divided by the beta3-tubulin-positive neurite area to normalize for variation in neurite density.

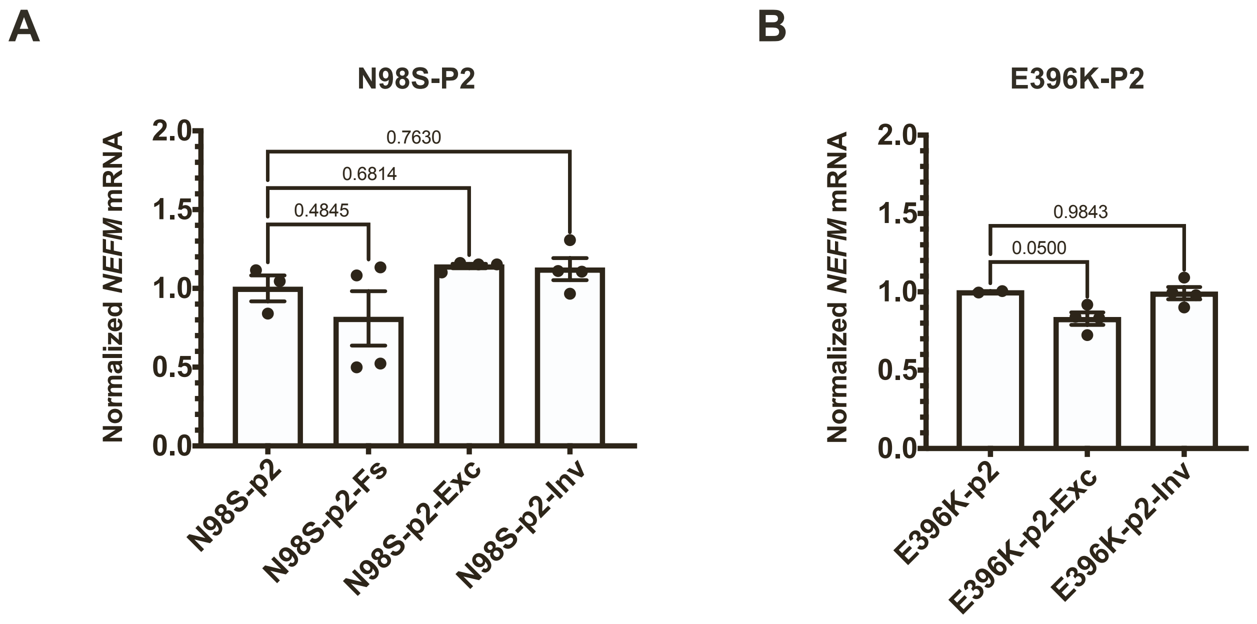

Figure S11: Total *NEFM* expression in edited i^{3}LMNs. Edited iPSCs from **(A)** N98S-P2 and **(B)** E396K-P2 were differentiated into i^{3}LMNs, and RNA was extracted on day 7. Total *NEFM* expression was measured by quantitative RT-ddPCR relative to *GAPDH* and normalized to unedited controls. Bar graphs represent mean +/- S.E.M. normalized to unedited control, with each data point representing an independent well of i^{3}LMNs (n = 2 – 4). Multiple comparisons were performed by one-way ANOVA with Dunnett's post-test.

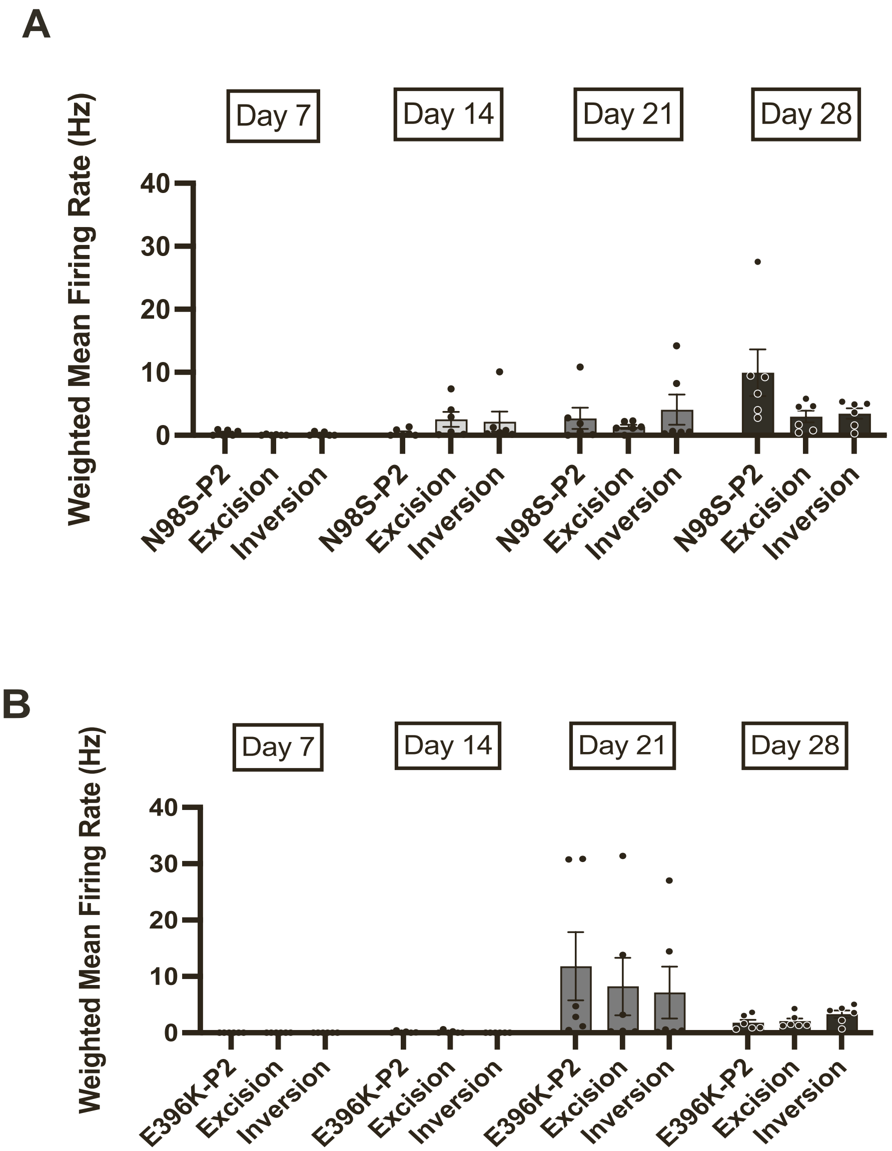

Figure S12: Total *NEFL* gene excision and inversion do not impair i^3LMN electrophysiology. Day 3 i^3LMNs were seeded on multi-electrode array plates to measure spontaneous action potentials on days 7, 14, 21, and 28 for **(A)** N98S-P2 and **(B)** E396K-P2 series of edited i^3LMN. Bar graphs represent mean +/- S.E.M. with each data point representing the mean firing rate from an independent well of i^3LMN (n = 6). Two-way ANOVA demonstrated a statistically significant effect of the differentiation day (N98S-P2 series: p = 0.0009, E396K-P2 series: p = 0.0002) but no significant difference between cell lines (N98S-P2 series: p = 0.3258, E396K-P2 series: p = 0.8814).

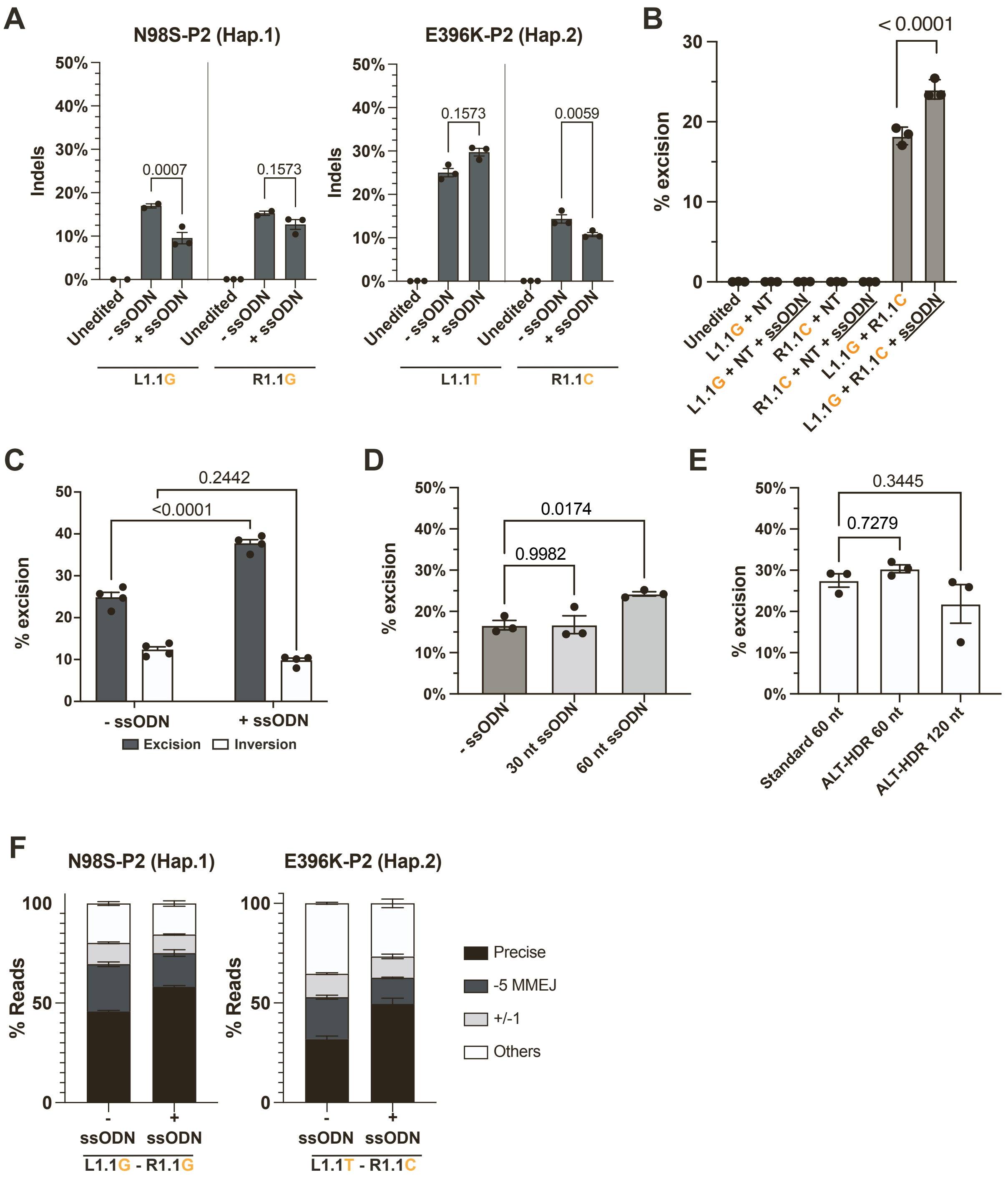

Figure S13: Effect of bridging ssODN on editing outcomes in iPSCs. (A) N98S-P2 and E396K-P2 iPSCs were nucleofected with L1.1 and R1.1 RNP with and without the addition of a 60 nt single-strand oligonucleotide donor (ssODN). PCR amplicons spanning L1.1 and R1.1 target sites were analyzed by NGS amplicon sequencing to quantify indels on alleles that were

not edited by excision or inversion. **(B)** E396K-P2 iPSC were nucleofected with L1.1 and R1.1 RNP, and with replacement of L1.1 or R1.1 with a non-targeting (NT) gRNA, with and without ssODN. Excision frequency was measured by ddPCR. **(C)** N98S-P2 iPSCs were transfected with L1.1 + BA1 RNP with and without ssODN. Excision and inversion frequency were measured by ddPCR. **(D,E)** E396K-P2 iPSCs were transfected with L1.1 + R1.1 RNP and ssODN of different lengths and chemistries. Excision was measured by ddPCR. Comparison of 30 and 60 nt unmodified ssODN shown in (D). Comparison of unmodified (60 nt) and ALT-HDR (Integrated DNA Technologies) modified (60 and 120 nt) ssODN shown in (E). For all experiments, gDNA was collected four days after nucleofection. For (A-E) bar graphs represent mean +/- S.E.M. with individual data points representing independent transfections (n = 3 – 4). Multiple comparisons were done by one- or two-way ANOVA with (A,B) Sidak's, (C) Tukey's, and (D,E) Dunnet's post-tests. **(F)** N98S-P2 (left) or E396K-P2 (right) iPSC were nucleofected with L1.1 and R1.1 RNP with and without the addition of a 60 nt single-strand oligonucleotide donor (ssODN). Excision events were amplified by PCR and sequenced by NGS. Outcomes were categorized as the expected excision repair (precise), 5-nucleotide larger than expected excision which was presumed microhomology-mediated (-5 MMEJ), single nucleotide insertion or deletion at the excision junction (+/-1), or numerous other rare variations (other). Statistical comparisons for the effect of ssODN on each category was performed by two-way ANOVA followed by Sidak's post-test as follows:

N98S-P2 - precise: $p < 0.0001$, -5 MMEJ: $p = 0.0008$, +/-1: $p = 0.8281$

E396K-P2 - precise: $p < 0.0001$, -5 MMEJ: $p = 0.0072$, +/-1: $p = 0.9777$

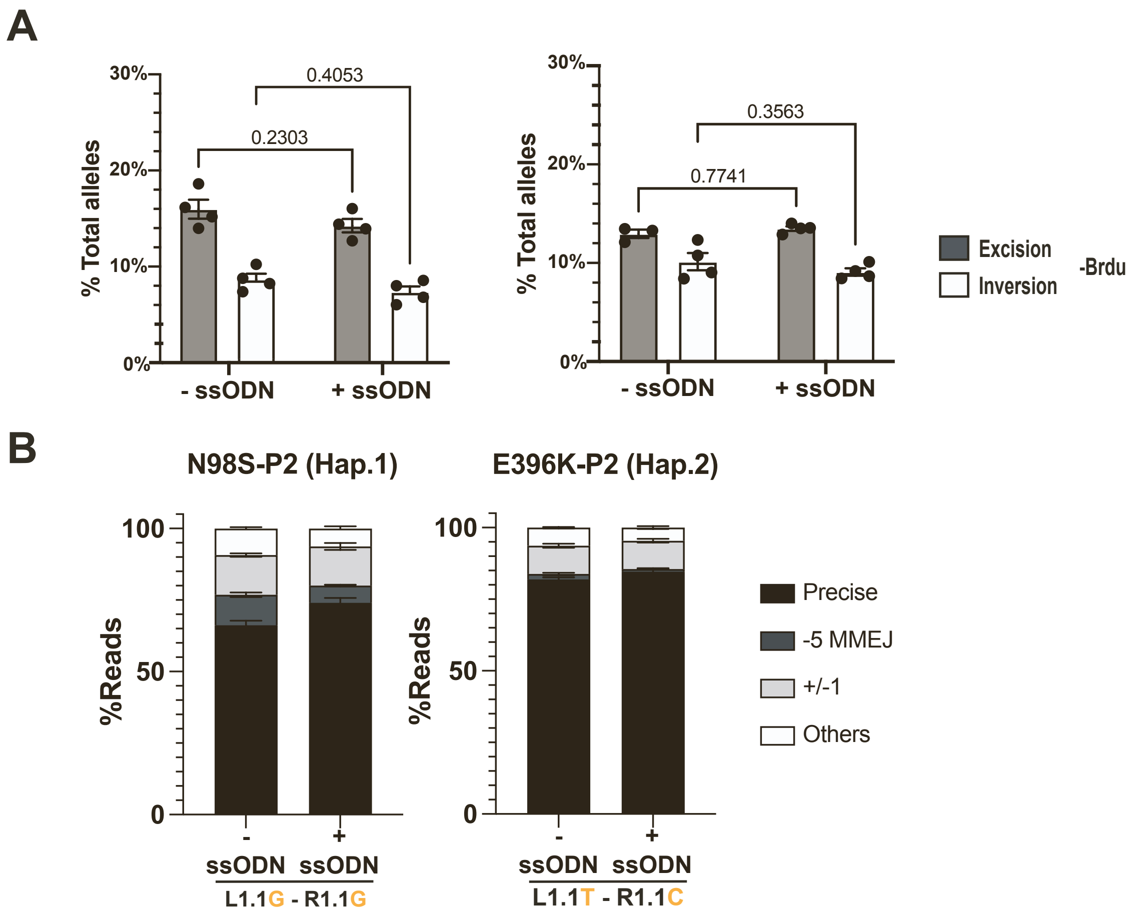

Figure S14: Effect of bridging ssODN on editing outcomes in day 3 i^{3}LMNs. (A) N98S-P2 (left) and E396K-P2 (right) day 3 i^{3}LMNs were nucleofected with L1.1 and R1.1 RNP with and without the addition of a 60 nt single-strand oligonucleotide donor (ssODN). Brdu was excluded from the media from days 3-4. Excision and inversion frequency were measured by ddPCR. Multiple comparisons were performed by two-way ANOVA with Sidak's post-test. **(B)** Excision events were amplified by PCR and sequenced by NGS. Outcomes were categorized as the expected excision repair (precise), 5-nucleotide larger than expected excision which was presumed microhomology-mediated (-5 MMEJ), single nucleotide insertion or deletion at the excision junction (+/-1), or numerous other rare variations (other). Bar graphs represent mean +/- S.E.M. of independent transfections (n = 4). Statistical comparisons for the effect of ssODN on each category was performed by two-way ANOVA followed by Sidak's post-test as follows: N98S-P2 - precise: $p < 0.0001$, -5 MMEJ: $p = 0.0169$, +/-1: $p = 0.9999$

E396K-P2 - precise: $p < 0.0299$, -5 MMEJ: $p = 0.7560$, +/-1: $p > 0.9999$

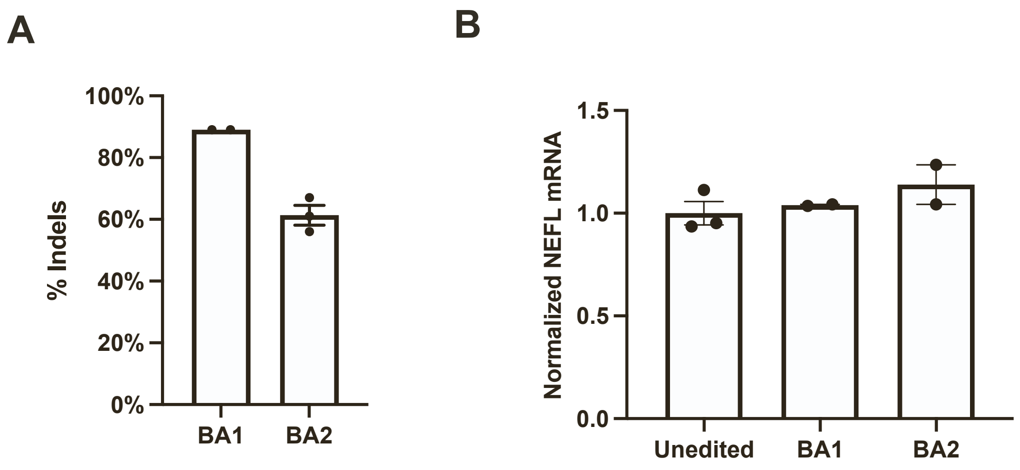

Figure S15: Evaluation of indels produced by biallelic gRNAs targeting *NEFL* intron 1. **(A)** WTC iPSCs were nucleofected with BA1 or BA2 RNP followed by quantification of indels by Sanger sequencing and ICE analysis. Bar graphs represent mean +/- S.E.M. with individual data points representing values from independent transfections (n = 2 – 3). **(B)** Total *NEFL* expression in day 7 i^3LMN differentiated from the edited populations shown in (A), as measured by quantitative RT-ddPCR and normalized to *GAPDH*. Bar graphs represent mean +/- S.E.M with individual data points representing independent wells of i^3LMNs (n = 2 – 3).

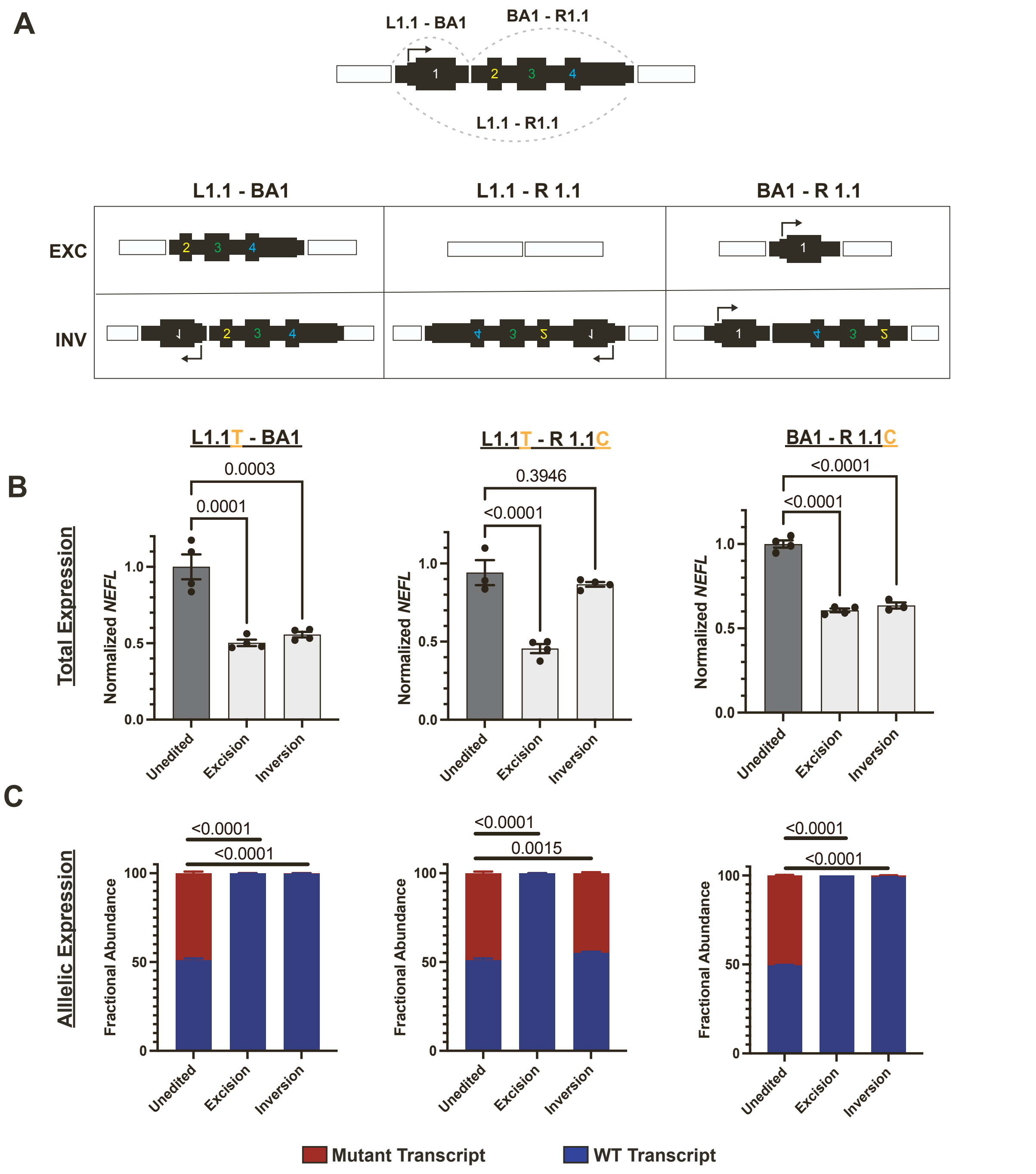

Figure S16: Clonal analysis of editing outcomes reveals variable contributions of excision and inversion to gene inactivation. (A) Schematic of predicted editing outcomes with various combinations of L1.1, R1.1 and intronic biallelic (BA1) gRNAs. E396K-P2 was transfected with each RNP pair and clonal iPSC lines were isolated with each predicted outcome and differentiated into i^{3}LMNs. RNA was isolated on day 7 and measured by

quantitative RT-ddPCR. **(B)** Total *NEFL* expression relative to *GAPDH* and normalized to unedited control. **(C)** Relative allelic expression via allele discrimination ddPCR using a heterozygous SNP in the 3' UTR (rs2976439). Bar graphs represent mean +/- S.E.M. with each data point representing an independent well of i^3LMNs (n = 4). Multiple comparisons were performed by one-way ANOVA followed by Dunnett's post-test.

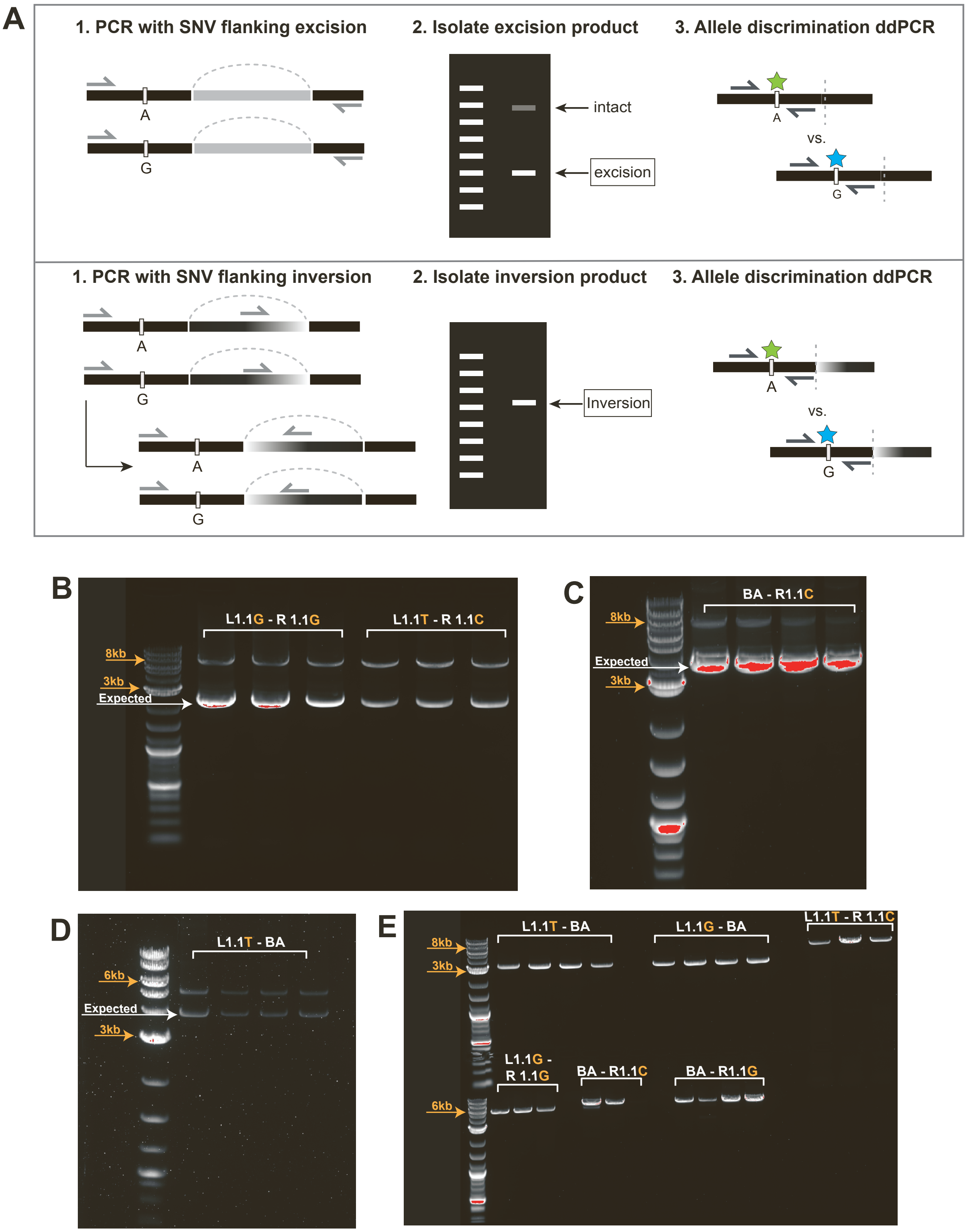
A
1. PCR with SNV flanking excision
2. Isolate excision product
3. Allele discrimination ddPCR
A
G
intact
excision
A
vs.
G
1. PCR with SNV flanking inversion
2. Isolate inversion product
3. Allele discrimination ddPCR
A
G
A
G
Inversion
A
vs.
G
B
L1.1G - R 1.1G
L1.1T - R 1.1C
8kb
3kb
Expected
C
BA - R1.1C
8kb
Expected
3kb
D
L1.1T - BA
6kb
Expected
3kb
E
L1.1T - BA
L1.1G - BA
L1.1T - R 1.1C
8kb
3kb
L1.1G -
R 1.1G
BA - R1.1C
BA - R1.1G
6kb

Figure S17: Multi-step PCR/ddPCR assay to measure allele-specificity of excision and inversion. (A) Assay workflow - 1: Schematic of PCR designed to amplify excised (top) or inverted (bottom) alleles with flanking heterozygous variant. 2: Gel purification to isolate excision or inversion products. 3: Schematic of allele discrimination ddPCR used to quantify specificity. **(B-D)** Examples of gel electrophoresis of PCR products used for excision specificity. **(E)** Examples of gel electrophoresis of PCR products used for inversion specificity.

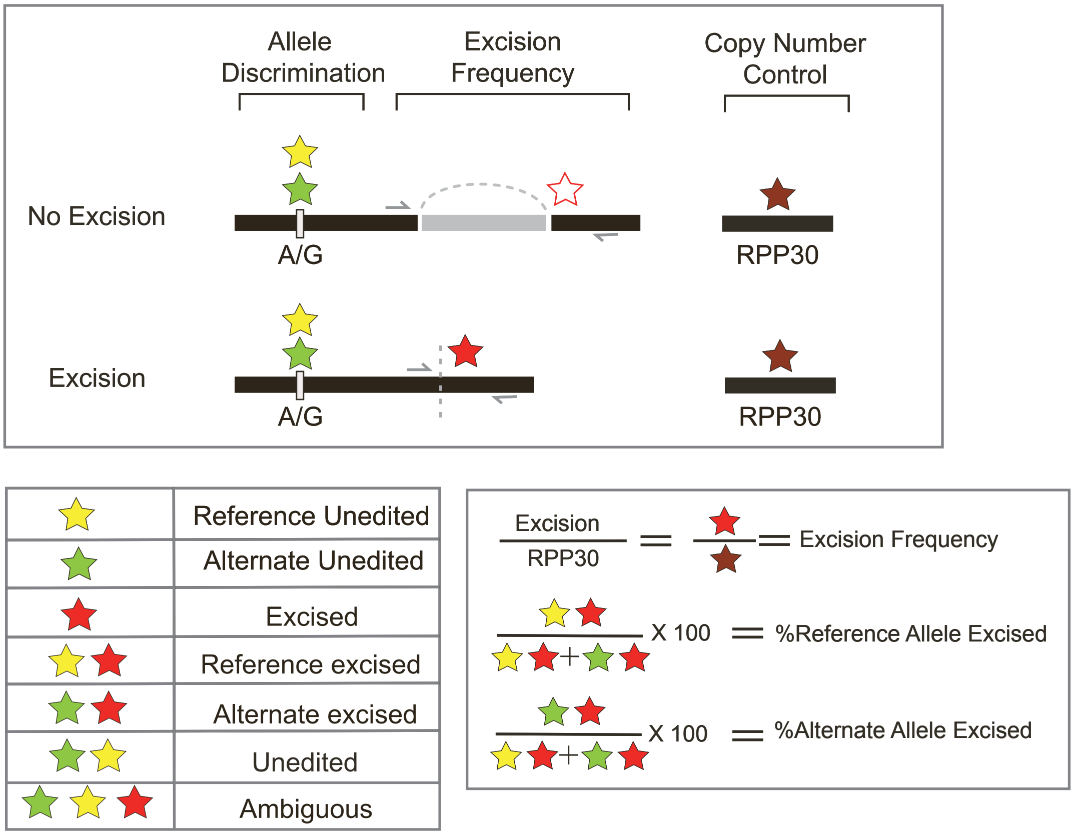

Figure S18: Single-step assay to measure excision frequency and specificity via multiplexed four-color digital PCR. Top: Schematic of multiplexed assay design with four probes (Green = FAM, Yellow = Hex, Red = ROX, Maroon = Cy5). **Bottom-left:** Partition occupancy observed for excision specificity measurements. **Bottom-right**: Calculation used to quantify excision specificity and frequency.

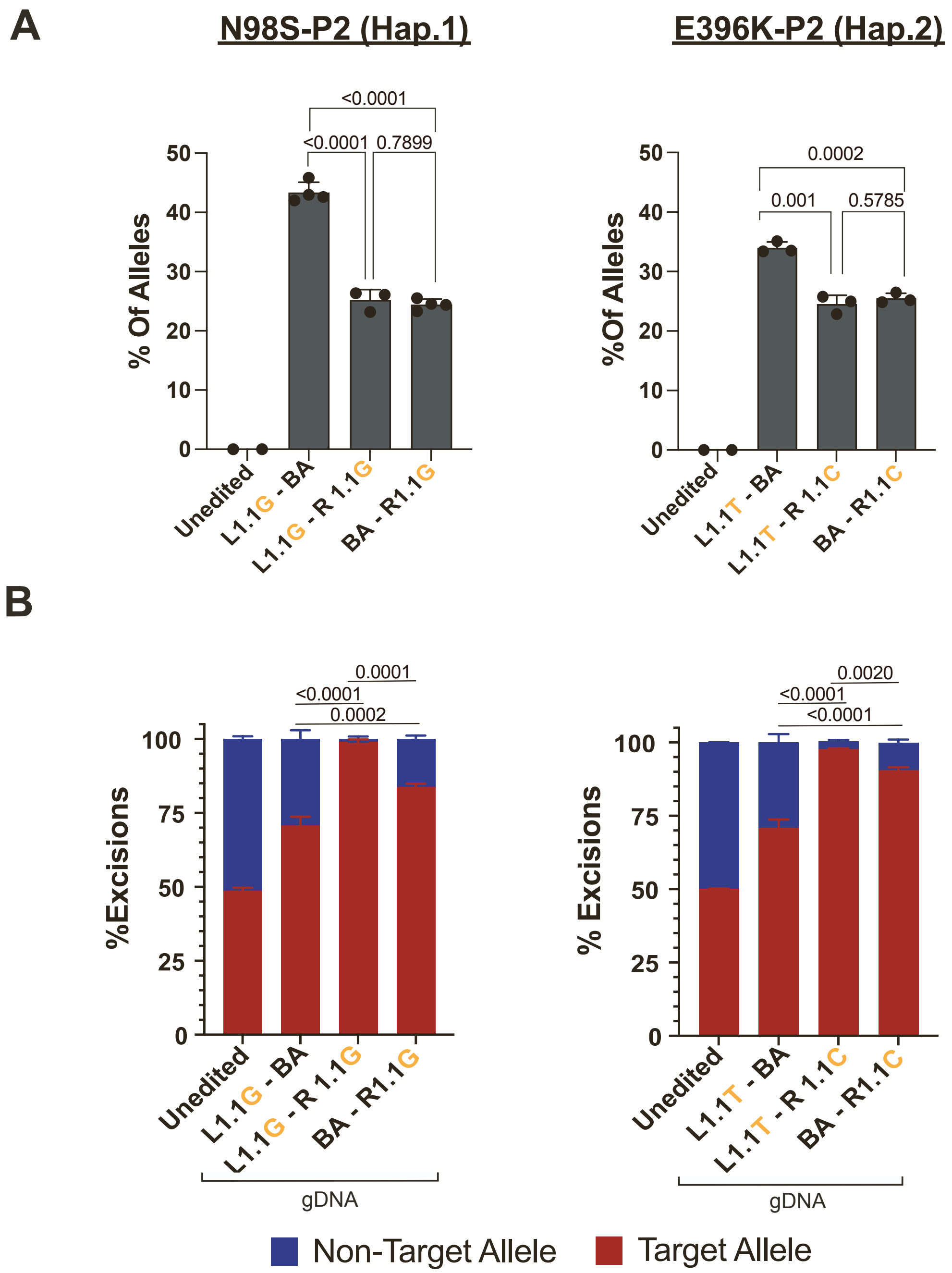

Figure S19: Quantification of excision frequency and specificity via multiplexed four-color digital PCR. The same samples from Figure 5 were assayed using the alternate method described in Figure S18. **(A)** Excision frequency as measured by the ratio of excision (ROX+) signal over RRP30 (Cy5+) signal. **(B)** Excision specificity measured via dPCR allele-discrimination assay for a heterozygous SNP (rs2979685, ref = HEX, alt = FAM) located 5' of

NEFL. Unedited control represents the fractional abundance of HEX vs. FAM in ROX-negative partitions to demonstrate equal abundance of the two alleles at baseline, while edited samples represent the fractional abundance of HEX vs. FAM in ROX-positive partitions that contain an excision event. Bar graphs represent mean +/- S.E.M. with individual data points representing independent transfections (n = 3 – 4). Multiple comparisons were performed by one-way ANOVA followed by Tukey's post-test.

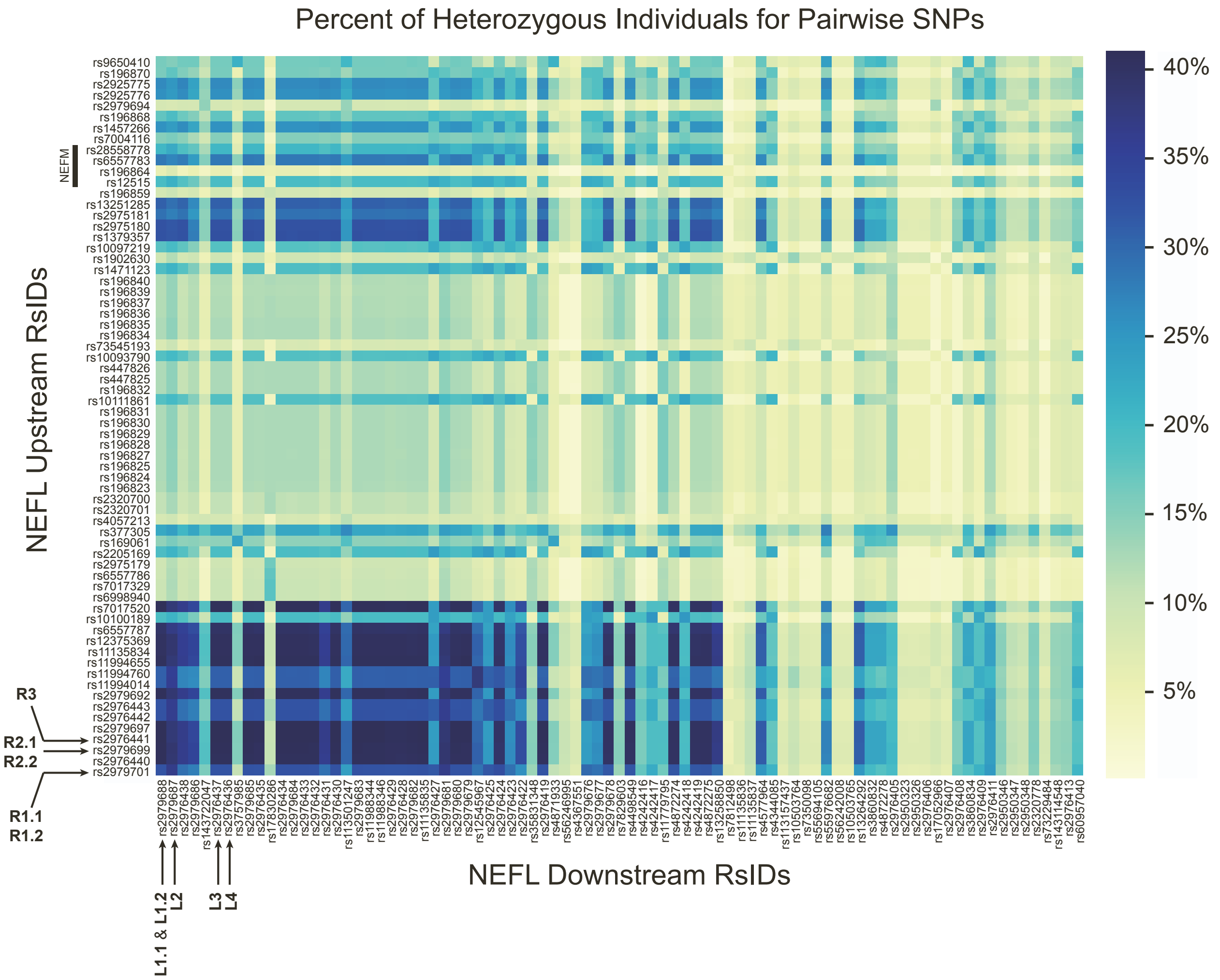

Figure S20: Percentage of individuals heterozygous for SNP pairs flanking *NEFL.* Variant data from 1000 Genomes phase 3, starting with minor allele frequency 0.1-0.9 in global population and within 50 kb of the *NEFL* gene**.** Horizontal axis indicates SNPs downstream (3') of *NEFL* increasing in linear distance from left to right. Vertical axis indicates SNPs upstream (5') of *NEFL* increasing in linear distance from bottom to top. The location of *NEFM* (upstream of *NEFL*) is annotated with a line along the vertical axis. Darker blue colors indicate a higher percentage of heterozygous individuals. Arrows indicate the SNPs targeted by gRNAs in this study. Note that *NEFL* is transcribed right to left from the bottom strand in the reference genome, opposite from our gRNA naming convention.

Table S1: Genomic off-target analysis

gRNA	#	chr	start (hg38)	End (hg38)	Strand	CFD score	Description	Results: N98S-P2 exc	Results: N98S-P2 inv
R1.1G	1	10	78632335	78632357	-	0.49777778	exon:KCNMA1	No mutations	No mutations
R1.1G	2	3	40733428	40733450	+	0.3799606	intergenic:RP11-528N21.1-HMGN2P24	No mutations	No mutations
R1.1G	3	5	8208980	8209002	+	0.34821429	intergenic:RP11-215I16.1-RP11-480D4.1	No mutations	No mutations
R1.1G	4	6	18441294	18441316	+	0.31533434	intron:RNF144B	No mutations	No mutations
R1.1G	5	8	48092200	48092222	+	0.26848498	intergenic:NDUFA5P12-RP11-1134I14.4	No mutations	No mutations
R1.1G	6	11	79198180	79198202	-	0.38787879	intergenic:TENM4-RP11-258O13.1	No mutations	No mutations
R1.1G	7	12	34271299	34271321	+	0.38956522	intergenic:RP11-847H18.3-RP11-313F23.3	Cell line has a 5 bp variant. No other mutations detected.	Cell line has a 5 bp variant. No other mutations detected.
R1.1G	8	4	72155681	72155703	+	0.38181818	intergenic:SLC4A4-RP11-1J11.1	No mutations	No mutations
R1.1G	9	5	133553321	133553343	+	0.36363636	intergenic:CTD-2410N18.4/CDKL3/PPP2CA-PPP2CA	No mutations	No mutations
R1.1G	10	6	68788813	68788835	-	0.32223776	intergenic:RP11-542F9.1-RP11-406O16.1	No mutations	No mutations
R1.1G	11	4	189766672	189766694	-	0.30198176	intergenic:RP11-756P10.2-RP11-818C3.1	No mutations	No mutations
**gRNA**	**#**	**chr**	**start (hg38)**	**End (hg38)**	**Strand**	**CFD score**	**Description**	**Results: E396K-P2 exc**	**Results: E396K-P2 inv**
L1.1T	1	5	2501128	2501150	+	0.4982699	intergenic:Y_RNA-RP11-129I19.2	No mutations	No mutations
L1.1T	2	11	43929675	43929697	-	0.29658922	intergenic:ALKBH3-ALKBH3-AS1	No mutations	No mutations
L1.1T	3	13	102135513	102135535	+	0.27731092	intron:ITGBL1	No mutations	No mutations
R1.1C	4	12	57160521	57160543	+	0.46928467	intron:HSD17B6	No mutations	No mutations
R1.1C	5	10	92818471	92818493	-	0.45389474	intergenic:DDX18P6-LINC00502	No mutations	No mutations
R1.1C	6	3	156692992	156693014	-	0.35	intergenic:LEKR1-KLF3P2	No mutations	No mutations
R1.1C	7	3	80744740	80744762	-	0.27575758	intergenic:RP11-481N16.1-RP11-47P18.1	PCR failed	PCR failed
R1.1C	8	3	4047631	4047653	+	0.44426162	intergenic:PNPT1P1-SUMF1	No mutations	No mutations

R1.1C	9	2	57033442	57033464	+	0.56384439	intergenic:AC008173.1-snoU13	Homozygous alternate SNP. No other mutations detected.	Homozygous alternate SNP. No other mutations detected.
R1.1C	10	5	67112973	67112995	-	0.51948052	intergenic:RP11-434D9.1-RP11-83M16.6	No mutations	No mutations
R1.1C	11	2	56267847	56267869	+	0.39751553	intron:AC011306.2	No mutations	No mutations
R1.1C	12	4	1103514	1103536	-	0.37151703	intron:RNF212	No mutations	No mutations
R1.1C	13	1	173767314	173767336	+	0.34208145	intergenic:RN7SKP160-CENPL	No mutations	No mutations
R1.1C	14	1	159346828	159346850	-	0.32814779	intergenic:OR10J8P-OR10J9P	No mutations	No mutations
R1.1C	15	4	99726082	99726104	-	0.26526316	intergenic:BTF3P13-EIF4E	No mutations	No mutations
R1.1C	16	10	54100207	54100229	+	0.26352941	intergenic:DKK1-RP11-346D6.4	No mutations	No mutations
**gRNA**	**#**	**chr**	**start (hg38)**	**End (hg38)**	**Strand**	**CFD score**	**Description**	**Results: N98S-P2 FS**	**Results: N98S-P2 Cor**
N98S	1	1	148316436	148316458	+	0.7143	intergenic: RP11-495P10.8/RP11-495P10.5/RP11-495P10.7-RP11-495P10.3	Sequencing Failed	Sequencing Failed
N98S	2	12	5752303	5752303	-	0.4412	intron: ANO2	PCR failed	PCR failed
N98S	3	10	75661072	75661094	-	0.3956	intergenic: RP11-310J24.3-RP11-367B6.2	No mutations	No mutations
N98S	4	20	48137627	48137649	+	0.3652	intergenic: AL139351.1-LINC00494	No mutations	No mutations
N98S	5	18	39020019	39020041	-	0.3649	intergenic: RN7SKP182-RNU6-706P	No mutations	No mutations
N98S	6	4	3605528	3605550	-	0.3563	intergenic: LINC00955-RP3-368B9.2	No mutations	No mutations
N98S	7	9	65426450	65426472	+	0.3563	intergenic: FOXD4L5-Y_RNA	No mutations	No mutations
N98S	8	8	1925519	1925541	-	0.3545	intron: ARHGEF10	No mutations	No mutations
N98S	9	13	111494882	111494904	+	0.3254	intergenic: TEX29-RP11-65D24.2	No mutations	No mutations
N98S	10	8	120892179	120892201	-	0.3196	intergenic: RP11-713M15.2/SNTB1-RP11-369K17.1	No mutations	No mutations
N98S	11	9	14223924	14223946	+	0.279	intron: NFIB	No mutations	No mutations

Table S2: ssODN sequences for excisions

**L1.1G**	**BA**	CTTGGCTGCAGCAGCGCGCTGCCCCCACTGTTCCGCATTAAAGCTGCCCA GCCCTTGTTG
**L1.1G**	**R1.1G**	CCTTGGCTGCAGCAGCGCGCTGCCCCCACTCACTAACCTATCACAGAGTTA TAGTGAGAA
**BA**	**R1.1G**	GGGAGTGTGCTCCGTGCTGCTGCACCGGCGCACTAACCTATCACAGAGTTA TAGTGAGAA
**L1.1T**	**BA**	CTTGGCTGCAGCAGCGCGCTGCCCCCACTGTTCCGCATTAAAGCTGCCCAG CCCTTGTTG
**L1.1T**	**R1.1C**	CCTTGGCTGCAGCAGCGCGCTGCCCCCACTGACTAACCTATCACAGAGTTAT AGTGAGAA
**BA**	**R1.1C**	GGGAGTGTGCTCCGTGCTGCTGCACCGGCGGACTAACCTATCACAGAGTTAT AGTGAGAA

Table S3: Antibodies

**Name/Antigen**	**Fluorophore**	**Vendor**	**Catalog / Clone #**	**Dilution / *µL per 1E6 cells**
NfL	N/A	Millipore	AB9568	1:1000 for CX7, 1:500 for Keyence
Beta3-Tubulin	N/A	ThermoFisher	480011	1:250
HB9	N/A	DSHB	81.5c10	1:200
Goat anti-rabbit	AF488	Invitrogen	A11034	1:500
Goat anti-mouse	AF594	Invitrogen	A11032	1:500
Live/Dead	NIR	Thermofisher	L34957A	1:2500
Tra-1-60	BV421	BD	562711	0.625*
SSEA-3	FITC	BD	560236	5*
Tra-1-81	PE	BD	560161	5*
SSEA-4	PE-Cy7	Biolegend	330420	0.625*
SSEA-1	APC-Cy7	Biolegend	323048	0.625*
Nanog	AF647	Biolegend	674210	2*
Oct ¾	BV421	BD	560306	5*
Sox2	AF488	BD	245610	5*

Table S4: CLYBL hNIL genotyping junction PCR primer sequences

**Junction PCR Primers for hNIL CLYBL integration and Post-CRE Junction PCR Primers**	
hNIL CLYBL 5' Junction F	CAGACAAGTCAGTAGGGCCA
hNIL CLYBL 5' Junction R	AGAAGACTTCCTCTGCCCTC

hNIL CLYBL 3' Junction F	CACCAGCAACCTGACGTTTT
hNIL CLYBL 3' Junction R	TTTTATAGGCGCCCACCGTA
CLYBL WT F	TGACTAAACACTGTGCCCCA
CLYBL WT R	AGGCAGGATGAATTGGTGGA
CF28	CCTCAGCCCAGTTTCCACTTG
CF29	GGCTATGAACTAATGACCCCGT

Table S5: hNIL copy number custom 20x ddPCR primer/ probe mixes

**Name**	**F Primer**	**R Primer**	**Probe**
Neomycin Assay	CATGGCTGATGCAATGCG	TCGCTTGGTGGTCGAATG	CGCTTGATCCGGCTACCTGCC
TRE3G Assay	TACGGTGGGCGCCTATAA	AGTGGTACGGAAAGTTGGTATAAG	AGATCGCCTGGAGCAATTCCACAA

Table S6: Sequences of gRNA genomic targets

**sgRNA Name**	**Target**	**Sequence**	**Allele Specific (AS) or Biallelic (BA)**
N98S	N98S	AGCTGGCGAAGCGGTCACTG	AS
E396K	E396K	CAGGAAACTCTTGGAAGGCA	AS
L1.1G	rs2979688	GATCACGGCACGCCGGCCAG	AS
L1.2G	rs2979688	AGCGCGCTGCCCCCACTGGC	AS
L2T	rs2979687	TCACGGGGTCTGGGCAATGC	AS
L3C	rs2976437	TCTACATATGGGTAATTGGG	AS
L4A	rs2976436	ACCCATATGTAGATGAAGCA	AS
R1.1G	rs2979701	TCTGTGATAGGTTAGTGTAG	AS
R1.2G	rs2979701	CTGTGATAGGTTAGTGTAGA	AS
R2.1T	rs2979699	TACCAGGGTGACTGGAGTGC	AS
R2.2T	rs2979699	TTCCAGCACTCCAGTCACCC	AS
R3G	rs2976441	GCTTAAATGTCATTCTCTAA	AS
L1.1T	rs2979688	GATCACGGCACGCCGTCCAG	AS

L1.2T	rs2979688	AGCGCGCTGCCCCCACTGGA	AS
R1.1C	rs2979701	TCTGTGATAGGTTAGTCTAG	AS
R1.2C	rs2979701	CTGTGATAGGTTAGTCTAGA	AS
BA1	Intron 1	GCAGCTTTAATGCGGAACGC	BA
BA2	Intron 1	CCTTTATTTAGTAGGTAGAC	BA
NT	Non-Targeting	GGCCAAACGTGCCCTGACGG	NT

Table S7: Digital PCR assays for excision and inversion frequency

**F primer**	**R primer**	**Probe**	**Excision Assay**
HW_ddXR_79_F	HW_ddXR_63_R	HW79	L3-R1.1, L3-R1.2, L4-R1.1, L4-R1.2
HW_ddXR_79_F	HW_ddXR_55_R	HW79	L3-R2.1, L3-R2.2, L4-R2.1, L4-R2.2
HW_ddXR_79_F	HW_ddXR_53_R	HW79	L3-R3, L4-R3
HW_ddXR_74_F	HW_ddXR_63_R	HW63	L2-R1.1
HW_ddXR_72_F	HW_ddXR_63_R	HW63	L1.1-R1.1, L1.2-R1.1
CM_ddXR_4790_F	CM_ddXR_11390_R	HW69	L1.1-R3, L1.2-R3
CM_ddXR_4590_F	CM_ddXR_11390_R	HW74	L2-R3
CM_ddXR_4790_F	CM_ddXR_11270_R	HW69	L1.1-R2.1, L1.1-R2.2, L1.2-R2.1, L1.2-R2.2
CM_ddXR_4590_F	CM_ddXR_11270_R	HW74	L2-R2.1, L2-R2.2
CM_ddXR_4890_F	CM_ddXR_10970_R	HW63	L1.1-R1.2, L1.2-R1.2
CM_ddXR_4650_F	CM_ddXR_10970_R	HW63	L2-R1.2
PD_ddXR_72/73F_anchored	PD_ddXR_HW15_R	PD_ddXR_72/63_probe	L1.1 - BA
HW_ddXR_15/63F	HW_ddXR_63_R	HW63	BA - R1.1
NEFL_EX4common_exc_F	NEFL_EX4common_exc_R	NEFLexon4	*NEFL* Loss of Signal

F primer	R primer	Probe	Inversion Assay
HW_ddXR_79_F	CM_iddXR_63_R	HW79	L3-R1.1, L3-R1.2, L4-R1.1, L4-R1.2
HW_ddXR_79_F	CM_iddXR_55_R	HW79	L3-R2.1, L3-R2.2, L4-R2.1, L4-R2.2
HW_ddXR_79_F	CM_iddXR_53_R	HW79	L3-R3, L4-R3
CM_iddXR_74_F	HW_ddXR_63_R	HW63	L2-R1.1
CM_NEFL_4996_R	HW_ddXR_63_R	HW63	L1.1-R1.1, L1.2-R1.1
CM_iddXR_11250_F	CM_iddXR_11430_R	HW13	L1.1-R3, L1.2-R3
CM_ddXR_4590_F	CM_iddXR_4840_R	HW74	L2-R3
CM_iddXR_11110_F	CM_iddXR_11310_R	HW13	L1.1-R2.1, L1.1-R2.2, L1.2-R2.1, L1.2-R2.2
CM_ddXR_4590_F	CM_iddXR_4800_R	HW74	L2-R2.1, L2-R2.2
CM_iddXR_10800_F	CM_iddXR_10950_R	HW63	L1.1-R1.2, L1.2-R1.2
CM_iddXR_10810_F	CM_iddXR_10950_R	HW63	L2-R1.2
PD_ddXR_72/73F_anchored	PD_iddXR_HW15_R	PD_ddXR_72/63_probe	L1.1 - BA
HW_iddXR_15/63F	HW_ddXR_63_R	HW63	BA - R1.1

Table S8: Specificity assays for inversions and excisions

Guide Pair	F Primer	R Primer	Allele discrimination assay	Assay Type
L1.1 - BA	NEFL_forASaround rs2979685	PD_7984_R	rs2979685	Excision Specificity
L1.1 - R1.1	NEFL_forASaround rs2979685	HW_ddXR_63_R	rs2979685	
BA - R1.1	NEFL_forASaround rs2979685	HW_ddXR_63_R	rs2979685	
L1.1 - BA	NEFL_forASaround rs2979685	HW_NEFL_5740_F	rs2979685	Inversion Specificity

L1.1 - R1.1	NEFL_forASaround rs2979685	BMJS_NEFL_7758F	rs2979685	
BA - R1.1	NEFL_forASaround rs2979685	HW_ddXR_63_R	rs2979685	

Table S9: Primer and probe sequences for excision and inversion frequency / specificity assays

**CATEGORY**	**NAME**	**SEQUENCE (5' → 3')**
Primer	CM_ddXR_4790_F	GGCATGGGATCTCAGAGAAA
Primer	CM_ddXR_11390_R	CAAAGAATTTGACCCACTAGAAGAG
Primer	CM_ddXR_4590_F	GAGGTTCCTGGTGGGAAA
Primer	CM_ddXR_11270_R	AAAGATGAGTGCTCCAGAAA
Primer	CM_ddXR_4890_F	CGCAGAATCCTCGCCTT
Primer	CM_ddXR_10970_R	CCCTGGGAGAAGGGTTAGA
Primer	CM_ddXR_4650_F	GAGGTGACGGGATACAGAAA
Primer	HW_ddXR_79_F	TATGCAGACTCACACACACTG
Primer	HW_ddXR_72_F	GCAGAATCCTCGCCTTGG
Primer	HW_ddXR_74_F	GGGCAACTTAAGGATCCAAGT
Primer	HW_ddXR_63_R	GTGGTGGCAGTATAAATTGAAAGA
Primer	HW_ddXR_55_R	ATCCTGTGACAGATGGGAGAA
Primer	HW_ddXR_53_R	TTCAAAGAATTTGACCCACTAGAAG
Primer	CM_iddXR_74_F	GCTCAGAGGGCCCTGATTTT
Primer	CM_iddXR_63_R	CTGTGGTCAGTGCCCCTTTT
Primer	CM_iddXR_55_R	CTGCCTAGTGCTGACTCCTG
Primer	CM_iddXR_53_R	CTCCACTTCCAGCACTCCAG
Primer	CM_NEFL_4996_R	CCGTTCTGCCACCCCTATTT
Primer	CM_iddXR_11250_F	GAGGATGGATGGCTGTGTG
Primer	CM_iddXR_11110_F	GAGGATGGATGGCTGTGTG
Primer	CM_iddXR_10800_F	TATTTATACGCCGGGAGGCT
Primer	CM_iddXR_10810_F	CCCTCACTCATTTCCCTTCTG
Primer	CM_iddXR_11430_R	AGATGCTAATGGCAAGAATCAA
Primer	CM_iddXR_4840_R	CTCCCATCTGTCACAGGATTT
Primer	CM_iddXR_11310_R	AATCTGAAGGGTCAGTAGGAAC
Primer	CM_iddXR_4800_R	CTGACTCCTGCCTAGTTCTCTA
Primer	CM_iddXR_10950_R	GTGGTGGCAGTATAAATTGAAAGAT
Primer	NEFL_EX4common_exc_F	GCATGGACCACGCTTATGA
Primer	NEFL_EX4common_exc_R	CACCGAAGGTTCAAAGGACTAT
Primer	PD_ddXR_72/73F_anchored	CCCTCTGAGCAAAGTGGAAA

Primer	HW_ddXR_15/63F	TGTAGTCTGGGAGTGTGCT
Primer	HW_iddXR_15/63F	CCCAATTCCCACGTCTTCC
Primer	NEFL_forASaroundrs2979685	GGCTGTCGTGGAGTATGAGG
Primer	PD_ddXR_HW15_R	CAGGTCAGTAGAGAGCTGAT
Primer	HW_ddXR_63_R	GTGGTGGCAGTATAAATTGAAAGA
Primer	PD_iddXR_HW15_R	TGTAGTCTGGGAGTGTGCT
Primer	PD_7984_R	CTCCTCTTGGACATGGCTGG
Primer	HW_ddXR_72_F	GCAGAATCCTCGCCTTGG
Primer	CM_NEFL_4996_R	CCGTTCTGCCACCCCTATTT
Primer	HW_NEFL_5740F	TCGACAGCTTGATGGACGAA
Primer	BMJS_NEFL_7758F	TCCTGCTTGCCTTTGTGTTTAG
Probe	HW79	TGAGGTTTGCAGGGAGCAGGTTAA
Probe	HW63	ACACCTCCATGTCTTAGATCCTTCCACA
Probe	HW13	CAGGCTGCGTCAGG
Probe	HW69	TCTGAGCAAAGTGGAAAGGACGACC
Probe	HW74	CTGCGAGGTGACGGGATACAGAAA
Probe	PD_ddXR_72/63_probe	CTGCGAGGTGACGGGATACAGAAA
Probe	NEFLexon4	ACGGCAATGTGAATCA

Table S10: Primer sequences for next generation sequencing and sanger sequencing

**Name**	**Sequence 5' → 3'**
BMJS_NGS_10733F	TCCCTACACGACGCTCTTCCGATCTcagctAgttctctttcagtattc cctctcc
BMJS_NGS_10965R	GTTCAGACGTGTGCTCTTCCGATCTcagctAggttagagtggtggc agtataaa
BMJS_NGS_4810F	TCCCTACACGACGCTCTTCCGATCTcagctAccctctgagcaaagt ggaaa
BMJS_NGS_5025R	GTTCAGACGTGTGCTCTTCCGATCTcagctAgaggatggatggct gtgtg
QC65_NGS-Fwd_NF-L1	CTTTCCCTACACGACGCTCTTCCGATCTNNNNaaagtggaaa ggacgaccgccg
QC70_NGS-Rev_NF-R1	GGAGTTCAGACGTGTGCTCTTCCGATCTggagaccaagagtag gccctggg

HW_NEFL_4388F	TCCCTATGTGTTAAGCAGCTC
HW_NEFL_5932R	GCGGCTCTTGAACCATTCCT
HW_NEFL_10113F	ACACGCCCCATGTATCCTTT
HW_ddXR_63R	GTGGTGGCAGTATAAATTGAAAGA
